# Evolution and Potential Function in Molluscs of Neuropeptide and Receptor Homologues of the Insect Allatostatins

**DOI:** 10.3389/fendo.2021.725022

**Published:** 2021-09-29

**Authors:** Zhi Li, João C. R. Cardoso, Maoxiao Peng, João P. S. Inácio, Deborah M. Power

**Affiliations:** ^1^ Comparative Endocrinology and Integrative Biology, Centre of Marine Sciences, Universidade do Algarve, Faro, Portugal; ^2^ International Research Center for Marine Biosciences, Ministry of Science and Technology, Shanghai Ocean University, Shanghai, China; ^3^ Key Laboratory of Exploration and Utilization of Aquatic Genetic Resources, Ministry of Education, Shanghai Ocean University, Shanghai, China

**Keywords:** allatostatins, GPCRs, molluscs, immunity, function

## Abstract

The allatostatins (ASTs), AST-A, AST-B and AST-C, have mainly been investigated in insects. They are a large group of small pleotropic alloregulatory neuropeptides that are unrelated in sequence and activate receptors of the rhodopsin G-protein coupled receptor family (GPCRs). The characteristics and functions of the homologue systems in the molluscs (Buccalin, MIP and AST-C-like), the second most diverse group of protostomes after the arthropods, and of high interest for evolutionary studies due to their less rearranged genomes remains to be explored. In the present study their evolution is deciphered in molluscs and putative functions assigned in bivalves through meta-analysis of transcriptomes and experiments. Homologues of the three arthropod AST-type peptide precursors were identified in molluscs and produce a larger number of mature peptides than in insects. The number of putative receptors were also distinct across mollusc species due to lineage and species-specific duplications. Our evolutionary analysis of the receptors identified for the first time in a mollusc, the cephalopod, GALR-like genes, which challenges the accepted paradigm that AST-AR/buccalin-Rs are the orthologues of vertebrate GALRs in protostomes. Tissue transcriptomes revealed the peptides, and their putative receptors have a widespread distribution in bivalves and in the bivalve *Mytilus galloprovincialis*, elements of the three peptide-receptor systems are highly abundant in the mantle an innate immune barrier tissue. Exposure of *M*. *galloprovincialis* to lipopolysaccharide or a marine pathogenic bacterium, *Vibrio harveyi*, provoked significant modifications in the expression of genes of the peptide precursor and receptors of the AST-C-like system in the mantle suggesting involvement in the immune response. Overall, our study reveals that homologues of the arthropod AST-systems in molluscs are potentially more complex due to the greater number of putative mature peptides and receptor genes. In bivalves they have a broad and varying tissue distribution and abundance, and the elements of the AST-C-like family may have a putative function in the immune response.

## Introduction

Molluscs are the second most diverse animal group after the insects and belong to the speciose Lophotrochozoan clade. Their success is linked to their adaptation to a wide variety of habitats, and they are found from the abysses of the sea to mud flats and even as parasites dwelling in other animals. Unlike the more popular protostome models of the nematodes and insects that have substantial genome rearrangements and gene loss (1–4), the molluscs have a more similar genome organisation and gene repertoire to deuterostomes. Their success, exquisite diversity in form and function and less rearranged genomes makes the molluscs of particular interest for evolutionary studies directed at deciphering the evolution, diversification and role of neuroendocrine factors (1, 5).

The allatostatins (AST) are a diverse group of small neuropeptides that have low sequence conservation but have overlapping functions as insect allatoregulatory peptides (6). Three AST peptide families exist and are named allatostatin A (AST-A), B (AST-B) and C (AST-C). They were first described in insects as inhibitors of the biosynthesis by the corpora allatum (CA) gland, of juvenile hormone (JH), an important regulator of arthropod development and reproduction (7–10). The ASTs are now recognized to be involved in a diversity of other activities and play a key role in arthropod physiology (8, 11) and immunity (12–14). The ASTs are best studied in insects and despite the relatively low gene sequence conservation between the AST families their function and distribution has been conserved. This provides an interesting opportunity to assess if functional constraints have shaped AST evolution in the same way across the protostomes.

The members of each AST family derive from distinct precursor proteins that are assumed to undergo proteolytic cleavage to generate multiple peptides with a similar structure and sequence. The exception is the precursor of AST-C which encodes a single peptide. AST peptides bind and activate members of the rhodopsin G-protein coupled receptor (GPCR) superfamily. AST-A and AST-C activate receptors of the rhodopsin-gamma GPCR cluster while AST-B activates receptors of the rhodopsin-beta GPCR cluster (15–17). Sequence orthologues of ASTs and of their receptors have been identified in other protostomes outside the arthropod phylum, such as the molluscs, the second most diverse protostome phylum after the arthropods. The evolution and function of the AST families in molluscs that lack a CA are at present poorly described (18–27).

AST-A was the first AST to be described and was initially isolated from the cockroach, *Diploptera punctata* (28, 29) and the peptides are characterized by a conserved C-terminal FGL-amide motif. In arthropods a single peptide precursor exists and it is mostly expressed in the nervous system and mid-gut (30, 31) and depending on the species encodes a differing number of peptides from 14 to 13 in Blatoidea (*Periplaneta americana* and *Diploptera punctata*) to 4 to 5 in Diptera (*Drosophila melanogaster* and *Anopheles gambiae*) (31). The regulation of JH biosynthesis by AST-A was demonstrated in cockroaches, termites and crickets and across the insects the most conserved physiological role for this peptide is regulation of food intake, inhibition of insect gut motility and regulation of digestive enzyme metabolism (30, 32–35). Recently we and others revealed that in the blood-feeding mosquitos the AST neuropeptides and GPCRs may regulate blood digestion and reproduction (31, 36). A single AST-A receptor (AST-AR) has been described in arthropods but in Diptera there are two receptor genes, that shared a common evolutionary origin with the vertebrate KISS (KISSR) and galanin (GALR) receptors (15, 31, 37, 38). In molluscs, buccalins are orthologues of insect AST-A, and were first identified and functionally described in the gastropod *Aplysia californica* where they regulate muscle contraction and feeding (18, 24). Currently buccalins have been reported in several molluscs where they are suggested to regulate reproduction and spawning in the Sydney rock oyster (*Saccostrea glomerata*) (39) and in the Mediterranean mussel (*Mytilus galloprovincialis*) their presence in the mantle has been linked to a role in shell formation (37).

The functions of AST-B and AST-C peptides have received much less attention. AST-B peptides are encoded by a precursor that in different species generates a variable number of small peptides. In *D. melanogaster* the AST-B precursor gives rise to 5 peptides but in *Rhodnius prolixus* 12 peptides are generated (40) and they are characterized by the presence of two conserved tryptophan residues and a C-terminal amide (WxW-amide). AST-B peptides were first isolated from the brain-corpora cardiaca-corpora allata-suboesophageal ganglion complex of the migratory locust, *Locusta migratoria* and were shown to inhibit hindgut and oviduct contractions, and thus were initially named myoinhibiting peptides (MIPs) (41). The allatostatic properties of AST-B/MIP peptides were only discovered after their isolation in the two-spotted cricket (*Gryllus bimaculatus*) (9). Subsequently other functions have been identified for AST-B/MIP including regulation of ecdysis in the silkworm (*Bombyx mori*) (42, 43), the circadian clock in Drosophila (*D. melanogaster*) (44), feeding and locomotion in the cockroach (*Leucophaea maderae*) (45), reproduction in the locust (*Locusta migratoria*) (46) and the immune response in the green mud crab (*Scylla paramamosain*) (14). In insects, the AST-B/MIP precursor is most commonly found in the nervous system (45, 47, 48). The functions of AST-B/MIP have also been described in other protostomes such as annelids (where they are known as MIP) and regulate larval settlement and feeding (49). The AST-B/MIP peptides activate the same GPCRs that are activated by insect sex peptide (23, 50, 51) and they are proposed to be proximate with the orphan receptors, GPR142 and GPR139, in vertebrates (15, 16).

AST-C was first isolated from the head of the tobacco hornworm (*Manduca sexta*) (52) and unlike other insect ASTs, the precursor produces a single peptide, although other genes, AST double C and triple C exist in the genomes of some arthropods including insects (53, 54). The AST-C mature peptide has a conserved C-terminal PISCF motif (8, 55, 56). The function of AST-C varies and includes inhibition of muscle contraction, feeding suppression, reduced fecundity and inhibition of ovarian development and regulation of circadian activities (57–62). In *D. melanogaster* AST-C was also recently proposed to regulate nociception and immunity (13). Receptors for AST-C are proposed to have a common origin with the vertebrate Somatostatin receptors (SSR) (15, 63, 64).

The present study revisits and enriches understanding of AST-like peptide and receptor evolution in the molluscs and takes a comparative genomics approach integrating results from previous genome and transcriptome studies (15, 19–21, 26, 65). To elucidate the potential functions of the AST-like families and GPCRs in molluscs we searched bivalve transcriptomes and targeted their potential role in the mantle, a multifunctional tissue well recognized for its contribution to shell production (37, 66–69) and with an emerging role as an innate immune barrier (70, 71). Taking into consideration the emerging role of AST family members in immunity of insects (12–14), the response of specific AST-like family members on the mantle of the Mediterranean mussel (*M. galloprovincialis*) exposed to an immune challenge with bacteria lipopolysaccharide (LPS) and heat inactivated *Vibrio harveyi*, a common pathogenic bacteria of the marine environment, was established (72–74).

## Methods

### Sequence Database Searches

#### Receptors

Searches for the homologues of the insect AST-GPCRs (AST-AR, AST-BR and AST-CR) were performed in molecular data available for nine representative species of the Mollusca phylum using the deduced protein sequences of the previously identified *M galloprovincialis* buccalin receptor [buccalin-R, previous AST-AR (37)] and *D. melanogaster* AST-BR (NP_001284892.1) and AST-CRs (Drostar1, AAG54080.1 and Drostar2, AAL02125.1). Searches were performed against molecular data available in NCBI (https://www.ncbi.nlm.nih.gov) using blastp in the species-specific sub-datasets available for five bivalves: two members of the Mytilidae family, *M. galloprovincialis* (taxid:29158) and *Mytilus coruscus* (taxid:42192), two members of the Ostreidae family, *Crassostrea gigas* (taxid:29159) and *Crassostrea virginica* (taxid:6565) and one member of the Pectinide family, the scallop *Mizuhopecten yessoensis* (taxid:6573); three gastropods, *Aplysia californica* (taxid:6500) of the Aplysidae family, *Biomphalaria glabrata* (taxid:6526) of the Planorbidae family and *Lottia gigantea* (taxid:225164) of the Lottidae family; and also one cephalopod the octopus *Octopus bimaculoides* (taxid:37653).

To increase the number of receptor sequences from the phylum Mollusca, selected genomes of representatives of different orders available from the NCBI Genomes database (https://www.ncbi.nlm.nih.gov/genome/) were also procured using the tblastn algorithm with the *M. galloprovincialis* deduced protein sequences of AST-like receptors as the query (**Supplementary Table 1**). The available genome assemblies of the Polyplacophora class were also searched. All molluscs mentioned above and the general non-redundant protein sequences (nr) database at NCBI were also searched with the *Capitella teleta* GALR-like (ELU13887.1) for other protostome orthologues.

For comparative analysis, sequence searches were also performed in other Lophotrochozoan phyla namely, the brachiopod *Lingula anatina* (taxid:7574), two annelids *Capitella teleta* (taxid:283909) and *Platynereis dumerilii* (taxid:6359) and in three arthropods of the Ecdysozoans namely, the diptera *Anopheles gambiae* (taxid:7165), the coleoptera *Tribolium castaneum* (taxid:7070) and the branchiopod *Daphnia pulex* (taxid:6669). Sequences for the homologue receptors in the vertebrates, human (*Homo sapiens*, taxid:9606), spotted gar (*Lepisosteus oculatus*, taxid:7918) and for the invertebrate deuterostome, the amphioxus (*Branchiostoma floridae*, taxid:7739) were retrieved for comparison with the Lophotrochozoan and Ecdysozoan sequences. Sequence hits with a cut off < e^-30^ were retrieved, and their identity was confirmed by searching against the *D. melanogaster* (taxid:7227) and *H. sapiens* (taxid:9606) databases.

Searches were also performed in mantle transcriptomes available from the Mediterranean mussel (*M galloprovincialis*, SRP 063654) (68) and the hard-shelled mussel (*M. coruscus*, kindly provided by Yifeng Li and Jin-Long Yang, SHOU, China) to identify the corresponding gene transcripts. Putative receptors were retrieved based on their sequence similarity (cut off < e^-30^) with *M. galloprovincialis* and *D. melanogaster* homologues or using transcriptome annotations. All sequences retrieved were translated using the Expasy translate tool (https://web.expasy.org/translate/) and their identity confirmed as described above.

#### Peptide Precursors

Mollusca buccalin precursors (37) and *M. galloprovincialis* and *M. coruscus* orthologues of the insect AST-B/MIP and AST-C peptide precursors were initially obtained by exploring the species-specific mantle transcriptome annotation available “in house”. The deduced protein sequence of MIP-like and AST-C-like were used to identify the corresponding coding genes and additional gene isoforms (**Supplementary Table 1**). The mussel precursor protein sequences were translated with the Expasy translate tool (https://web.expasy.org/translate/) and the localization of the predicted mature peptides was manually deduced by a) the identification of monobasic, dibasic, or tribasic consensus cleavage sites (RR, KR, KK) and b) the identification of the conserved mature peptide motifs such as the two conserved tryptophan (W) residues (W(_X_)W-amide) in AST-B/MIPs and the two conserved cysteine (C) residues in AST-Cs. The mature peptides and the full peptide precursors were procured in other molluscans (bivalves, gastropods cephalopods and the polyplacophoran) using a similar strategy. Homologues from other representatives of the lophotrochozoan clade such as one brachiopod and two annelids were also characterized for comparisons (**Supplementary Figure 1**).

### Sequence Comparisons and Phylogenetic Analysis

Multiple sequence alignments (MSA) of the deduced peptides and receptor protein sequences were performed using the MUSCLE algorithm available in Aliview 1.18 (75). For phylogenetic analysis the receptor alignments were manually inspected, and sequence gaps removed, and the edited alignments were used to construct Bayesian Inference (BI) and Maximum Likelihood (ML) phylogenetic trees. The Buccalin-R/AST-AR and AST-C-like/AST-C (members of the Rhodopsin γ family) trees and the MIP-R/AST-BR trees (members of Rhodopsin β family) included sequences from representatives of 27 molluscs (11 bivalves, 11 gastropods, 4 cephalopods, 1 polyplacophor), 1 brachiopod, 2 annelids, 1 cephalochordate and 2 vertebrates (**Supplementary Table 1**) and were built in the CIPRES Science Gateway v3 using an LG model (selected using model test-ng 0.1.5) since they best fitted the data (76). The BI trees were built in MrBayes (77) run on XSEDE v3.2.7a with 1.000.000 generation sampling and probability values to support tree branching. The ML trees were built with the RAxML v8.2.12 (78) method with 100 bootstrap replicates. The Buccalin-R/AST-AR and AST-CR-like/AST-CR trees were mid-rooted according to previous models proposed for receptor sequence evolution (15), and MIP-R/AST-BR were rooted with the *H. sapiens* (NP_000721) and *L. oculeatus* (XP_006629714) cholecystokinin receptor type A (CCKAR) branch. To build the phylogenetic trees for the AST-Rhodopsin γ family GPCRs the sequences of the metazoan KISSRs and GALRs were also included as they are suggested to have evolved from the same ancestral gene as protostome Buccalin-R/AST-AR [sequences obtained from (37)] and also from database searches using the predicted receptor proteins of *M. galloprovincialis* as queries. For the trees of the AST-Rhodopsin β family GPCRs the related protostome receptors from the FMRFamide (FMRFaR), RGWamide (RGWaR), Proctolin (ProctR) and Myosupressin/Myomodulin (MyoR) families were also included as well as the vertebrate sequence orthologues GPR142 and GPR139 from *H. sapiens* (GPR142, NP_001318005.1 and GPR139, NP_001002911.1) and *L. oculeatus* (GPR142, XP_006635495 and GPC139, XP_006637109) (15, 16, 79).

Receptor sequence alignments and percentage of sequence identity was displayed and calculated in the GenDoc programme (http://www.nrbsc.org/gfx/genedoc/). The mature peptide alignments were established using Clustal W (https://www.genome.jp/tools-bin/clustalw). Receptor structures were predicted in Protter (http://wlab.ethz.ch/protter) using the default settings and the outputs annotated in Inkscape to highlight the conserved aa positions across species. The receptor signal peptide was predicted using the SignalP-5.0 Server (80) and the DeepLoc1 server was used to explore protein cellular localization (81).

### Neighbouring Gene Analysis (Short Range Synteny)

The neighbouring gene environments of the annelid *C. teleta GALR-like* and *Buccalin-R* that map to SuperContig CAPTEscaffold_148 (scaffold_148) and SuperContig CAPTEscaffold_45 (scaffold_45), respectively and *H. sapiens* GALRs and *D. melanogaster AST-ARs* (*DAR1* and *DAR2*) were characterized to infer potential ancestral origin and to support the identification of the new protostome sister clade of the deuterostome GALRs. Ten genes upstream and downstream of the *C. teleta* gene loci were collected and they were used to search for gene homologues in the *H. sapiens GALR1* (chromosome 18), *GALR2* (chromosome 17), *GALR3* (chromosome 22) and KISSR (chromosome 19) and in *D. melanogaster DAR1* (chromosome X) and *DAR2* (chromosome 3R) genome regions. The BioMart tool available from Ensembl was used to compare the *C. teleta* and *D. melanogaster* genome regions. The deduced *C. teleta buccalin-R* and *GALR-like* neighbouring protein sequences were searched against the species-specific *H. sapiens* (taxid:9606) and *D. melanogaster* (taxid:7227) datasets available from NCBI (https://www.ncbi.nlm.nih.gov). The gene loci of the top protein hits (with the lowest e value) were retrieved based on the genome assemblies available from NCBI (*D. melanogaste*r, Release 6.32, and *H. sapiens*, GRCh38.p13). The *O. bimaculoides GALR-like* genome region on SuperContig KQ419625 was also analysed and the five neighbouring genes were retrieved and searched against *C. teleta* and *H. sapiens* genomes.

### Animal Manipulation and Sampling

Mediterranean mussels (*M. galloprovincialis*) were obtained from a local producer in the Ria Formosa (Olhão, Portugal). For the experimental immune challenge mussels (length 4.35 ± 0.34 cm, soft body dry mass 1.61 ± 0.46 g) were transported live to the Centre of Marine Sciences (CCMAR) where they were cleaned and acclimatized for a week in 5 litres of aerated seawater (SW) prior to the immune challenge at 20 - 22°C. Animals were fed daily with a mixture of a commercial dried microalgae diet (PHYTOBLOOM, Necton, Portugal). For tissue sampling animals were opened by cutting the adductor muscle and the mantle edge from the region most distal to the umbo (referred to as the posterior region) was dissected out and snap frozen in dry ice and stored at -80°C for RNA extraction. For tissue distribution, cDNA samples (n = 3 for each tissue) from gills, digestive gland, mantle edge and haemolymph available in the lab were used.

### Immune Challenge

Mussels were exposed to heat-inactivated *V. harveyi* by introducing them into the bathing seawater. The *V. harveyi* (kindly donated Dr M. Manchado, IFAPA, Puerto Santa Maria, Spain) was grown in TSB medium supplemented with 1% NaCl and the number of cfu/ml was determined on TSA/1% NaCl agar plates. For the bacterial challenge 5 x 10^7^ cfu/ml of the heat inactivated bacteria suspended in 1 L of sterile seawater was used. The *V. harveyi* bacteria was heat inactivated by boiling the culture for 2 hours.

For the challenge experiments mussels (n = 80) were randomly distributed in triplicate tanks. The experiments were performed in 2 L plastic tanks containing 1 L of filtered seawater (0.45 μm) that was collected from their natural environment. Each of the control (3 tanks) and exposed (3 tanks) tanks contained 12 - 13 individuals (total 40 animals per group). The seawater in the experimental tanks was constantly aerated with aquarium air-pumps and the temperature was 20 - 22°C, pH was 8.1 ± 0.1 and salinity 37 ppt and the experiments were conducted under natural photoperiod for March 2021 in the Algarve. Control mussels were maintained in seawater and transferred after 15 h to new tanks and the immune challenged mussels were exposed for 15 h to heat-inactivated *V. harveyi* (5 x 10^7^ cfu/litre) and then transferred to new tanks containing clean filtered seawater. Specimens (n = 6 per timepoint) from control and challenged tanks were sampled (as outlined above) at 0, 6, 12, 24 and 36h post exposure. Animals were not fed during the experiment and no mortality was observed.

### RNA Extraction and cDNA Synthesis

Total RNA (tRNA) from control and immune challenged mantle edge was extracted using an E.Z.N.A kit (VWR, USA) and DNase treatment was performed after elution using a Precision DNase kit (Primer design, UK) according to the manufacturers protocol. For extraction, collected tissues were defrosted in the lysis buffer and homogenized by mechanical disruption with two iron beads (5 mm) using a Tissue lyser II Qiagen and 1 cycle of 3 min at room temperature.

For cDNA synthesis, DNase treated tRNA (500 ng) was denatured at 65°C for 5 min and quenched for 5 min on ice. Reactions were carried out for a 20 μl final volume with 10 ng of pd(N)6 random hexamers (Jena Bioscience, Germany), 2 mM dNTPs (ThermoScientific, USA), 100 U of RevertAid Reverse Transcriptase and 8 U Ribolock RNAse inhibitor (ThermoScientific). Reaction conditions were: 10 min, 20 °C; 60 min, 42 °C; 5 min , 70 °C. The quality of the cDNA produced was assessed by amplification of mussel ribosomal subunit *18S* rRNA using specific primers (**Table 1**) and the following thermocycle: 95°C, 3 min; 25 cycles (95°C, 20 sec; 60°C, 20 sec; 72°C, 20 sec); 72°C, 5 min.

**Table 1:** List of primers used in qRT-PCR analysis for the bivalve *M. galloprovincialis*.

Transcript		Sequence (5’ ➔3´)	T (°C)	Efficiency	R^2^
AST-C-like	Fwd	GCAGTTTCAAGAGCAGGAAGCCT	66°C	95.6%	0.996
	Rev	GGCATTGCACATGGCTTCGTTT			
AST-CR-like VDI53419.1	Fwd	AAACATCGGAAGAGAGGCT	60°C	92.9%	0.999
	Rev	GCATTTCCAATCAGACCGGC			
AST-CR-like VDI15122.1	Fwd	TACGGACGAATTCGAAAACGG	62°C	96.2%	0.995
	Rev	ATTACCAACCAATCCACCGAC			
AST-CR-like VDI08560.1	Fwd	AACACATCCAGTGCTGTCGC	na	na	na
	Rev	CGCCTTATTGAATGCCATACC			
AST-CR-like VDI13242.1	Fwd	CGTCATTCTGCGTTCATCCA	na	na	na
	Rev	CCAAAAAGCCAGAATCGCAG			
TLRa	Fwd	TCATACCTGGGGCCTGCATA	62°C	99.7%	1
	Rev	GTGGCGTCGGTGTTTCAATG			
LYG1	Fwd	TGCAGTGTGATGTCCGAGTC	62°C	95.3%	0.996
	Rev	GTATGCTGCCACTCCACCTT			
C3-like	Fwd	CCAGCACCAAACTGTCCACT	62°C	100.3%	0.995
	Rev	ACGATTCGTCCCGTCTCATC			
18S	Fwd	GTGCTAGGGATTGGGGCTTG	60°C	95.5%	0.999
	Rev	TAGTAACGACGGGCGGTGTG			
EF1 α	Fwd	GAAGGCTGAGGGTGAACGTG	60°C	96.2%	0.996
	Rev	TCCTGGGGCATCAATAATGG			

na, not amplified.

### Tissue Expression Analysis

To characterise the tissue distribution publicly available control tissue transcriptomes of four bivalves: *M. galloprovincialis*, *M. coruscus*, *C. gigas* and *M. yessoensis* were retrieved and analysed. Public transcriptome data available for control tissues including the gills, muscle, mantle, digestive gland/hepatopancreas, haemocytes and nerve ganglia were searched using the species-specific nucleotide sequences identified for the peptide precursors and receptors. Searches were carried out using Blastn and the corresponding sequence read archive (SRA, https://www.ncbi.nlm.nih.gov/sra/) for each of the species analysed (**Supplementary Table 2**). Maximum target sequences were adjusted to 1000 and sequence hits with > 98% nucleotide identity were selected. FPKM counts were calculated taking into consideration the number of reads, gene length and the transcriptome sequencing depth.

The involvement of the bivalve homologues of the arthropod ASTs and receptors in the immune response was initially assessed using mantle edge transcriptomes of *M. galloprovincialis* challenged with Lipopolysaccharide (LPS, *E. coli* LPS 0111:B4, Sigma-Aldrich, USA) a major component of the outer membrane of Gram-negative bacteria. Candidate transcripts were identified by exploring available in-house DEG data (p-adj < 0.05, log2-fold > 2) for the mantle edge transcriptomes of control *M. galloprovincialis* (injected with 1x PBS) and LPS exposed *M. galloprovincialis* (injected with 50 μl of 0.5 mg/ml of bacterial LPS in the adductor mussel) from samples collected in the context of another study.

To further explore the involvement of the AST-C-like system in the bivalve response to pathogenic marine bacteria, expression analysis of the *M. galloprovincialis* members was assessed using cDNA (n = 3) from normal tissues (gills, digestive gland, mantle edge and haemocytes) and from the mantle edge of control and exposed specimens to heat-inactivated pathogen, *V. harveyi*, at 0 (n = 6), 6 (n = 6), 12 (n = 6), 24 (n = 6) and 36 h (n = 6) post exposure. Specific primers for the *M. galloprovincialis* AST-C-like peptide precursors and for four AST-CR-like were designed and the PCR products were sequenced, and their identity confirmed (**Table 1**). It was not possible to design specific primers for the *AST-CR-like* VDI60978.1 as its sequence was smaller and the predicted nucleotide coding region was highly identical to other AST-CR-like (97%). The activation of the immune response in *M. galloprovincialis* was confirmed by determining the expression of three humoral factors that have previously been shown in bivalves to respond to *Vibrio* spp., Toll-receptor *TLRa* (82), Lysozyme goose-type 1 *LYG1* (83) and complement-factor *C3-like* (Peng et al., submitted) in cDNA of the mantle edge of control (n = 3) and exposed (n = 3) specimens from 0, 6 and 12 h post exposure (**Table 1**).

Changes in gene expression were assessed by quantitative real-time PCR (qRT-PCR) using SsoFast EvaGreen Supermix (Bio-Rad, Portugal) in a 10 µl final reaction volume containing 200 nM of forward and reverse gene specific primers (**Table 1**) and 2 µl of template cDNA (diluted 1:2). Elongation factor 1-alpha (*EF1α*) and 18s ribosomal subunit (*18S*) were used as reference genes (cDNA diluted 1:50 and 1:500, respectively). QRT-PCR analysis was performed in duplicate reactions (< 5% variation between replicates) using a CFX Connect™ Real-Time PCR Detection System for 96-well microplates (Bio-Rad). Cycling conditions were 95°C, 30 sec; 44 cycles of 95°C, 5 sec; the most appropriate primer annealing temperature, 10 sec (**Table 1**). Melting curves were performed to detect the presence of non-specific products and primer dimers. Reverse transcriptase (RT-) and PCR control reactions were included in each PCR plate to confirm the absence of genomic or PCR contamination. QRT-PCR efficiencies and R^2^ (coefficient of determination) were established (**Table 1**), and data was normalized using the geometric mean of the expression levels of the reference genes.

### Statistical Analysis

Results are presented as the mean ± SEM. Statistical differences were detected using One-Way ANOVA for the tissue distribution and for gene expression between the control and immune challenged mussels with Two-Way ANOVA using a Sidak’s multiple comparison test. Analysis was executed using GraphPad Prism version 8.0 for Mac OS X (USA, www.graphpad.com).

## Results

### Nomenclature

The AST neuropeptides were named due to their inhibitory (allatostatic) actions on JH biosynthesis from the insects CA gland. In molluscs no equivalent organ has been described and JH is specific to insects. In molluscs the sequence orthologues of the arthropod AST-A are known as buccalin and in annelids the orthologues of the arthropod AST-B/MIP are known as MIP. For the lophotrochozoan AST-Cs no nomenclature has yet been proposed. Throughout the manuscript we use the existing lophotrochozoan nomenclature and have named AST-C as AST-C-like (**Table 2**).

**Table 2:** Nomenclature adopted for the molluscan neuropeptide homologues of the arthropod AST peptides and receptors.

	Arthropoda		Mollusca	
	*Peptide*	*Receptor*	*Peptide*	*Receptor*
Allatostatin A	AST-A	AST-AR	Buccalin	Buccalin-R
Allatostatin B/myoinhibitory peptide	AST-B/MIP	AST-B/MIP-R	MIP	MIP-R
Allatostatin C	AST-C	AST-CR	AST-C-like	AST-CR-like

### Orthologues of the Arthropod AST Precursors and Receptors in the Molluscs

**Figure 1:**
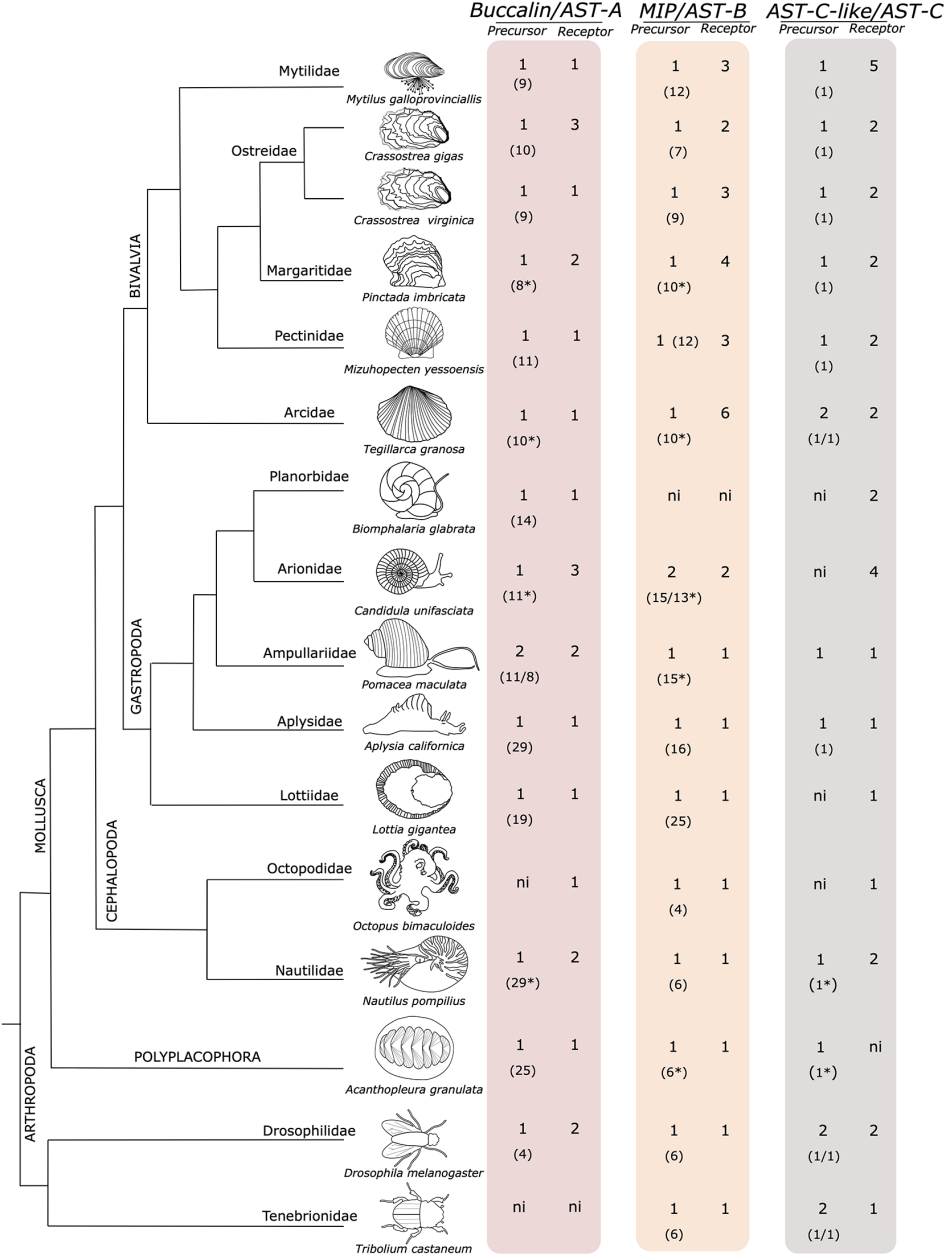
The molluscan orthologues of the arthropod AST precursors and receptors. The number of peptide precursors and receptors retrieved are indicated. The predicted number of deduced mature peptides produced by each peptide precursor is represented within brackets “()”. The dendrogram represents the evolutionary relationship of the species and was manually designed taking into consideration the studies of (84–87). The insect *D. melanogaster* and *T. castaneum* are also represented for comparison. The figure is not drawn to scale. ni- not identified. Complete peptide precursor sequences are available from **Supplementary Figure 1**. *deduced from an incomplete precursor sequence.

Sequence homologues of the three arthropod AST peptide precursors and receptors were found in the 27 mollusc species included in the study and the number of peptide precursor genes and predicted mature peptides and putative receptor genes varied across the species (**Figure 1** and **Supplementary Table 1**, **Supplementary Figures 1A–C**). In other lophotrochozoan phyla the peptide precursors shared a similar organization to the molluscs (**Supplementary Figure 1A**).

#### Buccalin Precursors and Buccalin Receptors

The Mollusca buccalin precursors are the orthologues of the arthropod AST-As and a single precursor gene was found in most molluscs and encoded for multiple mature peptides (**Figure 1** and **Supplementary Figure 1A**). The exception was the gastropods *Conus ventricosus* (CM031604.1 and CM031615.1) and *Pomacea maculata* (XP_025108540.1 and XP_025087986.1) where two putative genes were found. The number of buccalin-R was more variable and while most species possessed a single buccalin-R gene, other species genomes contained two receptor genes (the bivalves *Pinctada imbricata*, *Margaritifera margaritifera*; the gastropods *Alviniconcha marisindica*, *Haliotis laevigata*, *Pomacea maculata* and the cephalopod *Nautilus pompilius* but the bivalve *C. gigas* and the gastropod *Candidula unifasciata* contained three buccalin-R genes. The majority of the mollusca duplicate buccalin-R genes were localized within the same genome region and thus are likely to be tandem gene duplicates (**Supplementary Table 1**).

The buccalin precursors encoded for a different number of peptides. In the Mytilidae family 7 mature peptides were predicted in *M. coruscus* and 9 in *M. galloprovincialis*. In the Ostreidae family, the buccalin precursors have the potential to generate 10 mature peptides in *C. gigas* and *M. hongkongensis* and 9 in *C. virginica* and in the Pectinidae, *P. yessoensis*, 11 mature peptides were predicted (**Supplementary Figure 1A**). In other bivalves, such as the *Margaritifera margaritífera* (Margaritiferidae family), *Pinctada imbricata* (Pteriidae family) and in *Tegillarca granosa* (Arcidae family) at least 8, 8 and 10 putative mature peptides respectively, were encoded, and the full-length precursors still remain to be isolated. No buccalin precursor genes were retrieved from the bivalves *Sinonovacula constricta* and *Ruditapes philippinarum* genomes.

The buccalin precursor in the gastropod *A. californica* (Aplysiidae family) contained the largest number of predicted peptides (29 mature peptides) and the *Gigantopelta aegis* (Peltospiridae family) precursor was the second most rich. In the cephalopod *O. bimaculoides* and most cephalopod genomes explored our searches failed to identify the buccalin precursor except in the *Nautilus pompilius* (Nautilidae family) where 29 peptides were predicted (**Supplementary Figure 1A**). In the polyplacophore *Acanthopleura gran*ulata a single buccalin precursor was found (**Supplementary Figure 1A**).

Previously, a putative buccalin-like precursor with a similar organization to the buccalin precursor was identified in molluscs, which generates a series of highly identical V- amide mature peptides with a conserved PFDRLASGLV/I-amide sequence (37) (**Supplementary Figure 2**). These buccalin-like peptides were recently reclassified and proposed as a new Mollusca neuropeptide family, the LASGLI/V-amide peptide family (19, 25). For this reason, the buccalin-like peptides (LASGLI/V-amide peptide) were excluded from the present analysis.

#### MIP Precursors and MIP Receptors

In Mollusca a single MIP-B peptide precursor encoding multiple peptides was found (**Supplementary Figure 1B**) in most species, the exception was the gastropod, *C. unifasciata*, where two precursors were retrieved. The number of MIP-Rs was variable (**Figure 1**). A single receptor gene was found in the gastropods, cephalopods and in the polyplacophore genomes analysed but gene number was very variable in bivalves where multiple receptors were found with 3 in *M. galloprovincialis* and 6 in *T. granosa* (**Supplementary Table 1**). No MIP or MIP-R was found in the gastropod *B. glabrata*. In bivalves, the number of mature MIP peptides varied from 11 in the Mytilidae to 12 in the Pectinidae (**Figure 1** and **Supplementary Figure 1B**). The gastropod peptide precursors encoded the greatest number of peptides, and 25 MIP mature peptides were predicted in *L. gigantea* and 16 in *A. californica*. In other gastropods the minimum number of mature peptides found was 8 but most precursors were deduced from species genomes and were incomplete and it seems likely that more peptides may be produced. In the cephalopod *O. bimaculoides* the MIP precursor encoded the least number of peptides (only 4) but in other taxa at least 6 peptides existed (**Supplementary Figure 1B**). In the polyplacophore a single MIP precursor was found, which encoded at least 6 mature peptides.

#### AST-C-Like and AST-C-Like Receptors

In Mollusca a single AST-C-like peptide precursor that encodes a single mature peptide was found in all the species analysed (**Figure 1** and **Supplementary Figure 1C**). The only exception was the bivalve *T. granosa* where two identical mature peptides precursor genes localized in the same genomic fragment (JABXWC010000007.1) were found. In contrast, in other molluscs receptor number was variable and 5 putative *AST-CR-likes* were retrieved from bivalves of the Mytilidae family and four were found in the gastropod *C. unifasciata* (Arionidae family). The other representatives of the diverse Mollusca classes possessed 1 to 2 AST-CR-like (**Figure 1**). Genes encoding the *AST-C-like* peptide precursors were not predicted in available protein coding gene data for *M. galloprovincialis* and *M. coruscus* but searches in mantle transcriptomes identified a single *AST-C-like* gene transcript in *M. coruscus* which mapped with 100% identity to the genome (CM029607.1). An orthologue sequence was identified in the genome of *M. galloprovincialis* (UYJE01007806.1) when the *M. coruscus* AST-C-like sequence was used for searches.

### Phylogeny of the Mollusca Receptors

**Figure 2:**
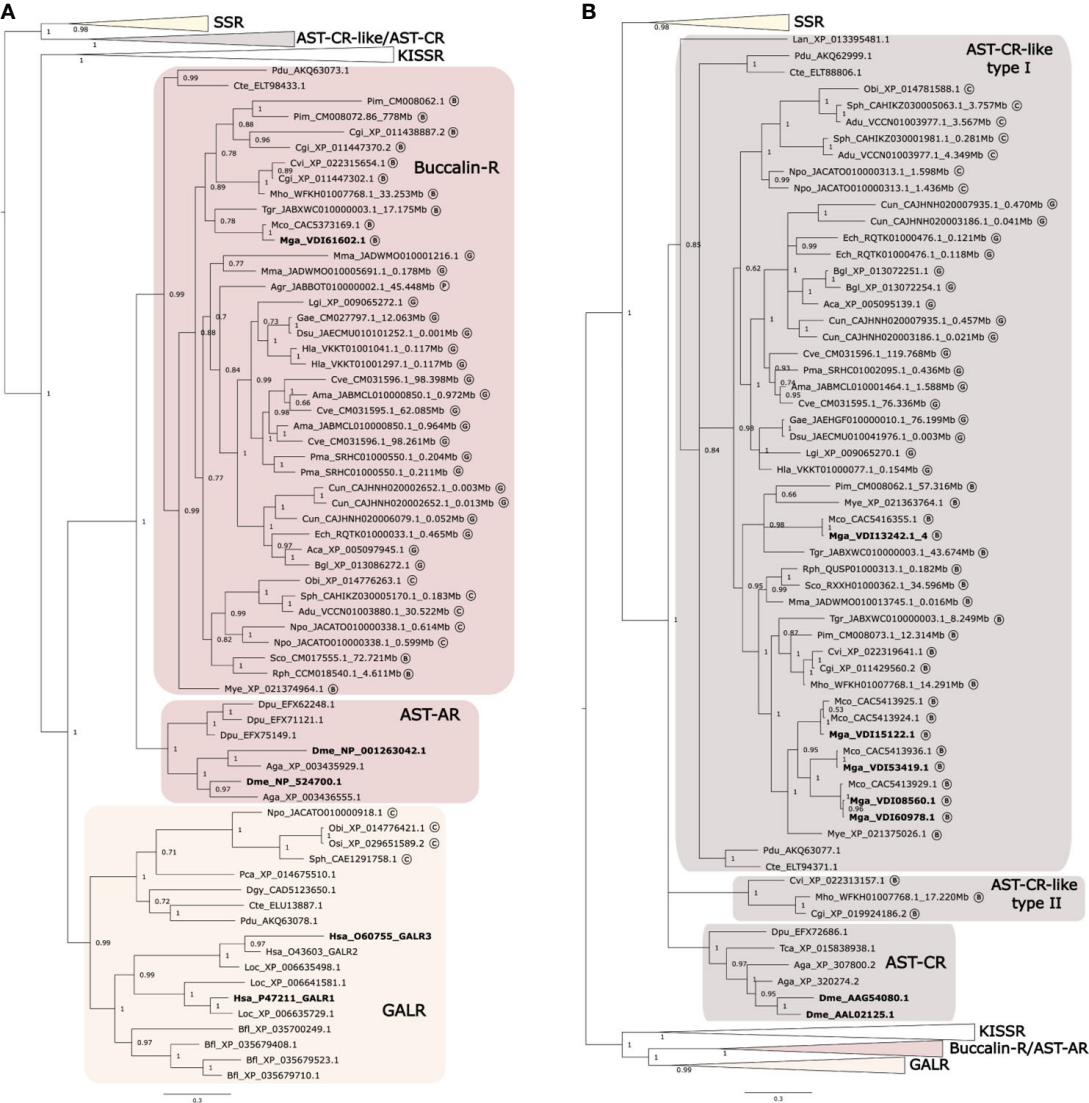
Phylogenetic trees of the Molluscan Buccalin and AST-C-like receptors and orthologues from other lophotrochozoans, ecdysozoans and deuterostomes. Two subsets of the same phylogenetic tree showing the expansion of the different metazoan receptor family members **(A)** Buccalin-R/AST-AR and **(B)** AST-C-like/AST-C are represented to facilitate interpretation. The tree was built using the Bayesian inference (BI) method with 1,000,000 generations and posterior probability values and was constructed using an LG model. A tree with a similar topology was also obtained using the Maximum likelihood (ML) method (**Supplementary Figure 3**). The AST-AR and AST-CR tree was mid-rooted taking into consideration the clustering of the sequences. Circled letters indicate: B, bivalves; G, gastropods, C, cephalopods; P, polyplacophore species used in the analyses. The sequences that were retrieved from non-annotated genomes have their putative localization (Mbp) indicated.

**Figure 3:**
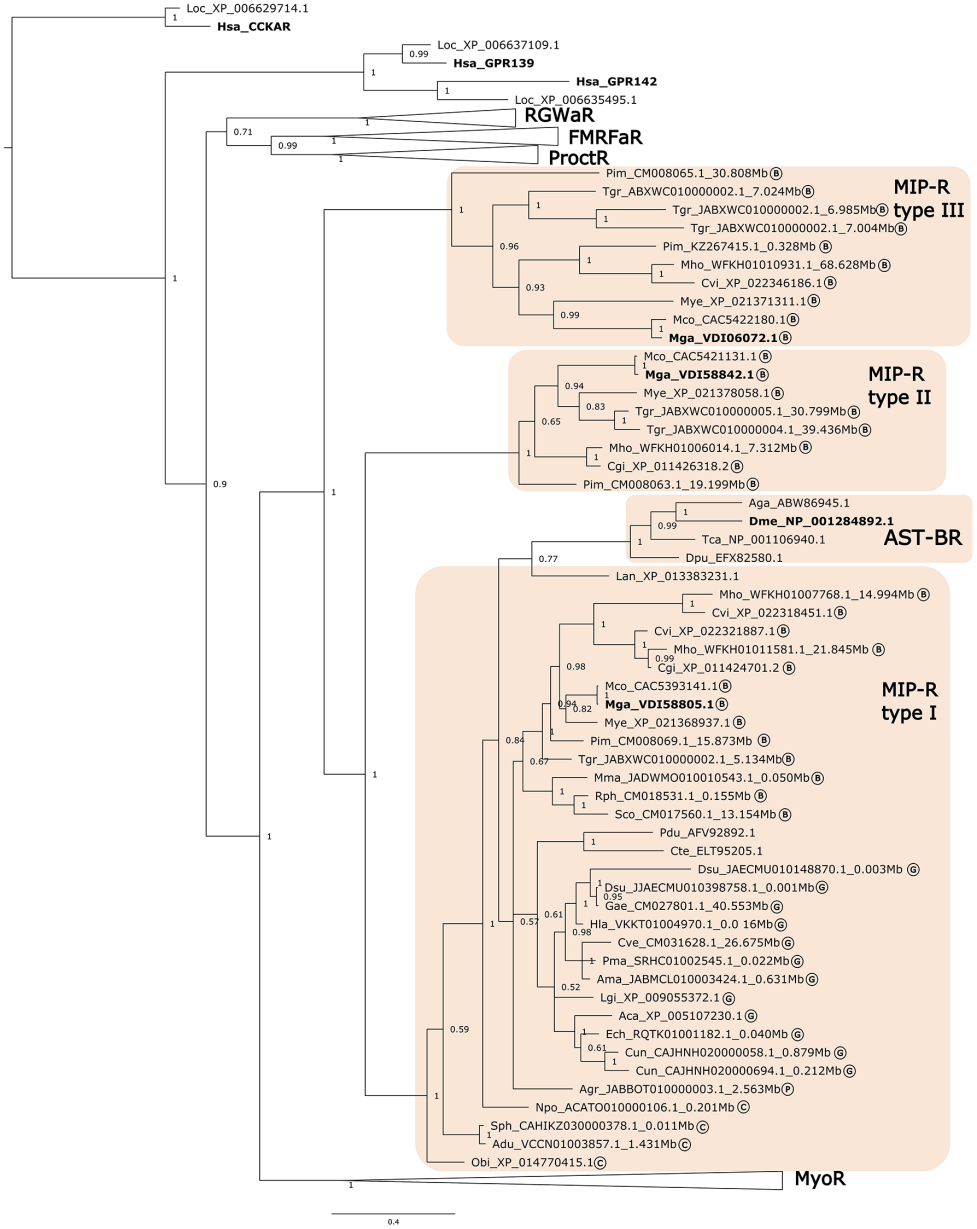
Phylogenetic tree of the Molluscan MIP receptors and orthologues from other lophotrochozoans, ecdysozoans and deuterostomes. The tree was built using the Bayesian inference (BI) method with 1,000,000 generations and posterior probability values and was constructed with an LG model. A tree with a similar topology was also obtained using the Maximum likelihood (ML) method (**Supplementary Figure 3**). The receptor clusters of FMRFamide (FMRFaR), RGWamide (RGWaR), Proctolin (ProctR) and Myosupressin/Myomodulin (MyoR) were collapsed. The tree was rooted with the *H. sapiens* (NP_000721) and *L. oculeatus* (XP_006629714) cholecystokinin receptor type A (CCKAR). Circled letters indicate: B- bivalves; G-gastropods, C- cephalopods; P- polyplacophore species used in the analysis. The sequences that were retrieved from non-annotated genomes have their putative localization (Mbp) indicated.

The retrieved receptors from Mollusca were compared with the orthologue receptors in other lophotrochozoans, arthropods and deuterostomes by building BI phylogenetic trees (**Figures 2A, B** and **Figure 3**). The ML tree had a similar branch topology (**Supplementary Figures 3A, B**). The protostome Buccalin-R/AST-AR and AST-CR-like/AST-CR family members belonged to the GPCR-rhodopsin γ subfamily. The protostome MIP-R/AST-BR family members belonged to the GPCR-rhodopsin β subfamily. All the Mollusca sequences within each receptor family clustered together based on their sequence homology in the phylogenetic trees and the lophotrochozoan receptors formed sister branches with the arthropod receptor homologues. The receptors of the Mytilidae family, *M. galloprovincialis* and *M. coruscus*, always clustered in proximity and the same was observed for the Ostreidae family, *C. gigas* and *C. virginica*. Clustering of the Mollusca receptors revealed that the variable number of members found within each family resulted from lineage and species-specific duplication events.

#### Buccalin-R

The topology of the phylogenetic tree confirmed that no Buccalin-R/AST-ARs existed in deuterostomes and that Mollusca as well as other protostome receptors shared a common origin with the metazoan KISSR and GALR (15, 31, 37, 88, 89) (**Figure 2A**). Our searches also confirmed that homologues of the deuterostome KISSRs exist in bivalves and gastropods and that GALR-like sequences were only found in cephalopods. The cephalopod GALR-like sequences clustered with the annelid GALR-like from *C. teleta* (ELU13887) and *P. dumelii* (AKQ63078) and with the deuterostome GALRs (**Figure 2A**) irrespective of the phylogenetic tree building method. This reveals for the first time the presence in Mollusca of a GALR-like clade, which is apparently absent from the bivalves, gastropods and polyplacophore.

#### MIP-R

Homologues of the arthropod AST-BRs (MIP-Rs) were identified in molluscs and other lophotrochozoans and the clustering of the retrieved bivalve sequences suggested that there were three MIP-R subtypes (**Figure 3**). Type I MIP-Rs was assigned to the Mollusca receptors that were present in most species and that clustered with the other lophotrochozoans and in the same branch as the arthropod AST-BRs. The other two MIP-R clusters were named type II and type III and contained sequences from bivalves (**Figure 3**). Identification of 3 MIP-R subtypes in bivalves and their absence from other protostomes suggests that they emerged prior to the divergence of the ecdysozoan and lophotrochozoan lineages (**Figure 3**). The protostome RGWaR, FMRFaR, ProctR and MyoR that are proposed to have radiated from the same common ancestor as MIP-R/AST-BRs were included in phylogenetic tree (15, 16, 79) and the tree topology confirmed that all receptors shared a common origin with the orphan deuterostome GPR139/142 (15, 16).

#### AST-CR-Like

The arrangement of the sequences within the Mollusca AST-CR-like branch confirmed that the receptors shared a common origin with vertebrate SSRs (**Figure 2B**). A large gene family expansion occurred within the Mytilidae family and originated four of the five receptor isoforms retrieved from *M. galloprovincialis* and *M. coruscus*. In addition, the clustering of one of the Ostreidae duplicate receptors from *C. gigas*, *C. virginica* and *M. hongkongensis* in distinct branches from the other lophotrochozoan receptors suggested that the two gene copies were under different evolutionary pressure (**Figure 2B**). Genome mapping revealed that the duplicate Ostreidae receptor genes mapped in proximity in the same genome fragment and are likely to be the result of a tandem gene duplication (**Supplementary Table 1**).

### Buccalin, MIP and AST-C-Like Mature Peptides in Molluscs

#### Buccalin Mature Peptides

**Figure 4:**
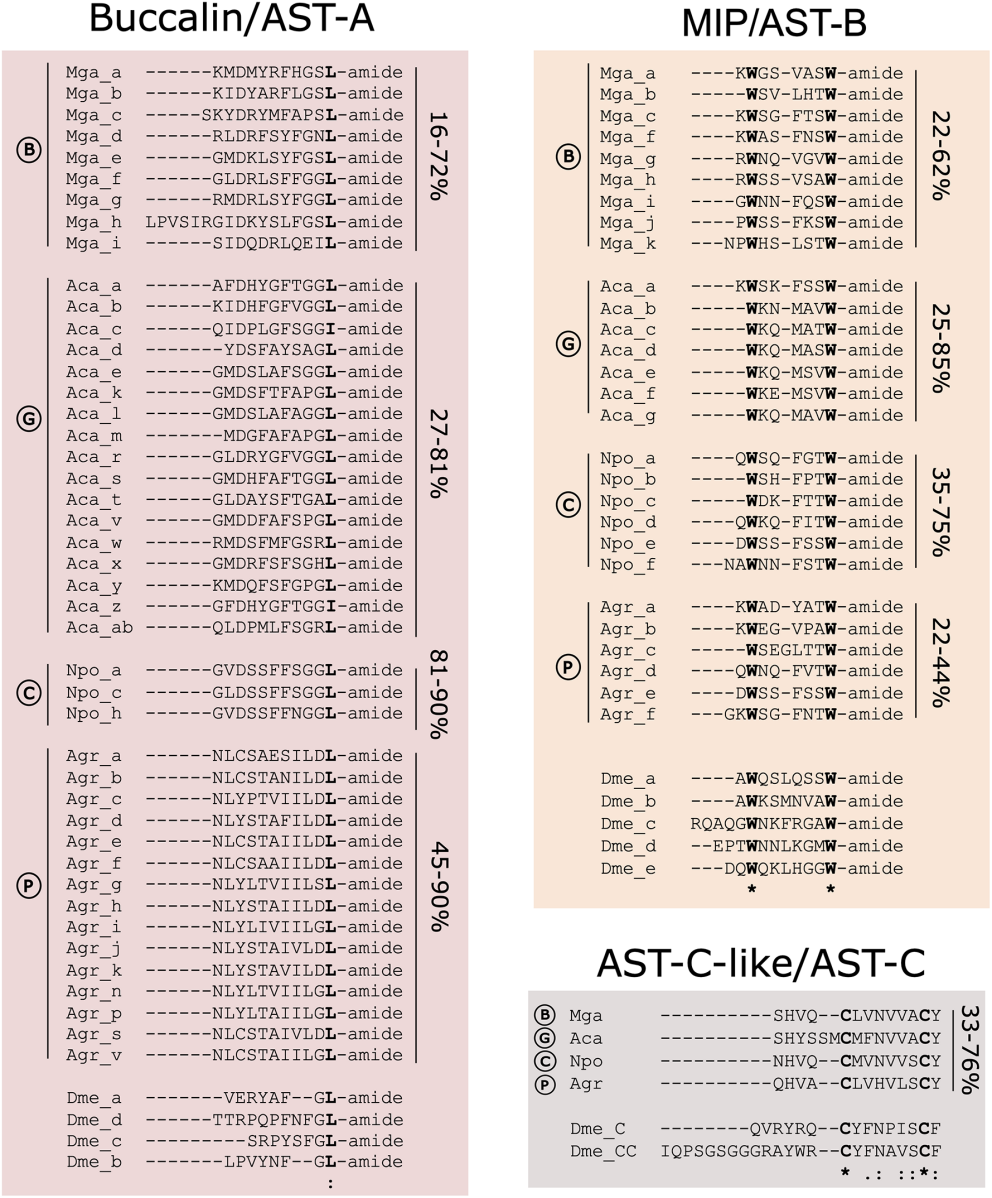
Molluscan Buccalin, MIP and AST-C-like mature peptide sequences. Identical peptides arising from the same peptide precursor are not represented. The deduced peptides were named with a letter (a to z) based on their position within the peptide precursor. The percentage of amino acid sequence identity of the mature peptides encoded by the same precursor in each species is indicated at the side for each peptide group. The bivalve (B) *M. galloprovincialis* (Mga), gastropod (G) *A. californica* (Aca), cephalopod (C) *N. pompilius* (Npo) and polyplacophore (P) *A*. *granulata* (Agr) mature peptides and the *D. melanogaster* (Dme) peptides are represented for comparison. Totally conserved amino acids are marked in bold. “*” indicates total amino acid conservation, “:” indicates a site belonging to groups exhibiting strong similarity and “.” indicates a site belonging to a group exhibiting weak similarity. Full-length peptide precursor sequences are available in **Supplementary Figure 1**.

In molluscs the buccalin peptide precursors encoded L-amide peptides (as found in *A. californica* (18) and a different number of peptides (**Supplementary Figure 1A**). The gastropod and the cephalopod *N. pompilius* precursors encoded the largest number of mature peptides (**Figure 1** and **Supplementary Figure 1**). The bivalve and cephalopod buccalin precursors encoded peptides with a conserved C-terminal L-amide (like *D. melanogaster* AST-A) (**Figure 4**) but in gastropods it also encoded I-amide peptides and all predicted peptides were less than 55% identical in sequence to the *D. melanogaster* AST-A. The number of predicted peptides in the buccalin precursor varied across the molluscs. In bivalves, the buccalin precursor in the representatives of the Mytilidae family, encoded 9 and 7 mature L-amide peptides, respectively in *M. galloprovincialis* and *M. coruscus* that all had different sequences (**Figure 4** and **Supplementary Figure 1A**).

In gastropods, the *A. californica* buccalin precursor encoded the largest number of predicted peptides (29 mature peptides) and 25 were L-amine and the remaining 3 were C-terminal I-amide and produced 17 different peptides (**Figure 4**). Other gastropod precursors only encoded L-amide peptides (*G. aegis, A. marisindica*, *C. ventricosus*, *D. subfuscus*, *P. maculata*, *H. laevigata*, *E. chlorotica*) but the *B. glabrata* precursor only encoded I-amide peptides (11 in total) (**Supplementary Figure 1A**). In *O. bimaculoides* and other cephalopod genomes searches failed to identify the buccalin precursor except in *N. pompilius* and 29 L-amide peptides were predicted, and most of them were identical in sequence and only 3 different types of mature peptides were produced (**Figure 4** and **Supplementary Figure 1A**). The buccalin precursor in the polyplacophore *A. granulata* encoded 25 L-amide peptides with 15 different sequences (**Figure 4** and **Supplementary Figure 1A**). Conservation of the mollusc mature peptides encoded within the precursor was distinct and the amino acid sequence identity of the *M. galloprovincialis* mature peptides varied from 16 - 72% and the predicted Mga_c and Mga_i peptides were most divergent and Mga_g was the most conserved (72% aa identical) with Mga_d, e and f.

#### MIP Mature Peptides

The MIP precursors in common with the mollusc buccalin precursor have the potential to generate multiple peptides by proteolysis of a single polypeptide precursors and the highest peptide numbers were identified in the gastropods (**Figures 1**, **4** and **Supplementary Figure 1B**). For MIP a single peptide precursor was found and in the bivalve Mytilidae the number of mature peptides varied from 11 in *M. coruscus* to 12 in *M. galloprovincialis* but in the gastropod *C. unifasciata* two MIP precursors existed and they encoded for different peptides (**Figure 1** and **Supplementary Figure 1B**). The mollusca MIP mature peptides varied from 7-9 aa in length and the sequence identity in the *M. galloprovincialis* precursor revealed that 3 peptides (Mga_c, d and e) were identical and the peptides overall, shared 22 to 62% aa identity (**Figure 4**). The MIP precursor in *A. californica* produced 16 peptides of which 7 had different sequences and overall, they shared 25-85% aa identity. The gastropod *L. gigantea* MIP precursor was the most peptide rich (25 MIPs in total) and it encoded putative mature peptides of different sizes, and the majority were slightly longer (12 aa) than most molluscan peptides due to their extended N-terminal region (**Supplementary Figure 1B**). The same occurred for the cephalopod *S. pharaonic* and *A. dux* MIPs but *N. pompilius* had peptides of a similar length to other mollusca MIPs and all the encoded peptides (6) differed and the same was true for the polyplacophore *A. granulata* (**Figure 4** and **Supplementary Figure 1**). All the mollusc mature peptides possessed two conserved tryptophan (W) residues separated by 4-7 aa residues (W(x_4-7_)W, where x represents any aa). In *D. melanogaster* there were five AST-B/MIP peptides that contained two conserved W residues separated by 6 aa (W(x_6_)W). The 12 MIP mature peptides in the bivalve *M. galloprovincialis* shared 38 to 78% aa sequence identity with the *D. melanogaster* orthologues.

#### AST-C-Like Mature Peptides

A single AST-C-like peptide was encoded by the AST-C precursor and in the molluscs analysed, the peptide was 13-15 aa in length (**Figure 4** and **Supplementary Figure 1C**). AST-C in molluscs like AST-C and AST-CC of *D. melanogaster* possessed two conserved cysteine (C) residues that form a disulphide bond in the peptide (**Figure 4**). Within molluscs the aa conservation varied from 33 to 76% and the mollusc AST-C-like mature peptide shared higher sequence conservation with *D. melanogaster* AST-C (25 - 33%) than with AST-CC (13 - 17%).

### Structure of the Mollusca Receptors

**Figure 5:**
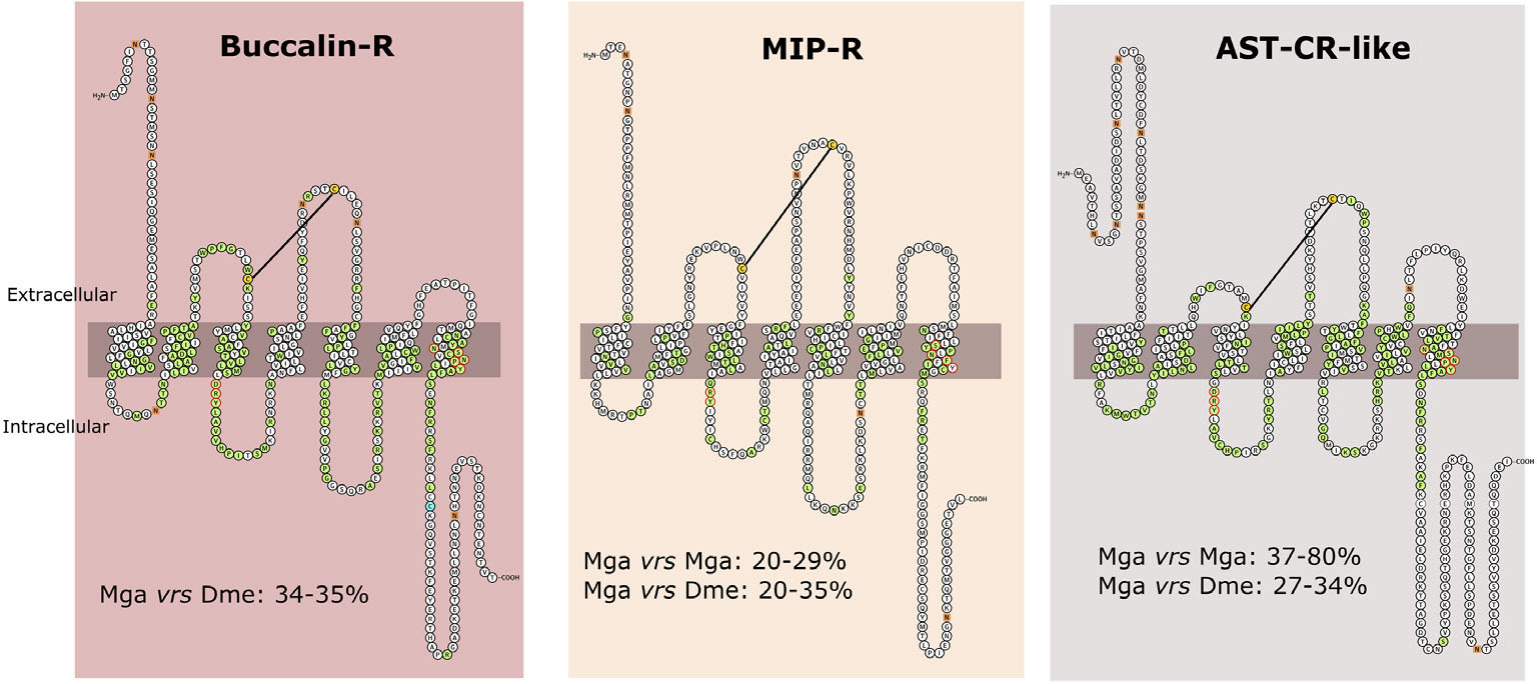
Structure and amino acid sequence conservation of the Mollusca Buccalin, MIP and AST-C-like receptors with the orthologues from other species. The figure represents the predicted receptor structure in the cell membrane. The receptors represented are the bivalve *M. galloprovincialis* Buccalin-R (VDI61602), MIP-R (VDI58805) and AST-CR-like (VDI08560). Receptor structure was obtained from Protter and the cell membrane is represented by the grey bar. Amino acids that were highly conserved across species and present in the full-length receptor sequence alignments (available from **Supplementary Figures 4**) are marked in green and the N-glycosylation motifs are represented by orange squares. The motif DRY and NSxxNPxxY (where x represents any aa) within TM7 that are required for receptor activation are outlined in red, the two conserved cysteine residues which might be responsible for the formation of a disulphide bond are highlighted in yellow and the C-terminal cysteine residue for potential palmitoylation after TM7 is in blue. The percentage (%) of amino acid sequence identity between the *M. galloprovincialis* (Mga) receptors and *D. melanogaster* (Dme) homologues is indicated.

The Mollusca receptors shared a conserved protein structure with their homologues from *D. melanogaster* and *H. sapiens* and receptors possessed seven transmembrane domains linked by three extracellular loops that alternated with three intracellular loops (**Figure 5** and **Supplementary Figures 4A–C**). Multiple sequence alignments of the mollusc receptors with the other protostome homologues revealed that the TM domains shared the highest sequence conservation across species. The conserved aa motifs involved in receptor activation, DRY in ICL2 between TM3 and TM4 and NSxxNPxxY (where x represents any aa) within TM7 were present (90, 91) (**Figure 5** and **Supplementary Figures 4A, C**). The exception was the protostome MIP-Rs in which aspartic acid of the motif DRY was mutated to QRY (Glutamine) and degeneration of the motif in TM7 occurred (**Figure 5** and **Supplementary Figure 4B**).

Two conserved cysteine residues, which potentially form a disulphide bond between ECL1 and ECL2 and determine receptor structure and stability were conserved in the mollusc and arthropod receptors. A C-terminal cysteine residue for potential palmitoylation after TM7, which is a characteristic of the rhodopsin-GPCRs, was also identified in the Buccalin-Rs and AST-CRs-like (**Figure 5**). No signal peptide was predicted for the *M. galloprovincialis* receptors or the *D. melanogaster* homologues except for DAR1. However, DeepLoc1 analysis predicted a cell membrane localization for the *M. galloprovincialis* receptors. Several N-glycosylation sites were found in the N-terminal region of *M. galloprovincialis* and other bivalve receptors (**Figure 5** and **Supplementary Figures 4A–C**).

The deduced protein sequence of *M. galloprovincialis* and *M. coruscus* buccalin-Rs shared 96% aa identity, 40 - 54% identity with the homologues from the Ostreidae family and 34 - 35% with the two receptor homologues in *D. melanogaster* (DAR1 and DAR2). The three *M. galloprovincialis* MIP-Rs were only 25 - 29% identical suggesting that they may have different functions. The *M. galloprovincialis* type I receptor was most similar to *D. melanogaster* AST-BR (35%), while the other paralogues type II and type III only shared 20% identity with *D. melanogaster* AST-BR. The amino acid sequence of the five *M. galloprovincialis* AST-CR-like shared between 37 to 80% identity and VDI08560.1 and VDI60978.1 (that arose due to a recent gene duplication) shared the highest aa sequence identity. The *M. galloprovincialis* and *M. coruscus* AST-CR-like shared 36 – 94% aa identity and 27 to 34% aa identity with the two *D. melanogaster* receptors.

### Comparisons of the Gene Environment of Buccalin-R And GALR-Like With the Homologue Regions in Human and *D. melanogaster*

**Figure 6:**
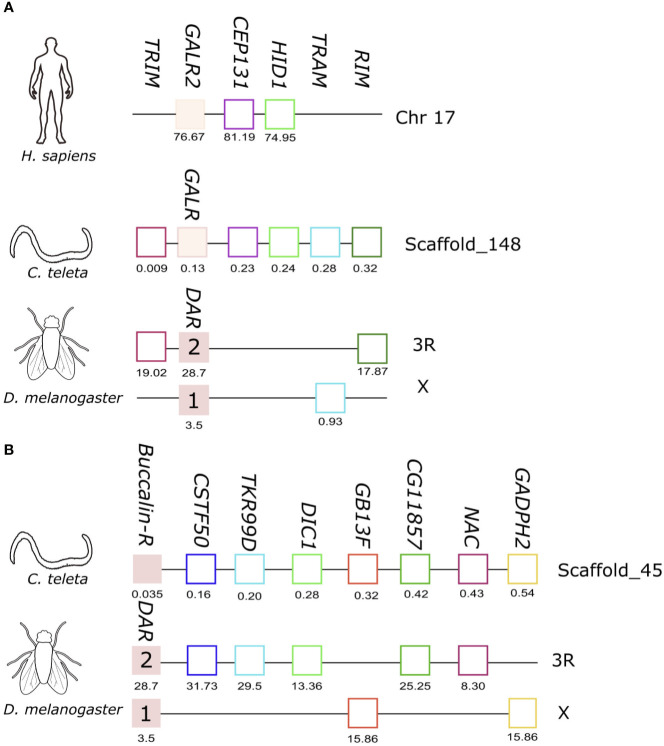
Short-range gene synteny of the *C*. *teleta GALR-like* **(A)** and *Buccalin-R* **(B)** with *D*. *melanogaster* and *H*. *sapiens*. Predicted genes are represented by coloured boxes and full blue boxes represent the genes of the *GALR* and *Buccalin-R*/*AST-AR* family. Orthologue neighbouring genes are indicated in the same colour. The genes are mapped on chromosomes based on their predicted position (Mbp) in the genome assemblies. Only genes that were conserved in the homologous genome regions are represented. Tripartite motif family (*TRIM*); *CCR4-NOT* transcription complex subunit 3 (*CNOT3*), Centrosomal Protein 131 (*CEP131*), HID1 Domain Containing (*HID1*), Translocation Associated Membrane Protein 1 (*TRAM*), Rab3 interacting molecule (*RIM*), WD repeat-containing protein 55 homolog (*CSTF50*), Tachykinin-like receptor at 99D (*TKR99D*), Mitochondrial dicarboxylate transporter (*DIC1*), Guanine nucleotide-binding protein subunit beta-1 (*GB13F*), NAC transcription factor (*NAC*), Glyceraldehyde -3phosphate dehydrogenase 2 (*GADPH2*).

Previously, our studies on the characterization of the Buccalin-R/AST-ARs and the sequence homologues of the vertebrate KISSR and GALRs in Arthropods and Mollusca suggested that they were most closely related to the metazoan KISSRs (31, 37). However, the topology of the present phylogenetic trees (**Figure 2A**) that included a larger number of molluscan representatives indicated that the protostomes, arthropod AST-ARs and lophotrochozoan Buccalin-Rs, tended to cluster with the vertebrate GALRs (**Figure 2A**). Moreover, a novel receptor clade containing homologues of the deuterostome GALRs were identified in molluscs (cephalopods), annelids and other lophotrochozoans. To gain further insight into the relationship of the lophotrochozoan Buccalin-R and GALR-like members with the KISSR/GALR, the gene environment in the annelid *C. teleta* and *O. bimaculoides*, which have the two receptor types was characterized (**Figure 6**). The *O. bimaculoides GALR-like* gene mapped to a short genome fragment (SuperContig KQ419625) and only 5 gene neighbours were mapped, and searches against *C. teleta* and the human genome regions failed to identify gene homologues in the neighbourhood of the *GALR-like* and *GALRs* genes.

In *C. teleta* the *GALR-like* homologue mapped to scaffold_148 and gene homologues of human *CEP131* and *HID1* located near the loci of human *GALR2* in chromosome 17 were identified (**Figure 6A**). In addition, homologues of three other neighbouring genes were found in *D. melanogaster AST-AR* chromosomes, *TRIM* and *RIM* in proximity with the *DAR2* gene on chromosome 3R and *TRAM* in proximity with *DAR1* on chromosome X. This suggests that the *C. teleta GALR-like* genome region shares similarity with both arthropod *AST-AR* and human *GALR2* genome regions.

The *C. teleta AST-AR* genome region was also characterized, and the gene mapped to scaffold_45 and shared conserved homologue neighbouring genes with the *D. melanogaster DAR1* and *DAR2* genome regions suggesting that both annelid *Buccalin-R* and insect *AST-AR* shared a common origin (**Figure 6B**). No homologue neighbouring genes of *C. teleta AST-AR* loci were found in the proximity of the human *GALR1* (chromosome 18), *GALR2*, *GALR3* (chromosome 22) or *KISS1R* (chromosome 19) genome regions. Comparison of the gene environment of human *KISSR* and *AST-ARs* in *D. melanogaster* (chromosomes 3R and X) and *A. gambiae* (chromosome 2R) using the conserved neighbouring genes previously identified, *PTBP1*, *EVIL5L*, *DOT1L* and *ODF3L2* (31), failed to retrieve putative homologues in the annelid *C. teleta KISSR*-like and *GALR-like* genome regions.

### Expression Analysis in the Bivalves and Targets of Immunity

**Figure 7:**
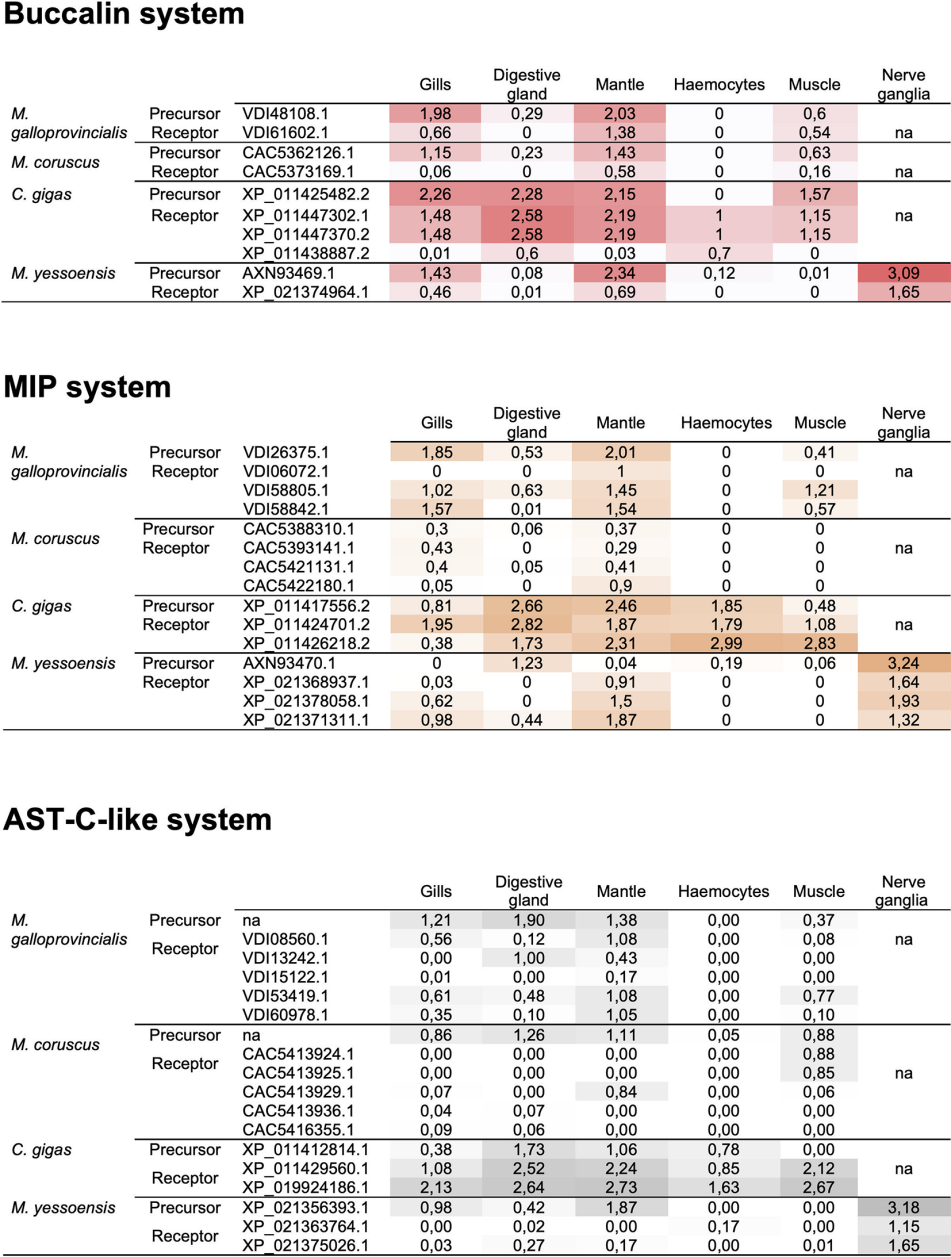
Expression levels (FPKM) of the Buccalin, MIP and AST-C-like systems in four bivalves. Data for *M. galloprovincialis*, *M. coruscus*, *C. gigas* and *M. yessoensis* was obtained by screening publicly available transcriptome data (SRA database) for gills, muscle, mantle, digestive gland/hepatopancreas, haemocytes and nerve ganglia (**Supplementary Table 2**). FPKM counts were calculated having in consideration the number of reads, gene length and the transcriptome sequencing depth. Only tissues common to all species are represented. Expression data for *C. gigas* mantle corresponded to the average of inner (SRX093415) and outer (SRX093411) transcriptomes and for the *M. yessoensis* muscle the average of smooth (n=3) and striated (n=3) muscles. Other *M. yessoensis* transcriptome data also reflect the average FPKM of 2 to 3 datasets (except the nerve ganglia, n = 1). Colour grading highlights differences in transcript abundance in the tissue libraries analysed. na- not available.

Transcriptome data available for the bivalves, *M. galloprovincialis*, *M. coruscus*, *C. gigas* and *M. yessoensis* (**Supplementary Table 2**) was used to infer potential AST system function in molluscs. The expression profile obtained suggested that buccalin, MIP and AST-C-like peptide precursors and receptors have a widespread tissue distribution and that they seemed to be most abundant in the mantle (**Figure 7**). Of the tissue transcriptomes analysed the haemocytes had the lowest expression except for the MIP system in *C. gigas*. The widespread tissue distribution and different relative abundance (RPKM) of the peptides and receptors suggests that they may have different functions (**Figure 7**). In *M. yessoensis* all homologues of the arthropod ASTs and receptors were present in the nerve ganglion (transcriptome, SRR6407589), and were far more abundant in this tissue than in the mantle (**Figure 6**). Analysis of a mantle transcriptome of *M. galloprovincialis* challenged with bacterial LPS revealed that only the AST-CR-like (VDI60978.1), was significantly down-regulated 12h post challenge (**Supplementary Figure 5**). These results suggest that the bivalve AST-C-like system may be involved in the immune response, as described in arthropods. To test this hypothesis *M. galloprovincialis* were challenged with the pathogenic marine bacteria *V. harveyi*.

### Exposure of *M. galloprovincialis* to an Immune Challenge and Response of the AST-C-Like System in Mantle

**Figure 8:**
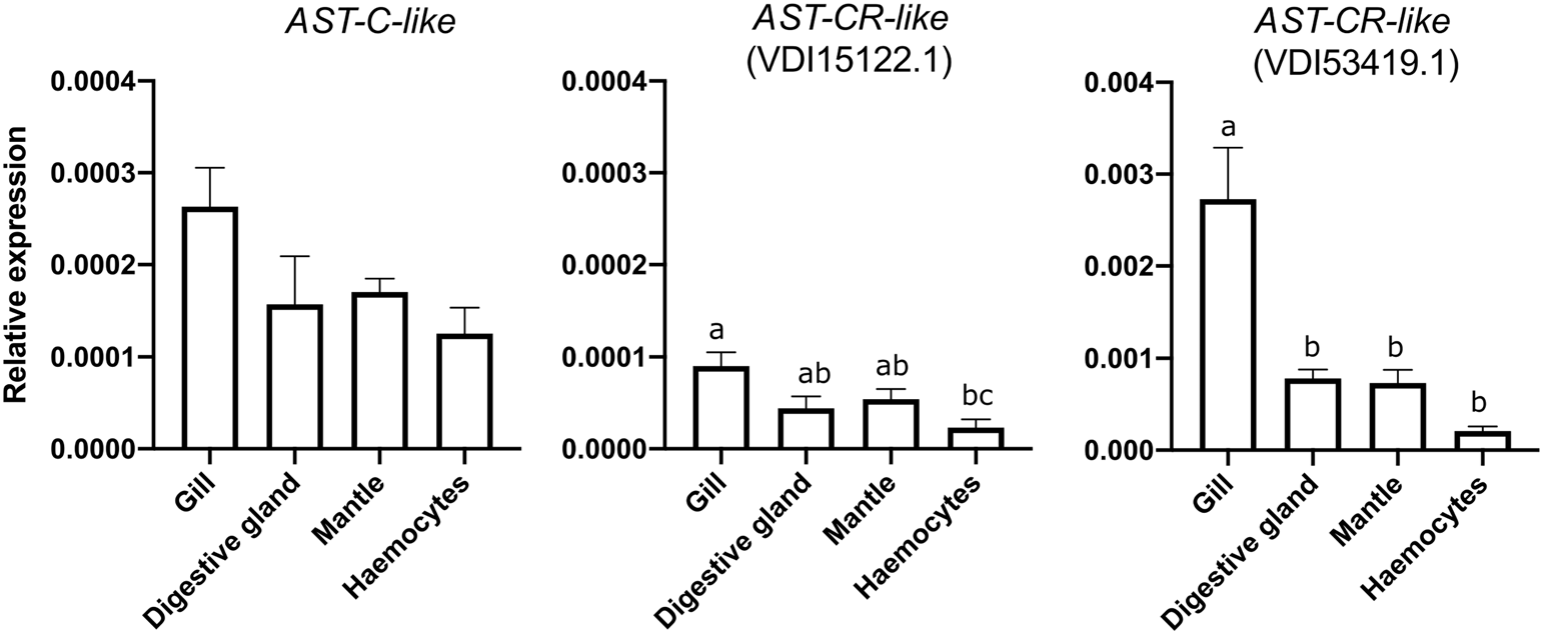
Tissue distribution of the AST-C-like precursor and two receptor transcripts in the bivalve *M. galloprovincialis*. Gene expression levels were normalized using the geometric mean of two reference genes (*EF1α* and *18S*). The results are represented as the mean ± SEM of three (n= 3) biological replicates. Significant differences are denoted by different letters and statistical analysis was performed using One-Way ANOVA in GraphPad Prism version 8.0.0 software for Mac OS X.

Quantitative PCR (qRT-PCR) analysis of *M. galloprovincialis* tissues confirmed the *AST-C-like* precursor and two *AST-CR-like* (VDI53419.1 and VDI15122.1) were detectable in the mantle (**Figure 8**). Other tissues such as the gills, the digestive gland and the haemocytes also expressed the peptide precursor and receptors. The abundance of *AST-C-like* was similar in all tissues analysed. *AST-CR-like* VDI15122.1 was significantly more expressed in the gills compared to haemocytes (p < 0.05) and *AST-CR-like* VDI53419.1 was significantly more expressed (p < 0.01) in the gills than in other tissues. *AST-CR-like* VDI53419.1 was most abundant and its expression in tissue was approximately 10-fold higher than the other receptor gene VDI15122.1 and the gene encoding the peptide precursor (**Figure 8**). No amplification product was obtained for VDI08560.1 and VDI13242.1. The *AST-CR-like* gene, VDI60978.1, which shared 97% identity with VDI08560.1 was not analysed as isoform specific qRT-PCR primers could not be designed.

**Figure 9:**
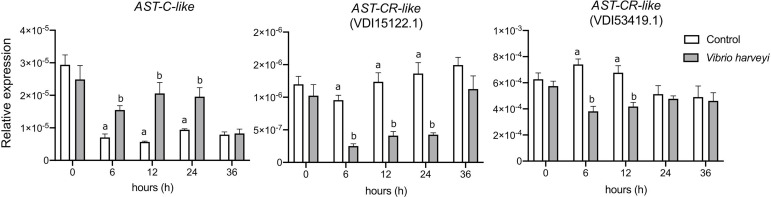
Effect of an immune challenge on AST-C-like precursor and receptor expression in the mantle of the bivalve *M. galloprovincialis*. Mantle edge samples were collected at 0, 6, 12, 24 and 36 hours after exposure to heat-inactivated *V. harveyi.* The expression of two *AST-CRs-like* (VDI15122.1 and VDI53419.1) was analysed. Gene expression levels were normalized using the geometric mean of two reference genes (*EF1α* and *18S*). The results are presented as the mean ± SEM of six (n= 6) biological replicates per group/sampling point. Significant differences between control and immune challenged mussel mantle at each timepoint are indicated by different letters and were detected using two-way ANOVA and a Sidak’s multiple comparison test in GraphPad Prism version 8.0.0 software for Mac OS X.

To assess if the AST-C-like system was modified by an immune challenge, the immune response of *M. galloprovincialis* to heat-killed *V. harveyi* was assessed by measuring by qRT-PCR the change in expression of innate immune-related genes. *TLRa* was significantly up-regulated 6h (p < 0.001) and 12 h (p < 0.05) after an immune challenge and *LYG1* was significantly down-regulated at 6 h (p < 0.05) and complement-like *C3-like* was not significantly changed (**Supplementary Figure 6**). The *AST-C-like* peptide precursor and receptor expression was modified after exposure to heat-killed *V. harveyi* (**Figure 9**). The *AST-C-like* precursor was significantly up-regulated 6 h (p < 0.05), 12 h (p < 0.0001) and 24 h (p <0.01) after an immune challenge. *AST-CR-like* VDI15122.1 was down-regulated at 6 h (p < 0.01), 12 h (p < 0.001) and 24 h (p < 0.0001) and *AST-CR-like* VDI53419.1 was down-regulated at 6 h (p < 0.0001) and 12 h (p < 0.01) after exposure to heat-killed *V. harveyi* (**Figure 9**). The qRT-PCR expression data corroborated the LPS transcriptome data of the AST-C-like system (peptide precursor and receptors) under an immune challenge in *M. galloprovincialis*.

## Discussion

The homologues of the arthropods AST-A, AST-B/MIP and AST-C systems and their function have been poorly described in the second largest animal phylum, the Mollusca. To better understand the peptide and receptor evolution and function a comparative approach was taken paying particular attention to the shelled molluscs (the bivalves) a group of ecologically and commercially important species. In molluscs, homologues of the three arthropod peptide groups (named buccalin, MIP and AST-C-like) and their corresponding receptors were identified. The sequence similarity between the mollusc and insect peptides and receptors was low but aa motifs involved in peptide and receptor structure were well conserved. In molluscs the peptide precursor and receptors evolved *via* lineage and species-specific events with receptor gene family expansions found in some species. The buccalin and MIP precursors encoded several mature peptides with differing aa sequences and sizes suggesting that they may have differing affinity or potencies for the corresponding receptors and may modulate distinct biological functions. Expression of all elements of the buccalin, MIP and AST-C-like systems were detected in the bivalve mantle and changes in the AST-C-like peptide and receptor transcripts in response to a bacterial immune challenge in *M. galloprovincialis* revealed that this neuropeptide system may contribute to the immune response in Mollusca.

### Diversity of Mollusca Peptides and Receptors

In molluscs homologue peptide precursors and receptors of the arthropod AST-systems were found. The potential peptides produced, and their putative receptors were more numerous than in *D. melanogaster* and other insects. In common with the insects and other arthropods AST-A and AST-B/MIP, the buccalin and MIP mature peptides in Mollusca were encoded by long protein precursors containing multiple mature peptides with distinct amino acid sequences but well conserved motifs important for bioactivity (18–20, 25, 37, 92). The results of phylogenetic analysis from the present and previous studies (15, 16, 37) revealed that the Mollusca and Arthropoda receptors shared a common origin. The general sequence and structural conservation of the mollusc peptides and receptors with the arthropod AST systems homologues suggests functional conservation is likely across the protostomes.

In molluscs, buccalins were originally isolated from neuronal elements in the accessory radula closer (ARC) muscle of the gastropod *A. californica* and regulate food-induced arousal (24). In arthropods including insects a single gene for the AST-A precursor has been described. In *D. melanogaster* the AST-A peptides share a conserved FGL-amide terminus but the sequences of the orthologue peptides in molluscs was very different although a conserved L-amide C-terminus existed but, in some gastropods and cephalopods I-amide C-terminal peptides were also found. A second AST-A peptide precursor previously proposed to contain buccalin-like peptides that terminated with a V-amide (37) was exclusive to molluscs and has recently been assigned to the LASGLV-amide family (19, 20, 25). The two buccalin precursors recently described in the gastropod Pacific abalone (*Haliotis discus hanna*) are authentic as they both encode C-terminal L-amide peptides (92) as well as the *C. ventricosus* and *P. maculate* precursors identified in this study. The longest and most peptide rich buccalin precursors were found within the gastropods, in the cephalopod *N. pompilius* and in the polyplacophore *A. granulata*. In bivalves, the number of buccalin L-amide peptides varied from 7 to 11 and peptide sequence conservation within each species precursor was distinct and may indicate they have different functions. Despite the large variety of putative buccalins only a single homologue of the arthropod AST-AR was found in most of the species analysed suggesting that alternative receptors activated by buccalins may exist. It would be interesting to test if molluscan buccalins are the cognate peptide activators of KISSR/GALR-like since protostome AST-A and AST-ARs are proposed to share a common evolutionary origin with the metazoan KISSR and GALRs (15, 16, 31, 37, 38). Putative KISSR-like and GALR-like were identified in molluscs and in annelids (this study and (31, 37) but our searches in Mollusca failed to identify orthologues in the genome of several of the species.

The MIP peptide precursors in molluscs in common with arthropod homologues encodes for multiple peptides with a similar structure. All mollusc and arthropod deduced mature peptides were C-terminal amides and possessed two conserved tryptophan residues previously shown to be important for peptide bioactivity (55). However, the consensus W(X_6/7_)W-amide sequence in Arthropoda AST-B/MIP peptides (8) was modified to W(X_4-7_)W-amide in Mollusca MIPs. Gastropod MIP precursors produced the largest variety of peptides and in *L. gigantea* 25 peptides were predicted, which was 5 times higher than in the insect *D. melanogaster*. Like the insects, *D. melanogaster* and *T. castaneum* a single AST-BR/MIPR was identified in most gastropods and in cephalopods. In contrast, bivalves possessed three receptor type genes that arose prior to the divergence of the molluscs and arthropods by gene duplication. In insects, AST-B/MIP share the same receptor as insect sex peptide, a.k.a. accessory gland peptide, which in *D. melanogaster* is 36 aa in length. The single gastropod *A. californica* MIP-R has been deorphanized but the species MIP peptides (23) and the newly identified bivalve MIP-Rs (type II and III) found in this study remain to be characterized with the MIP peptides as the obvious choice of ligand. The degeneration of functionally important rhodopsin-family receptor motifs such as DRY (in ICL2) and NSxxNPxxY (where x represents any aa) within TM7 previously reported in arthropod AST-BR/MIP-R also existed in the mollusc homologue receptors (88,87,8). MIP and MIP-R were present in all species analysed, except *B. glabrata* and it is unclear if this was due to the genome quality or if they were deleted from the genome.

In molluscs the AST-C-like peptide precursor generated a single peptide as observed in arthropods. The AST-C mature peptides were characterised by two conserved cysteine motifs in arthropods (19, 20, 25, 54, 92), which were proposed to determine peptide structure and function and they were also conserved in mollusc AST-C-like suggesting they may be important for function. Nonetheless, despite superficial sequence similarities the evolutionary model underpinning the AST-C/AST-C-like systems in arthropods and molluscs diverged. In arthropods three genes encode three different peptide precursors (AST-C, CC and CCC) (54, 93) and generally only one or two AST-CR exist (63, 94, 95). In the case of molluscs, a single gene encodes the peptide precursor (except in the gastropod *C. unifasciata* that contains two identical mature peptide genes), but a larger number of putative AST-CR-like exist compared to arthropods. In molluscs the receptor number was generally conserved apart from in the bivalves and specifically the Mytilidae family where lineage and species-specific events caused expansion of the AST-CR-like (type I). The oysters possessed the protostome AST-CR-like type I and an additional AST-CR-like type II that according to the phylogeny diverged from other lophotrochozoan receptors. Genome analysis of the lophotrochozoans revealed that AST-CR-like type II are tandem duplicates with the AST-CR-like type I gene suggesting that after gene duplication in the ancestral Ostreoidea they considerably diverged.

The identification in molluscs of homologues of the arthropod AST peptide precursors with the potential to produce multiple mature peptides with distinct sequences and of AST-Rs that emerged from lineage/species-specific expansions in molluscs suggests that the diversity and complexity of this neuropeptide system is higher than in arthropods. The functional role of the arthropod AST-like system in the molluscs and other Lophotrochozoans remains to be studied but its diversity suggests it may be pluripotent. In the future, deorphanization of the multiple receptors within the Mollusca with the corresponding peptides will help to understand the peptide-receptor interactions and provide a route towards the establishment of function.

### Evolutionary Scenarios for the Protostome AST-Like System and Discovery of GALR-Like Receptors in Molluscs

The lack of sequence conservation and distinct peptide precursor organization of the three protostome AST-like peptide families (AST-A/buccalin, AST-B/MIP and AST-C/AST-C-like) is indicative of their different evolutionary origins. This contrasts with the common evolutionary origin proposed for cognate receptor families. The presence of both peptide precursors and receptors in arthropods and molluscs suggested that they emerged prior to the ecdysozoans-lophotrochozoan divergence and shared a common origin and functional conservation with homologue systems in vertebrates. The protostome AST-A/Buccalin systems are homologues of the highly studied deuterostome GALR/KISS systems and the AST-C/AST-C-like system in protostomes is suggested to be the counterpart of the vertebrate SST-system (15, 31, 63–65, 95). For AST-B/MIP no homologue neuropeptide system has been described but receptor clustering with the vertebrate orphan receptors, GPR139/142, suggests that they may be evolutionary related. The function of GPR139/142 is poorly understood, and in mammals GPR139 is mostly expressed in the CNS and is suggested to regulate movement and food consumption/metabolism (96).

In our study we uncovered a novel mollusc GALR-like clade which only persisted in cephalopods. Previously we showed that putative GALR-like receptors were also present in annelids (31, 37) and the identification of homologues in other lophotrochozoans revealed that the GALR-like receptor clade emerged early but was selectively deleted from the genomes of several species. The GALR-like is the protostome orthologue of the deuterostome GALRs and changes the currently accepted paradigm that AST-AR/Buccalin-R are the protostome GALR representatives. In a previous study we proposed that the AST-ARs/Buccalin-Rs were more related with KISSR than with the GALRs. However, the inclusion of more family members in the present study clustered the mollusca and other lophotrochozoan Buccalin-Rs and insect AST-ARs closer to the GALRs suggesting an alternative evolutionary scenario. Comparisons of the gene environment of *AST-AR* and *GALRs* in the annelid (*C. teleta*), insect (*D. melanogaster*) and vertebrate (*H. sapiens*) revealed orthologue genes. The *GALR-like* genome region in *C. teleta* possessed genes shared in the genome region flanking arthropod *AST-AR* and human *GALR2* suggesting that the protostome *GALR-like* and vertebrate *GALR* genome regions are related and that the ancestral genes of metazoan *GALR* and protostome *AST-ARs* may have emerged from a common genome region in the early bilaterian genome prior to the protostome and deuterostome divergence. The *Buccalin-R* genome regions in *C. teleta* only shared genes with the equivalent *AST-AR* genome regions in *D. melanogaster* and no orthologues were found in human GALR or KISSR genome regions. Nonetheless, the evolutionary scenario that gave rise to the protostome ancestral AST-ARs, metazoan *KISSR* and GALR genes is difficult to resolve since protostomes and deuterostomes are suggested to have diverged approximately 535 million years ago (97, 98) according to fossil records and global, lineage and species-specific genome events may have blurred evolutionary traits.

### AST-C-Like System and Mantle Immune Function in *M. galloprovincialis*

In protostomes innate immunity is the main defence against pathogens. This system provides an immediate and effective response and depends on haemocytes that circulate in the haemolymph and humoral factors like lysozymes, complement-like molecules, peptidoglycan-recognition proteins (PGRPs) amongst others (71, 99, 100). Recent studies have revealed that immune factors in bivalves and molluscs are highly diverse due to species-specific expansions [(71, 101–106), Peng et al., submitted]. The diversity of bivalves, their adaptation to highly diverse environments, the plethora of pathogenic microorganism to which they are exposed, and their very different susceptibilities makes them excellent model systems to understand how environmental adaptation has shaped the immune system.

In arthropods, the role of the AST families in immunity is poorly resolved. In the haemolymph of the cockroach *Diploptera punctate* ASTs were suggested to be important regulatory peptides of the immune response (107) and in *Litopenaeus vannamei*, transcripts for the three AST peptide precursors (AST-A, AST-B/MIP and AST-C) were up-regulated in the haemocytes 6 hours after infection with the white spot syndrome virus (12). In *Scylla paramamosain* both AST-B/MIP and AST-BR/MIP-R were significantly induced in haemocytes and in the hepatopancreas after immune challenge and animals treated with AST-B/MIP had increased expression of pro-inflammatory cytokines and immune-effectors and receptor knock-down impaired the innate immune response to bacterial proliferation (14, 108). The only report that links the AST-C system to immunity was performed in *D. melanogaster* where ASTC-R1 and R2 were up-regulated by pathogenic bacteria and ASTC-R2 knock-down significantly decreased survival (13). The effect of the AST-C system was linked to modulation of the “*inhibition of immune deficiency*” (Imd) pathway (13). The Imd pathway controls anti-bacterial peptide gene expression in the fat body in response to gram-negative bacterial infections and the pathway is suggested to be similar to the mammalian TNF-α pathway (109, 110).

The orthologues of the arthropod AST families had a particularly high expression in the bivalve mantle (5, 68, 111–113) a mucosal surface where host-pathogen interactions are initially established (70). Expression analysis (qRT-PCR and transcriptome analysis) indicated that the AST-C-like system was highly expressed in the bivalve mantle and mantle transcriptome data revealed modified AST-CR-like expression after a bacterial LPS challenge. The activation of the immune response in *M. galloprovincialis* exposed to a pathogen challenge was revealed by the significantly modified mantle expression of *TLRa* and *LYG1* and was associated with a significant change in expression of the AST-C-like system. However, in contrast to what was described in *D. melanogaster* down-regulation of the receptors occurred in *M. galloprovincialis* mantle. The basis for the divergent response of the AST-C/AST-C-like system between *D. melanogaster* and *M. galloprovincialis* was not determined but may result from the character of the immune challenge used. In molluscs, homologues of the vertebrate TNF-α and TNFR have been described and respond to infection (114, 115). However, if the AST-C-like system in bivalves modulates TNF-α and TNFR is unstudied and further work using targeted gene knock-down approaches or overexpression studies and defining the peptide-receptor pairs and their response to different pathogens will be required to characterise in detail the molecular basis of the innate immune response.

## Conclusion

Peptide and receptor homologues of the arthropod AST-like families existed in molluscs. The diversity of AST peptides and receptor members found, and their widespread tissue distribution in molluscs suggests they are pluripotent factors that modulate a diversity of physiological processes. The mature peptides and receptor members (Buccalin, MIP and AST-C-like) were distinct across mollusc species, and this suggests that the AST families may be as complex as the neuropeptide system described in insects and other arthropods.

Phylogenetic analysis confirmed that receptors are specific to protostomes and emerged early in evolution prior to the Lophotrochozoa and Ecdysozoa divergence. In a previous study (37) we proposed that AST-ARs/Buccalin-Rs were most closely related to the metazoan KISSRs. In the present study by including AST-CRs in the phylogenetic analysis we found that they grouped with metazoan GALRs. Short-range gene linkage analysis of annelid genomes (the only protostomes where genes for Buccalin-R, GALR and KISSR persisted) confirmed that AST-AR and GALRs may be more evolutionary proximate, and we revised our previous evolutionary model for the protostome AST-ARs/Buccalin-Rs. In addition, we revealed for the first time the existence of Mollusca GALR-like genes in cephalopod genomes, and they were also present in annelids and a few other lophotrochozoans. This reveals that AST-AR/Buccalin-Rs evolved as a separate lineage and are not the orthologues of vertebrate GALRs as previously accepted.

Peptide precursors that encode for multiple mature peptides that diverge in sequence and the existence of lineage and species-specific duplicate receptors in molluscs suggests that the function of the Buccalin, MIP and AST-C-like systems may be distinct across species and that adaption to different environments may have affected gene evolution. Meta-analysis of tissue transcriptomes revealed that the Buccalin, MIP and AST-C-like families have a broad tissue distribution and varying abundance in several different bivalves, indicating it is an omniscient regulatory factor of the molluscan neuropeptide repertoire. The administration of an immune challenge to *M. galloprovincialis* significantly changed the expression of the AST-C-like peptide and receptor genes supporting its role in immunity and hinting at conservation of this function across protostomes. Overall, our study provides for the first time a comparison of the homologues of the three arthropod AST-systems across different molluscs and contributes to a better understanding of their evolution and function in protostomes.

## Data Availability Statement

The sequence, accession sequence numbers and datasets analyzed in this study are included in the Figures and also available in article/**Supplementary Material**. Further inquiries can be directed to the corresponding authors.

## Author Contributions

JC and DMP planned and supervised the study. JC and JI performed the bioinformatic and comparative analysis. ZL and MP performed the experimental challenge, transcriptome analysis and expression studies. JC and DMP analysed the results and wrote the manuscript. All authors contributed to the article and approved the submitted version.

## Funding

This study was funded by the Portuguese Foundation for Science and Technology (FCT) through project UIDB/04326/2020. ZL was supported by a PhD scholarship from the China Scholarship Council. MP was supported by a PhD scholarship from Shanghai Ocean University (China).

## Conflict of Interest

The authors declare that the research was conducted in the absence of any commercial or financial relationships that could be construed as a potential conflict of interest.

## Publisher’s Note

All claims expressed in this article are solely those of the authors and do not necessarily represent those of their affiliated organizations, or those of the publisher, the editors and the reviewers. Any product that may be evaluated in this article, or claim that may be made by its manufacturer, is not guaranteed or endorsed by the publisher.

## Supplementary Material

Supplementary Figure 1 Complete deduced sequences of the Mollusca **(A)** buccalins **(B)** MIPs and **(C)** AST-C-like peptide precursors. Each protein precursor encodes multiple small peptides except for AST-C-like where a single mature peptide exists. The predicted mature peptide sequences are highlighted in green. The protease cleavage sites are predicted based on the identification of dibasic residues (KR) and are in yellow. The predicted glycine that forms the C-terminal amide is annotated in blue. The predicted peptides were numbered with letters according to the order in which they appear in the precursors and are aligned in **Figure 3**. Sequences that were derived from mantle transcriptomes are annotated with a “*” and those deduced from the genome are indicated with “#”. The orthologue peptide precursors and the localization of the mature peptides in the brachiopod *L. anatina*, two annelids *P. dumerilii* and *C. teleta* and from the insects *D. melanogaster* and *T. castaneum* are also represented for comparison. The conserved amino acids that are important for the peptides structure and function are highlighted in bold.

Supplementary Figure 2 Deduced amino acid sequences of previously designated Mollusca buccalin-like precursors. The peptide precursors are now known to be members of the LASGLI/V-amide peptide family. Each precursor protein contains multiple small peptides. The predicted mature peptide sequences are highlighted in green. The protease cleavage sites are predicted based on the identification of dibasic residues (KR, RK) and are in yellow. The predicted glycine that forms the C-terminal amide are annotated in blue.

Supplementary Figure 3 Maximum Likelihood (ML) trees of the Molluscan receptors and homologues from other lophotrochozoans, ecdysozoans and deuterostomes. **(A)** Phylogenetic tree of the Buccalin-R/AST-ARs and AST-CR-like/AST-CRs (Rhodopsin γ family) and **(B)** Phylogenetic tree of MIP-Rs/AST-BRs (Rhodopsin β family). Trees were built with 100 bootstrap replicates and were performed with an LG model. The Rhodopsin γ family tree was mid-rooted based on the clustering of the sequences and the Rhodopsin β family tree was rooted with the *H. sapiens* (NP_000721) and *L. oculeatus* (XP_006629714) cholecystokinin receptor type A (CCKAR). Trees were displayed in FigTreev1.4.4 and edited in the Inkscape programme. Circled letters indicate: B- bivalves; G- gastropods, C- cephalopods; and P- polyplacophore species used in the analysis. The sequences that were retrieved from non-annotated genomes have the putative localization (Mbp) indicated.

Supplementary Figure 4 Multiple sequence alignments of Mollusca (bivalve, gastropod, and cephalopod) receptors with orthologues from annelids and *D. melanogaster*. **(A)** Buccalin-R/AST-AR, **(B)** MIP-R/AST-BR and **(C)** AST-CR-like/AST-CR. Receptor transmembrane domains are annotated within red boxes. Shading denotes amino acid conservation; dark grey represents 80% and black 100% conservation. Other sequence motifs suggested to be important for receptor function the motif DRY in ICL2 between TM3 and TM4 and NSxxNPxxY (where x represents any aa) within TM7 are marked with “*”. The putative disulphide bridge between the ECL1 and ECL2 is annotated and the C-terminal cysteine for potential palmitoylation after TM7 is marked with “#”. The N-terminal N-glycosylation sites conserved across the species are annotated with “.”. The accession number of receptor genes are indicated.

Supplementary Figure 5 Transcript abundance (FPKM) of the Buccalin, MIP and AST-CR-like peptide precursors and receptors in mantle edge transcriptomes from control and LPS challenged *M. galloprovincialis*. Data represents transcript abundance of the control (n= 3) and LPS challenged (n= 3) animals after 12h. The colour grades are indicative of transcript abundance, and the more intense colour indicates higher abundance. FPKM counts were calculated taking into consideration the number of reads, gene length and the transcriptome sequencing depth. The AST-CR (VDI60978.1) transcript highlighted in bold and italics was DE (p-adj < 0.05, log2-fold > 2) between the two conditions.

Supplementary Figure 6 Quantitative expression analysis of *TLRa*, *LYG1* and *C3-like* in the mantle edge of control and *V. harveyi* challenged *M. galloprovincialis*. Mantle samples at 0, 6 and 12 hours post exposure to the pathogen were analysed. The immune genes tested were *TLRa* (82), *LYG1* (83) and *C3-like* (Peng et al., submitted). Expression levels were normalized using the geometric mean of two reference genes (*EF1α* and *18S*). The results are represented as the mean ± SEM of three (n= 3) biological replicates per group/sampling point. Significant differences (*p< 0.05 and ***p< 0.001) between the control and immune challenged groups of the same sampling period were detected using two-way ANOVA and a Sidak’s multiple comparison test using GraphPad Prism version 8.0.0 software for Mac OS X.

Supplementary Table 1 Accession numbers of the mollusc, brachiopod and annelid peptide precursors and receptor genes. The genome localizations (scaffold or genome fragment) and positions (first coding exon, Mb) are indicated. Accession numbers that correspond to scaffolds and genome scaffolds are represented in italics. The species taxonomic classification was based on http://www.marinespecies.org/.

Supplementary Table 2 Accession numbers of bivalve sequence read archives (SRA) for RPKM analysis. SRA are available from https://www.ncbi.nlm.nih.gov/sra/.

## References

[1] Simakov O Marletaz F Cho SJ Edsinger-Gonzales E Havlak P Hellsten U. Insights Into Bilaterian Evolution From Three Spiralian Genomes. Nature (2013) 493:526–31. doi: 10.1038/nature11696 PMC4085046 23254933

[2] Raible F Tessmar-Raible K Osoegawa K Wincker P Jubin C Balavoine G. Evolution: Vertebrate-Type Intron-Rich Genes in the Marine Annelid Platynereis Dumerilii. Science (80) (2005) 310:1325–6. doi: 10.1126/science.1119089 16311335

[3] Miller DJ Ball EE. The Gene Complement of the Ancestral Bilaterian - Was Urbilateria a Monster? J Biol (2009) 8:10–3. doi: 10.1186/jbiol192 PMC2790832 19939290

[4] Takahashi T McDougall C Troscianko J Chen WC Jayaraman-Nagarajan A Shimeld SM. An EST Screen From the Annelid Pomatoceros Lamarckii Reveals Patterns of Gene Loss and Gain in Animals. BMC Evol Biol (2009) 9:1–17. doi: 10.1186/1471-2148-9-240 19781084 PMC2762978

[5] Wang S Zhang J Jiao W Li J Xun X Sun Y. Scallop Genome Provides Insights Into Evolution of Bilaterian Karyotype and Development. Nat Ecol Evol (2017) 1:1–12. doi: 10.1038/s41559-017-0120 28812685 PMC10970998

[6] Audsley N Weaver RJ. Neuropeptides Associated With the Regulation of Feeding in Insects. Gen Comp Endocrinol (2009) 162:93–104. doi: 10.1016/j.ygcen.2008.08.003 18775723

[7] Stay B Tobe SS. The Role of Allatostatins in Juvenile Hormone Synthesis in Insects and Crustaceans. Annu Rev Entomol (2007) 52:277–99. doi: 10.1146/annurev.ento.51.110104.151050 16968202

[8] Verlinden H Gijbels M Lismont E Lenaerts C Vanden Broeck J Marchal E. The Pleiotropic Allatoregulatory Neuropeptides and Their Receptors: A Mini-Review. J Insect Physiol (2015) 80:2–14. doi: 10.1016/j.jinsphys.2015.04.004 25982521

[9] Lorenz MW Kellner R Hoffmann KH. A Family of Neuropeptides That Inhibit Juvenile Hormone Biosynthesis in the Cricket, Gryllus Bimaculatus. J Biol Chem (1995) 270:21103–8. doi: 10.1074/jbc.270.36.21103 7673141

[10] Nässel DR. Neuropeptides in the Nervous System of Drosophila and Other Insects: Multiple Roles as Neuromodulators and Neurohormones. Prog Neurobiol (2002) 68:1–84. doi: 10.1016/S0301-0082(02)00057-6 12427481

[11] Wu K Li S Wang J Ni Y Huang W Liu Q. Peptide Hormones in the Insect Midgut. Front Physiol (2020) 11:191. doi: 10.3389/fphys.2020.00191 32194442 PMC7066369

[12] Wang F Li S Xiang J Li F. Transcriptome Analysis Reveals the Activation of Neuroendocrine-Immune System in Shrimp Hemocytes at the Early Stage of WSSV Infection. BMC Genomics (2019) 20:1–14. doi: 10.1186/s12864-019-5614-4 30922216 PMC6437892

[13] Bachtel ND Hovsepian GA Nixon DF Eleftherianos I. Allatostatin C Modulates Nociception and Immunity in Drosophila. Sci Rep (2018) 8:1–11. doi: 10.1038/s41598-018-25855-1 29760446 PMC5951828

[14] Xu Z Wei Y Wang G Ye H. B-Type Allatostatin Regulates Immune Response of Hemocytes in Mud Crab Scylla Paramamosain. Dev Comp Immunol (2021) 120:104050. doi: 10.1016/j.dci.2021.104050 33631272

[15] Mirabeau O Joly J-S. Molecular Evolution of Peptidergic Signaling Systems in Bilaterians. Proc Natl Acad Sci (2013) 110:E2028–37. doi: 10.1073/pnas.1219956110 PMC3670399 23671109

[16] Elphick MR Mirabeau O Larhammar D. Evolution of Neuropeptide Signalling Systems. J Exp Biol (2018) 221. doi: 10.1242/jeb.151092 PMC5818035 29440283

[17] Gerdol M Moreira R Cruz F Gómez-Garrido J Vlasova A Rosani U. Massive Gene Presence-Absence Variation Shapes an Open Pan-Genome in the Mediterranean Mussel. Genome Biol (2020) 21(1):275. doi: 10.1186/s13059-020-02180-3 33168033 PMC7653742

[18] Miller MW Beushausen S Cropper EC Eisinger K Stamm S Vilim FS. The Buccalin-Related Neuropeptides: Isolation and Characterization of an Aplysia cDNA Clone Encoding a Family of Peptide Cotransmitters. J Neurosci (1993) 13:3346–57. doi: 10.1523/jneurosci.13-08-03346.1993 PMC6576545 8101868

[19] De Oliveira AL Calcino A Wanninger A. Extensive Conservation of the Proneuropeptide and Peptide Prohormone Complement in Mollusks. Sci Rep (2019) 9:1–17. doi: 10.1038/s41598-019-40949-0 30890731 PMC6425005

[20] Veenstra JA. Neurohormones and Neuropeptides Encoded by the Genome of Lottia Gigantea, With Reference to Other Mollusks and Insects. Gen Comp Endocrinol (2010) 167:86–103. doi: 10.1016/j.ygcen.2010.02.010 20171220

[21] Stewart MJ Favrel P Rotgans BA Wang T Zhao M Sohail M. Neuropeptides Encoded by the Genomes of the Akoya Pearl Oyster Pinctata Fucata and Pacific Oyster Crassostrea Gigas: A Bioinformatic and Peptidomic Survey. BMC Genet (2014) 15:1–16. doi: 10.1186/1471-2164-15-840 25277059 PMC4200219

[22] Vilim FS Cropper EC Rosen SC Tenenbaum R Kupfermann I Weiss KR. Structure, Localization, and Action of Buccalin B: A Bioactive Peptide From Aplysia. Peptides (1994) 15:959–69. doi: 10.1016/0196-9781(94)90058-2 7991459

[23] Kim YJ Bartalska K Audsley N Yamanaka N Yapici N Lee JY. MIPs Are Ancestral Ligands for the Sex Peptide Receptor. Proc Natl Acad Sci USA (2010) 107:6520–5. doi: 10.1073/pnas.0914764107 PMC2851983 20308537

[24] Cropper EC Miller MW Tenenbaum R Kolks MA Kupfermann I Weiss KR. Structure and Action of Buccalin: A Modulatory Neuropeptide Localized to an Identified Small Cardioactive Peptide-Containing Cholinergic Motor Neuron of Aplysia Californica. Proc Natl Acad Sci USA (1988) 85:6177–81. doi: 10.1073/pnas.85.16.6177 PMC281928 3413086

[25] Zhang M Wang Y Li Y Li W Li R Xie X. Identification and Characterization of Neuropeptides by Transcriptome and Proteome Analyses in a Bivalve Mollusc Patinopecten Yessoensis. Front Genet (2018) 9:197. doi: 10.3389/fgene.2018.00197 29922332 PMC5996578

[26] Adamson KJ Wang T Zhao M Bell F Kuballa AV Storey KB. Molecular Insights Into Land Snail Neuropeptides Through Transcriptome and Comparative Gene Analysis. BMC Genomics (2015) 16:1–15. doi: 10.1186/s12864-015-1510-8 25884396 PMC4408573

[27] Ahn SJ Martin R Rao S Choi MY. Neuropeptides Predicted From the Transcriptome Analysis of the Gray Garden Slug Deroceras Reticulatum. Peptides (2017) 93:51–65. doi: 10.1016/j.peptides.2017.05.005 28502716

[28] Pratt GE Farnsworth DE Siegel NR Fok KF Feyereisen R. Identification of an Allatostatin From Adult Diploptera Punctata. Biochem Biophys Res Commun (1989) 163:1243–7. doi: 10.1016/0006-291X(89)91111-X 2783135

[29] Woodhead AP Stay B Seidel SL Khan MA Tobe SS. Primary Structure of Four Allatostatins: Neuropeptide Inhibitors of Juvenile Hormone Synthesis. Proc Natl Acad Sci USA (1989) 86:5997–6001. doi: 10.1073/pnas.86.15.5997 2762309 PMC297759

[30] Chen J Reiher W Hermann-Luibl C Sellami A Cognigni P Kondo S. Allatostatin A Signalling in Drosophila Regulates Feeding and Sleep and Is Modulated by PDF. PLoS Genet (2016) 12:1–33. doi: 10.1371/journal.pgen.1006346 PMC5045179 27689358

[31] Felix RC Trindade M Pires IRP Fonseca VG Martins RS Silveira H. Unravelling the Evolution of the Allatostatin-Type A, KISS and Galanin Peptide-Receptor Gene Families in Bilaterians: Insights From Anopheles Mosquitoes. PLoS One (2015) 10. doi: 10.1371/journal.pone.0130347 PMC4489612 26135459

[32] Fusé M Zhang JR Partridge E Nachman RJ Orchard I Bendena WG. Effects of an Allatostatin and a Myosuppressin on Midgut Carbohydrate Enzyme Activity in the Cockroach Diploptera Punctata. Peptides (1999) 20:1285–93. doi: 10.1016/S0196-9781(99)00133-3 10612442

[33] Robertson L Rodriguez EP Lange AB. The Neural and Peptidergic Control of Gut Contraction in Locusta Migratoria: The Effect of an FGLa/AST. J Exp Biol (2012) 215:3394–402. doi: 10.1242/jeb.073189 22693021

[34] Vanderveken M O’Donnell MJ. Effects of Diuretic Hormone 31, Drosokinin, and Allatostatin a on Transepithelial K+ Transport and Contraction Frequency in the Midgut and Hindgut of Larval Drosophila Melanogaster. Arch Insect Biochem Physiol (2014) 85:76–93. doi: 10.1002/arch.21144 24408875

[35] Hentze JL Carlsson MA Kondo S Nässel DR Rewitz KF. The Neuropeptide Allatostatin a Regulates Metabolism and Feeding Decisions in Drosophila. Sci Rep (2015) 5:1–16. doi: 10.1038/srep11680 PMC4485031 26123697

[36] Christ P Hill SR Schachtner J Hauser F Ignell R. Functional Characterization of the Dual Allatostatin-A Receptors in Mosquitoes. Peptides (2018) 99:44–55. doi: 10.1016/j.peptides.2017.10.016 29103918

[37] Cardoso JCR Félix RC Bjärnmark N Power DM. Allatostatin-Type A, Kisspeptin and Galanin GPCRs and Putative Ligands as Candidate Regulatory Factors of Mantle Function. Mar Genomics (2016) 27. doi: 10.1016/j.margen.2015.12.003 26751715

[38] Lenz C Søndergaard L Grimmelikhuijzen CJP. Molecular Cloning and Genomic Organization of a Novel Receptor From Drosophila Melanogaster Structurally Related to Mammalian Galanin Receptors. Biochem Biophys Res Commun (2000) 269:91–6. doi: 10.1006/bbrc.2000.2251 10694483

[39] In VV Ntalamagka N O’Connor W Wang T Powell D Cummins SF. Reproductive Neuropeptides That Stimulate Spawning in the Sydney Rock Oyster (Saccostrea Glomerata). Peptides (2016) 82:109–19. doi: 10.1016/j.peptides.2016.06.007 27328253

[40] Vanden Broeck J. Neuropeptides and Their Precursors in the Fruitfly, Drosophila Melanogaster. Peptides (2001) 22:241–54. doi: 10.1016/S0196-9781(00)00376-4 11179818

[41] Schoofs L Holman GM Hayes TK Nachman RJ De Loof A. Isolation, Identification and Synthesis of Locustamyoinhibiting Peptide (LOM-MIP), a Novel Biologically Active Neuropeptide From Locusta Migratoria. Regul Pept (1991) 36:111–9. doi: 10.1016/0167-0115(91)90199-Q 1796179

[42] Hua YJ Tanaka Y Nakamura K Sakakibara M Nagata S Kataoka H. Identification of a Prothoracicostatic Peptide in the Larval Brain of the Silkworm, Bombyx Mori. J Biol Chem (1999) 274:31169–73. doi: 10.1074/jbc.274.44.31169 10531308

[43] Kim YJ Žitňan D Cho KH Schooley DA Misoguchi A Adams ME. Central Peptidergic Ensembles Associated With Organization of an Innate Behavior. Proc Natl Acad Sci USA (2006) 103:14211–6. doi: 10.1073/pnas.0603459103 PMC1599936 16968777

[44] Kolodziejczyk A Nässel DR. A Novel Wide-Field Neuron With Branches in the Lamina of the Drosophila Visual System Expresses Myoinhibitory Peptide and May Be Associated With the Clock. Cell Tissue Res (2011) 343:357–69. doi: 10.1007/s00441-010-1100-7 21174124

[45] Schulze J Neupert S Schmidt L Predel R Lamkemeyer T Homberg U. Myoinhibitory Peptides in the Brain of the Cockroach Leucophaea Maderae and Colocalization With Pigment-Dispersing Factor in Circadian Pacemaker Cells. J Comp Neurol (2012) 520:1078–97. doi: 10.1002/cne.22785 22095637

[46] Schoofs L Veelaert D Vanden BJ De Loof A. Immunocytochemical Distribution of Locustamyoinhibiting Peptide (Lom-MIP) in the Nervous System of Locusta Migratoria. Regul Pept (1996) 63:171–9. doi: 10.1016/S0167-0115(96)00040-7 8837226

[47] Davis NT Blackburn MB Golubeva EG Hildebrand JG. Localization of Myoinhibitory Peptide Immunoreactivity in Manduca Sexta and Bombyx Mori, With Indications That the Peptide Has a Role in Molting and Ecdysis. J Exp Biol (2003) 206:1449–60. doi: 10.1242/jeb.00234 12654884

[48] Lange AB Alim U Vandersmissen HP Mizoguchi A Vanden BJ Orchard I. The Distribution and Physiological Effects of the Myoinhibiting Peptides in the Kissing Bug, Rhodnius Prolixus. Front Neurosci (2012) 6:98. doi: 10.3389/fnins.2012.00098 22783161 PMC3390896

[49] Williams EA Conzelmann M Jékely G. Myoinhibitory Peptide Regulates Feeding in the Marine Annelid Platynereis. Front Zool (2015) 12. doi: 10.1186/s12983-014-0093-6 PMC4307165 25628752

[50] Vandersmissen HP Nachman RJ Vanden Broeck J. Sex Peptides and MIPs Can Activate the Same G Protein-Coupled Receptor. Gen Comp Endocrinol (2012) 188:137–43. doi: 10.1016/j.ygcen.2013.02.014 23453963

[51] Yamanaka N Hua YJ Roller L Spalovská-Valachová I Mizoguchi A Kataoka H. Bombyx Prothoracicostatic Peptides Activate the Sex Peptide Receptor to Regulate Ecdysteroid Biosynthesis. Proc Natl Acad Sci USA (2010) 107:2060–5. doi: 10.1073/pnas.0907471107 PMC2836647 20133850

[52] Kramer SJ Toschi A Miller CA Kataoka H Quistad GB Li JP. Identification of an Allatostatin From the Tobacco Hornworm Manduca Sexta. Proc Natl Acad Sci USA (1991) 88:9458–62. doi: 10.1073/pnas.88.21.9458 PMC52737 1946359

[53] Veenstra JA. Allatostatin C and Its Paralog Allatostatin Double C: The Arthropod Somatostatins. Insect Biochem Mol Biol (2009) 39:161–70. doi: 10.1016/j.ibmb.2008.10.014 19063967

[54] Veenstra JA. Allatostatins C, Double C and Triple C, the Result of a Local Gene Triplication in an Ancestral Arthropod. Gen Comp Endocrinol (2016) 230–231:153–7. doi: 10.1016/j.ygcen.2016.04.013 27102937

[55] Coast GM Schooley DA. Toward a Consensus Nomenclature for Insect Neuropeptides and Peptide Hormones. Peptides (2011) 32:620–31. doi: 10.1016/j.peptides.2010.11.006 21093513

[56] Weaver RJ Audsley N. Neuropeptide Regulators of Juvenile Hormone Synthesis: Structures, Functions, Distribution, and Unanswered Questions. Ann NY Acad Sci (2009) 1163:316–29. doi: 10.1111/j.1749-6632.2009.04459.x 19456353

[57] Price MD Merte J Nichols R Koladich PM Tobe SS Bendena WG. Drosophila Melanogaster Flatline Encodes a Myotropin Orthologue to Manduca Sexta Allatostatin. Peptides (2002) 23:787–94. doi: 10.1016/S0196-9781(01)00649-0 11897399

[58] Matthews HJ Audsley N Weaver RJ. In Vitro and In Vivo Effects of Myo-Active Peptides on Larvae of the Tomato Moth Lacanobia Oleracea and the Cotton Leaf Worm Spodoptera Littoralis (Lepidoptera; Noctuidae). Arch Insect Biochem Physiol (2008) 69:60–9. doi: 10.1002/arch.20265 18780345

[59] Down RE Matthews HJ Audsley N. Effects of Manduca Sexta Allatostatin and an Analog on the Pea Aphid Acyrthosiphon Pisum (Hemiptera: Aphididae) and Degradation by Enzymes From the Aphid Gut. Peptides (2010) 31:489–97. doi: 10.1016/j.peptides.2009.06.017 19560498

[60] Matthews HJ Down RE Audsley N. Effects of Manduca Sexta Allatostatin and an Analogue on the Peach-Potato Aphid Myzus Persicae (Hemiptera: Aphididae) and Degradation by Enzymes in the Aphid Gut. Arch Insect Biochem Physiol (2010) 75:139–57. doi: 10.1002/arch.20376 20936640

[61] Díaz MM Schlichting M Abruzzi KC Long X Rosbash M. Allatostatin-C/AstC-R2 Is a Novel Pathway to Modulate the Circadian Activity Pattern in Drosophila. Curr Biol (2019) 29:13–22.e3. doi: 10.1016/j.cub.2018.11.005 30554904 PMC6325008

[62] Liu A Liu F Shi W Huang H Wang G Ye H. C-Type Allatostatin and Its Putative Receptor From the Mud Crab Serve an Inhibitory Role in Ovarian Development. J Exp Biol (2019) 222. doi: 10.1242/jeb.207985 31558587

[63] Kreienkamp HJ Larusson HJ Witte I Roeder T Birgül N Hönck HH. Functional Annotation of Two Orphan G-Protein-Coupled Receptors, Drostar1 and -2, From Drosophila Melanogaster and Their Ligands by Reverse Pharmacology. J Biol Chem (2002) 277:39937–43. doi: 10.1074/jbc.M206931200 12167655

[64] Cardoso JC Félix RC Fonseca VG Power DM. Feeding and the Rhodopsin Family G-Protein Coupled Receptors in Nematodes and Arthropods. Front Endocrinol (Lausanne) (2012) 3:157. doi: 10.3389/fendo.2012.00157 23264768 PMC3524798

[65] Jékely G. Global View of the Evolution and Diversity of Metazoan Neuropeptide Signaling. Proc Natl Acad Sci USA (2013) 110:8702–7. doi: 10.1073/pnas.1221833110 PMC3666674 23637342

[66] Cardoso JCR Ferreira V Zhang X Anjos L Félix RC Batista FM. Evolution and Diversity of Alpha-Carbonic Anhydrases in the Mantle of the Mediterranean Mussel (Mytilus Galloprovincialis). Sci Rep (2019) 9. doi: 10.1038/s41598-019-46913-2 PMC6639325 31320702

[67] Cardoso JCR Félix RC Ferreira V Peng MX Zhang X Power DM. The Calcitonin-Like System Is an Ancient Regulatory System of Biomineralization. Sci Rep (2020) 10:7581 . doi: 10.1038/s41598-020-64118-w 32371888 PMC7200681

[68] Bjärnmark NA Yarra T Churcher AM Felix RC Clark MS Power DM. Transcriptomics Provides Insight Into Mytilus Galloprovincialis (Mollusca: Bivalvia) Mantle Function and its Role in Biomineralisation. Mar Genomics (2016) 27:37–45. doi: 10.1016/j.margen.2016.03.004 27037218

[69] Clark MS Peck LS Arivalagan J Backeljau T Berland S Cardoso JCR. Deciphering Mollusc Shell Production: The Roles of Genetic Mechanisms Through to Ecology, Aquaculture and Biomimetics. Biol Rev (2020) 95(6):1812–37. doi: 10.1111/brv.12640 32737956

[70] Allam B Pales Espinosa E. Bivalve Immunity and Response to Infections: Are We Looking at the Right Place? Fish Shellfish Immunol (2016) 53:4–12. doi: 10.1016/j.fsi.2016.03.037 27004953

[71] Allam B Raftos D. Immune Responses to Infectious Diseases in Bivalves. J Invertebr Pathol (2015) 131:121–36. doi: 10.1016/j.jip.2015.05.005 26003824

[72] Beaz-Hidalgo R Balboa S Romalde JL Figueras MJ. Diversity and Pathogenecity of Vibrio Species in Cultured Bivalve Molluscs. Environ Microbiol Rep (2010) 2:34–43. doi: 10.1111/j.1758-2229.2010.00135.x 23765996

[73] Romero A Del Mar Costa M Forn-Cuni G Balseiro P Chamorro R Dios S. Occurrence, Seasonality and Infectivity of Vibrio Strains in Natural Populations of Mussels Mytilus Galloprovincialis. Dis Aquat Organ (2014) 108:149–163. doi: 10.3354/dao02701 24553420

[74] Zhang X-H He X Austin B. Vibrio Harveyi: A Serious Pathogen of Fish and Invertebrates in Mariculture. Mar Life Sci Technol (2020) 2:231–45. doi: 10.1007/s42995-020-00037-z PMC7223180 32419972

[75] Larsson A. AliView: A Fast and Lightweight Alignment Viewer and Editor for Large Datasets. Bioinformatics (2014) 30(22):3276–8. doi: 10.1093/bioinformatics/btu531 PMC4221126 25095880

[76] Miller MA Pfeiffer W Schwartz T. Creating the CIPRES Science Gateway for Inference of Large Phylogenetic Trees. 2010 Gatew Comput Environ Work GCE 2010 (2010). doi: 10.1109/GCE.2010.5676129

[77] Ronquist F Teslenko M van der Mark P Ayres DL Darling A Höhna S. Mrbayes 3.2: Efficient Bayesian Phylogenetic Inference and Model Choice Across a Large Model Space. Syst Biol (2012) 61(3):539–642. doi: 10.1093/sysbio/sys029 22357727 PMC3329765

[78] Stamatakis A. RAxML Version 8: A Tool for Phylogenetic Analysis and Post-Analysis of Large Phylogenies. Bioinformatics (2014) 30:1312–3. doi: 10.1093/bioinformatics/btu033 PMC3998144 24451623

[79] Thiel D Yañez-Guerra LA Franz-Wachtel M Hejnol A Jékely G. Nemertean, Brachiopod, and Phoronid Neuropeptidomics Reveals Ancestral Spiralian Signaling Systems. Mol Biol Evol (2021) 1–20. doi: 10.1093/molbev/msab211 34272863 PMC8557429

[80] Almagro Armenteros JJ Tsirigos KD Sønderby CK Petersen TN Winther O Brunak S. SignalP 5.0 Improves Signal Peptide Predictions Using Deep Neural Networks. Nat Biotechnol (2019) 37:420–3. doi: 10.1038/s41587-019-0036-z 30778233

[81] Almagro Armenteros JJ Sønderby CK Sønderby SK Nielsen H Winther O. DeepLoc: Prediction of Protein Subcellular Localization Using Deep Learning. Bioinformatics (2017) 33:3387–95. doi: 10.1093/bioinformatics/btx431 29036616

[82] Xu M Wu J Ge D Wu C Changfeng C Lv Z. A Novel Toll-Like Receptor From Mytilus Coruscus Is Induced in Response to Stress. Fish Shellfish Immunol (2018) 78:331–7. doi: 10.1016/j.fsi.2018.04.058 29709593

[83] Wang Q Zhang L Zhao J You L Wu H. Two Goose-Type Lysozymes in Mytilus Galloprovincialis: Possible Function Diversification and Adaptive Evolution. PLoS One (2012) 7(9):e45148. doi: 10.1371/journal.pone.0045148 23028813 PMC3448621

[84] Rayko M Komissarov A Kwan JC Lim-Fong G Rhodes AC Kliver S. Draft Genome of Bugula Neritina, a Colonial Animal Packing Powerful Symbionts and Potential Medicines. Sci Data (2020) 7:1–5. doi: 10.1038/s41597-020-00684-y 33082320 PMC7576161

[85] Kocot KM Struck TH Merkel J Waits DS Todt C Brannock PM. Phylogenomics of Lophotrochozoa With Consideration of Systematic Error. Syst Biol (2017) 66:256–82. doi: 10.1093/sysbio/syw079 27664188

[86] Wanninger A Wollesen T. The Evolution of Molluscs. Biol Rev (2019) 94:102–15. doi: 10.1111/brv.12439 PMC6378612 29931833

[87] Tanner AR Fuchs D Winkelmann IE Gilbert MTP Pankey MS Ribeiro ÂM. Molecular Clocks Indicate Turnover and Diversification of Modern Coleoid Cephalopods During the Mesozoic Marine Revolution. Proc R Soc B Biol Sci (2017) 284:1–7. doi: 10.1098/rspb.2016.2818 PMC5360930 28250188

[88] Liu Z Xu Y Wu L Zhang S. Evolution of Galanin Receptor Genes: Insights From the Deuterostome Genomes. J Biomol Struct Dyn (2010) 28:97–106. doi: 10.1080/07391102.2010.10507346 20476798

[89] Kim DK Yun S Son GH Hwang JI Park CR Kim J. Coevolution of the Spexin/Galanin/Kisspeptin Family: Spexin Activates Galanin Receptor Type II and III. Endocrinology (2014) 155:1864–73. doi: 10.1210/en.2013-2106 24517231

[90] Rovati GE Capra V Neubig RR. The Highly Conserved DRY Motif of Class A G Protein-Coupled Receptors: Beyond the Ground State. Mol Pharmacol (2007) 71:959–64. doi: 10.1124/mol.106.029470 17192495

[91] Fritze O Filipek S Kuksa V Palczewski K Hofmann KP Ernst OP. Role of the Conserved NPxxY(x)5,6F Motif in the Rhodopsin Ground State and During Activation. Proc Natl Acad Sci USA (2003) 100:2290–5. doi: 10.1073/pnas.0435715100 PMC151333 12601165

[92] Sharker MR Sukhan ZP Kim SC Rha SJ Kho KH. In Silico Prediction of Neuropeptides From the Neural Ganglia of Pacific Abalone Haliotis Discus Hannai (Mollusca: Gastropoda). Eur Zool J (2020) 87:35–45. doi: 10.1080/24750263.2019.1708485

[93] Chang J Zhao J Tian X. In Silico Prediction of Neuropeptides in Hymenoptera Parasitoid Wasps. PLoS One (2018) 13:1–15. doi: 10.1371/journal.pone.0193561 PMC5831470 29489917

[94] Mayoral JG Nouzova M Brockhoff A Goodwin M Hernandez-Martinez S Richter D. Allatostatin-C Receptors in Mosquitoes. Peptides (2010) 31:442–50. doi: 10.1016/j.peptides.2009.04.013 PMC2826609 19409436

[95] Caers J Verlinden H Zels S Vandersmissen HP Vuerinckx K Schoofs L. More Than Two Decades of Research on Insect Neuropeptide GPCRs: An Overview. Front Endocrinol (Lausanne) (2012) 3:151. doi: 10.3389/fendo.2012.00151 23226142 PMC3510462

[96] Vedel L Nøhr AC Gloriam DE Bräuner-Osborne H. Pharmacology and Function of the Orphan GPR139 G Protein-Coupled Receptor. Basic Clin Pharmacol Toxicol (2020) 126:35–46. doi: 10.1111/bcpt.13263 31132229 PMC7318219

[97] Budd GE Jensen S. The Origin of the Animals and a ‘Savannah’ Hypothesis for Early Bilaterian Evolution. Biol Rev (2017) 92:446–73. doi: 10.1111/brv.12239 26588818

[98] Budd GE Mann RP. Survival and Selection Biases in Early Animal Evolution and a Source of Systematic Overestimation in Molecular Clocks: Biases in Evolution. Interface Focus (2020) 10(4):20190110. doi: 10.1098/rsfs.2019.0110 32637066 PMC7333906

[99] Bouallegui Y. Immunity in Mussels: An Overview of Molecular Components and Mechanisms With a Focus on the Functional Defenses. Fish Shellfish Immunol (2019) 89:158–69. doi: 10.1016/j.fsi.2019.03.057 30930277

[100] Schultz JH Adema CM. Comparative Immunogenomics of Molluscs. Dev Comp Immunol (2017) 75:3–15. doi: 10.1016/j.dci.2017.03.013 28322934 PMC5494275

[101] Zhang L Li L Guo X Litman GW Dishaw LJ Zhang G. Massive Expansion and Functional Divergence of Innate Immune Genes in a Protostome. Sci Rep (2015) 5:8693. doi: 10.1038/srep08693 25732911 PMC4346834

[102] Gerdol M. Immune-Related Genes in Gastropods and Bivalves: A Comparative Overview. Invertebr Surviv J (2017) 14:95–111. doi: 10.25431/1824-307X/isj.v14i1.103-118

[103] Moreira R Balseiro P Forn-Cuní G Milan M Bargelloni L Novoa B. Bivalve Transcriptomics Reveal Pathogen Sequences and a Powerful Immune Response of the Mediterranean Mussel (Mytilus Galloprovincialis). Mar Biol (2018) 165:1–20. doi: 10.1007/s00227-018-3308-0

[104] Batista FM Churcher AM Manchado M Leitão A Power DM. Uncovering the Immunological Repertoire of the Carpet Shell Clam Ruditapes Decussatus Through a Transcriptomic-Based Approach. Aquac Fish (2019) 4:37–42. doi: 10.1016/j.aaf.2018.03.003

[105] Rosani U Domeneghetti S Gerdol M Pallavicini A Venier P. Expansion and Loss Events Characterized the Occurrence of MIF-Like Genes in Bivalves. Fish Shellfish Immunol (2019) 93:39–49. doi: 10.1016/j.fsi.2019.07.019 31306763

[106] Vogeler S Carboni S Li X Joyce A. Phylogenetic Analysis of the Caspase Family in Bivalves: Implications for Programmed Cell Death, Immune Response and Development. BMC Genomics (2021) 22:1–17. doi: 10.1186/s12864-021-07380-0 33494703 PMC7836458

[107] Skinner JR Fairbairn SE Woodhead AP Bendena WG Stay B. Allatostatin in Hemocytes of the Cockroach Diploptera Punctata. Cell Tissue Res (1997) 290:119–28. doi: 10.1007/s004410050914 9377632

[108] Xu Z Wei Y Guo S Lin D Ye H. B-Type Allatostatin Modulates Immune Response in Hepatopancreas of the Mud Crab Scylla Paramamosain. Dev Comp Immunol (2020) 110:103725. doi: 10.1016/j.dci.2020.103725 32376281

[109] Khush RS Leulier F Lemaitre B. Drosophila Immunity: Two Paths to NF-κb. Trends Immunol (2001) 22:260–4. doi: 10.1016/S1471-4906(01)01887-7 11323284

[110] Davoodi S Galenza A Panteluk A Deshpande R Ferguson M Grewal S. The Immune Deficiency Pathway Regulates Metabolic Homeostasis in Drosophila. J Immunol (2019) 202:2747–59. doi: 10.4049/jimmunol.1801632 30902902

[111] Yang JL Feng DD Liu J Xu JK Chen K Li YF. Chromosome-Level Genome Assembly of the Hard-Shelled Mussel Mytilus Coruscus, a Widely Distributed Species From the Temperate Areas of East Asia. Gigascience (2021) 10:1–13. doi: 10.1093/gigascience/giab024 PMC8063583 33891010

[112] Wang J Zhang G Fang X Guo X Li L Luo R. The Oyster Genome Reveals Stress Adaptation and Complexity of Shell Formation. Nature (2012) 490:49–54. doi: 10.1038/nature11413 22992520

[113] Moreira R Pereiro P Canchaya C Posada D Figueras A Novoa B. RNA-Seq in Mytilus Galloprovincialis: Comparative Transcriptomics and Expression Profiles Among Different Tissues. BMC Genomics (2015) 16:1–18. doi: 10.1186/s12864-015-1817-5 26400066 PMC4581086

[114] Li L Qiu L Song L Song X Zhao J Wang L. First Molluscan TNFR Homologue in Zhikong Scallop: Molecular Characterization and Expression Analysis. Fish Shellfish Immunol (2009) 27:625–32. doi: 10.1016/j.fsi.2009.07.009 19632334

[115] De Zoysa M Jung S Lee J. First Molluscan TNF-α Homologue of the TNF Superfamily in Disk Abalone: Molecular Characterization and Expression Analysis. Fish Shellfish Immunol (2009) 26:625–31. doi: 10.1016/j.fsi.2008.10.004 18984056

