# Supplementary material for: Evolution and Potential Function in Molluscs of Neuropeptide and Receptor Homologues of the Insect Allatostatins
Source: Front Endocrinol (Lausanne). 2021 Sep 29;12:725022. doi: 10.3389/fendo.2021.725022 (PMC8514136; doi:10.3389/fendo.2021.725022)
Supplement: Supplementary Figure 1 — Complete deduced sequences of the Mollusca (A) buccalins (B) MIPs and (C) AST-C-like peptide precursors. Each protein precursor encodes multiple small peptides except for AST-C-like where a single mature peptide exists. The predicted mature peptide sequences are highlighted in green. The protease cleavage sites are predicted based on the identification of dibasic residues (KR) and are in yellow. The predicted glycine that forms the C-terminal amide is annotated in blue. The predicted peptides were numbered with letters according to the order in which they appear in the precursors and are aligned in Figure 3 . Sequences that were derived from mantle transcriptomes are annotated with a “*” and those deduced from the genome are indicated with “#”. The orthologue peptide precursors and the localization of the mature peptides in the brachiopod L. anatina, two annelids P. dumerilii and C. teleta and from the insects D. melanogaster and T. castaneum are also represented for comparison. The conserved amino acids that are important for the peptides structure and function are highlighted in bold. [file DataSheet_1.pdf]

Supplementary Figure 1

A) Buccalin/AST-A

MOLLUSCS

<u>BIVALVES</u>

Mytilus galloprovincialis

>VDI48108.1
MVSSVQFRCVASAVIYLILLCNELVCQESTELSKDDELLQYKRKMDMYRFHGSLGKRQD
EQPIPEDQFEKRKIDYARFLGSLGKRANFDDSYPDVERRSKYDRYMFAPSLGKRYVDPQ
ETMEADKRRLDRFSYFGNLGKRGMDKLSYFGSLGKRGLDRLSFFGGLGKRRMDRLSYFG
GLGKRSRDTDERYNNIDSADNHNLYDVNDILPARETRSWYWTNRGTKRLPVSIRGIDKY
SLFGSLGKRSIDQDRLQEILGKRLLSSVES

Mytilus coruscus

>CAC5362126.1
MVSNVQLRCVASAVIYLILLCNELVCQESTELSKDDELLQYKRKMDMYRFHGALGKRQD
EQPIPEDQFEKRKIDYARFLGSLGKRATFDDNYPEVERRSKYDRFMFSPSLGKRYVDPQ
ESTEADKRRLDRFSYFGNLGKRGMDKLSYFGSLGKRGLDRLSFFGGLGKRRLDRLSYFG
GLGKRSGDTDERES

Crassotrea gigas

>XP_011425482.2
MWSTNYATTVFGFFCFVQVFVLTVSQHISSHNDDYTKHLENIKFLNKEAEISPKQPADD
DVDFGNSDTADDLSDLTEEEKRALDRYSFSGSLGKRGLDRYSFYGGLGKRALDRYGFFG
GLGKRALDQYGFAGSLGKRALDRYSFMGGLGKRKLDQYGFAGRLGKRALDRYGFVGTLG
KRKLDQYSFMGNLGKRRLDSHRYFGSLGKRALDRYGFFGGLGKRADTLGNSQENIQGAD
KDEKFEQKRLYPYWYYRQGGSPIYTQTRGIDRFSFAARLGRR

Crassotrea virginica

>XP_022342145.1
MWSYNYAKTVFGCFCLVQVFLCAVSQHIPSHNDDLTNHLESIRLLSKQTENSPKHSDDD
TDNRNSDLTEDFSDLSEEEKRALDPYSFYGGLGKRGLDRYNFFGGLGKRALDKYGFYGS
LGKRALDQNIFSGSLGKRKLDRYGFAGSLGKRALDRYGFTGSLGKRRLDQFGFMGGLGK
RRLDSHRFVGGLGKRALDRYGFFGSLGKRANLSPEDDVDSDGNLLQKRLYPYWYYRQGG
NPIFTQTRGIDRFSFAARLGRR

Mizuhopecten yessoensis

>AXN93469.1
MTNGQHKSQPLTTLFAFLCLVIAVHCSKESLTSRDTKLVNSIQKREADTAAGNAGLDYN
TFHEEPVKRSTNDMHIDDLLLSNSPDKRGRGGNRYGFYGALGKRMDSEEMEKRRMRKMF
FGSLGKRRPSFFAGLGKRDGDETHEETFGDDLDKRGRRNKYFFGSLGKRDLDEGEEDDE
EEDVDKRRMPFFGSLGKRGRSRNFRFYGNLGKRDGTSTDDSTDFDGSDDMEKRRFKQQF
FGTLGKREDDLDLEKRRRYAFASLGKRFDDDDYEENDDMEKRRMKLRPSFYGSLGKRRP
MFYGSLGKRSAPSPDNEMYNEQPEDIHSRRKRSPSSFNLSRALRDGSSYGRARRINRIA
LGRRLIRRTQDFRFFPMLGKRSFDTSDGPEDNGEY

Margaritifera margaritífera
>JADWMO010006559.1
KRRMDRLAFAGALGKRLDELEQAGDGDEEKRRMDRFSYMSNLGKRGTKGRMDFSYFGNL
GKRSRMDRYMFTGALGKRGRMDRYSFIGNLGKRRGRMDRYSFIGNLGKRPSMDRYSFIG
ALGKRGEDLYPYAKSLENQHGMDAYSLDGSLERNAVEDKADIGDTSVDDEDSEFGKRGR
LDMKSFYGQLGKRSKRSMFFPWGGVRYTRDNKPMRGIDKYSFFARLGKREPDYRFLPTL
GKR

Pinctada imbricata
>CM008064.1
HFRTPNSAMLCQLSVCLLYLTSHYTLVTSLQIDDDIQEQTPFEKRRLDRYNFLGTLGKR
QNEEISNERDLEEKRALDYRLFRGSLGKRNKDATDSLDSSEVYPSAGYLDKETEKDGDS
QFQDESNDEGNNVMKGYDLALTEADKRPMDPYMFRGYLGKRMDSRMFSGQLGKRALDRK
MFISQLGKRLDNRMFFGRLGKRMDYRMFSGQLGKRGLDNHMFVGHLGKRLIPVRNSAYY
YRYGSRGRPIYTQTRGMDRFSFAARLGKR

Tegillarca granosa
>JABXWC010000010.1
KRGFDRYGFVGTLGKRQEDEILTPKEQYETDITDSDKREESDDDLTDGENLLTAKRKID
RLMFTGGLGKKRLPYYRFAGTLGKRDDTSTEDEAAEKRGLDRMSYFGTLGKRGQLDMKM
FHGALGKRLDSMYYGRLGKRLNKYFGTLGKRSPLYKSFFGTLGKRGRIDNMYFGHLGKR
DVSSRSRRSLNYGWFNGRPARAIRPIYTQTRGIDRFFSNVGLGKRAPDMRFLPTLGKRF
FIPCE

Magallana hongkongensis
>WFKH01011801.1
KRALDRYSYFGSLGKRGLDRYSFYGGLGKRALDRYGFFGGLGKRALDQYGFAGSLGKRA
LDRYSFMGGLGKRKLDQYGFAGRLGKRALDRYGFVGTLGKRKLDQYSFMGNLGKRRLDS
HRYLGSLGKRALDRYGFFGGLGKR

GASTROPODS
Biomphalaria glabrata
>XP_013065853.1
MLPKNWLHSFVLCLVVLSSCRAYDPDTNEFDGERPEDLVTSDSLDNSEAMDKRKLDRYG
FHMGIGKRDDEEDGDLEDVYEKRRIDPFAFSGGIGKRRLDRFSFAGGIGKRGIDRYGFV
AGIGKRRLDRFGFSGGIGKRGIDRFNFAGGIGKRPIDRFSFAGGIGKRGFDRYGFYGGI
GKRPFDRYAFAGGIGKRPIDKFGFYGGIGKRRLDRFSFAGGIGKRIPDLEEVSAAELSE
AANDVEKRSVPSAKSEKETVKST

Lottia gigantea
>XP_009051897.1
MAARKHELVLVLTSVLCFVSSIVGDPNVPSDSQDNSALTQDDFAKRGMDKFGFAGGVGK
RGLDKFGFTGQLGKRDMDSFGFAGQLGKRGLDQYGFTGQLGKRGLDQYGFTGQLGKRGL
DQYGFTGQLGKRGLDQYGFTGQLGKRGLDQYGFTGQLGKRGLDQYGFTGQLGKRGLDQY
GFAGQLGKRGLDQYGFTGQLGKRGLDQYGFAGQLGKRGLDHYGFAGQLGKRGLDQYGFA
GQLGKRGFDQFGFAGQLGKRGLDHYGFAGQLGKRGLDQLGFTGQLGKRQMDIFGYRGQL
GKRQSIDKYSFLGAGIGKRSVKNTAGIKKDDA

Aplysia californica
>NP_001191649.1
MAHHRGHRHILLYVSLALSLGLALAEDATDPSDDTGSFDDVEAVSEEADLDPYSMSQEL
NKRPNVDPYSYLPSVGKRAFDHYGFTGGLGKRKIDHFGFVGGLGKRQIDPLGFSGGIGK
RYDSFAYSAGLGKRGMDSLAFSGGLGKRGMDSLAFSGGLGKRGMDSLAFSGGLGKRGMD
SLAFSGGLGKRGMDSLAFSGGLGKRGMDSLAFSGGLGKRGMDSFTFAPGLGKRGMDSLA
FAGGLGKRMDGFAFAPGLGKRMDSFAFAPGLGKRGMDSLAFAGGLGKRMDSFAFAPGLG
KRMDSFAFAPGLGKRGLDRYGFVGGLGKRGMDHFAFTGGLGKRDSGEASGDLEEGKRGL
DAYSFTGALGKRGLDRYGFVGGLGKRGMDDFAFSPGLGKKRMDSFMFGSRLGKRGMDRF
SFSGHLGKRKMDQFSFGPGLGKRGFDHYGFTGGIGKRGFDHYGFTGGIGKRQLDPMLFS
GRLGKRSSSEQEEEDVRQVEKRSTTEEQSSKSL

Alviniconcha marisindica
>JABMCL010002885.1
DSYGFTGQLGKRGMDSYGFTGQLGKRGMDSYGFTGQLGKRGMDSYGFTGQLGKRGMDSY
GFTGQLGKRGMDRYGFTGQLGKRGMDSYGFTGQLGKRGMDSYGFTGQLGKRGMDSYGFT
GQLGKRGMDSYGFTGQLGKRGMDNYGFRGQLGKRGMDSYGFTGQLGKRGMDNYAFTGQL
GKRDPD

Conus ventricosus
>CM031604.1
NVTLTHTVLTSILGNGQWTATTSALDWENGLWTATISDSDWEKRTMDRNNFGFGLGKRT
MDRNNFGFGLGKRTMDRNNFGFGLGKRTMDRNNFGFGLGKRTMDRNNFGFGLGKRTMDR
NNFGFGLGKRTMDRNNFGFGLGKRTMDRNNFGFGLGKRTMDRNNFGFGLGKRTMDRNNF
GFGLGKRTMDRNNFGFGLGKRTMDRNNFGFGLGKRTMD

>CM031615.1
LGKRAVDSYNFKFGLGKRQQDPGHPSSVLGKRKVDHNNFSFGLGKRRMDQNNFKFGLGK
RNIDRNSFSFGLGKRGVDRNNFGFGLGKRKMDSTNFGFGLGKRKMDSTNFGFGLGKRKM
DRNNFGFGLGKRKMDSNNFGFGLGKRKMDSNNFGFGLGKRKMDRNNFGFGLGKRKMD

Candidula unifasciata
>CAJHNH020001579.1
MLAKTIPRQLLLLALVIACTQTDARDVTDAADSTDAISDINEPVEIDSVALLAPEDQDS
ETTEGVEKRRIDRYGFYGGIGKRVDRYSFAGGLGKRQLDPFSFAGHLGKRRIDKFGFSG
GIGKRRLDKIGFTGGIGKRRLDRFGFTGQLGKRRIDKFGFAGGIGKRRIDSYGFAGGIG
KRRIDRFGFAGGIGKRRIDRFGFAGGIGKRRIDRYGFAGGIGKRSED

Dracogyra subfuscus
>JAECMU010080661.1
LGKRAIDQYGFMGQLGKRAIDQYGFMGQLGKRFGYGYKRAMDHYGFAPVLGKRGLDQFA
YLSQLGKRGLDHYGFGARLGKRSIDSFGYAAQLGKRGLDHYGFAGQLGKKSADKFGFAA
QLGKKSADHFGFAPQLGKKSADHFGFAPQLGKKSADHFGFAPQLGKKSADSFGFAPQLG
KKSAD

Gigantopelta aegis
>XP_041367891.1
MFLVHRPLGLGLFTITLIAVVVRADDNGYGGDSGADSLGYFHGVDSKQSLNDVSNKRGM
DSYMFSNDQEKQKIDPVMFGARLGKRGMDQFRYISQLGKRAMDQYGFMGQLGKRAIDQY
GFMGQLGKRFGYAYKRAMDHYGFAPVLGKRGLDQFAYLSQLGKRGLDHYGFGARLGKRS

IDSFGYAAQLGKRGLDHYGFAGQLGKKSADKFGFAAQLGKKSADHFGFAPQLGKKSADH
FGFAPQLGKKSADHFGFAPQLGKKSADSFGFAPQLGKKSADHFGFAPQLGKKSADSFGF
APQLGKKSADSFGFAPQLGKKSADHFGFAPQLGKKSADHFGFAPQLGKKSADTFGFAPQ
LGKKSADHFGFAPQLGKKSADHFGFAPQLGKKSADTFGFAPQLGKKSGDKFGFAPQLGK
KSADKFGFGAQLGKKSTDMYGFAPQLGKRGLDNFGYAAQLGKRSVN

Pomacea maculata
>XP_025108540.1
MATRCLAFFIMALTSWACTGRQAVAERWAGSDDNAELVNSSTATTGNSSSSYLRLLRLF
GGNALPELLGNRPADDGGADKRRRTLKLSHPPAMSRTAIWTSASGTWILITSLSAWVRR
SMDPKHFFVGLGKRSMDPHHFFVGLGRRSMDPKHFFVGLGKRSMDPKHFFVGLGRRSMD
PKHFFVGLGKRSIDPRHLFVGLGKRTMDPKHFFVGLGKGSMDPNHLFVGLGKRNLDPHP
FFVGLGKRIMDTRHLFVGLGKRNMDTRHLFVGLGKRNMDSRHLFVGLGKRNMDSRHLFV
GLGKRGFVPASKKQSLDDSKTSFGISKQKRSLREFEQGVYSYPRLPVWRNTGIPASSFR

>XP_025087986.1
MYGEQDQLDKRGVDRNAFFGQLGKSRWINTGFQDSLAKEKWTSPIWNGQQDAQEMLDKR
GMDRNAFYGQLGKRSVGWAKARRHIGRRGLDFSSLYGQLGKRGFDRYSSFGQLGKRGLD
HYGIFGQLGKRGVDGYGFFGQLGKRGFDQYRLFGQLGKRGMDKYGFFGQLGKRGLGHYS
VFGQLGKRDWDAENSEFERSPAKTSTTWS

Haliotis laevigata
>VKKT01005602.1
DTNNNDDKRQTVDKLGFSGTLGKRRFDPILFGGRLGKRGMDRLGFAGTLGKRGMDNFGF
AGQLGKRGMDNLGFAGQLGKRGMDNLGFAGQLGKRRMDNLGFAGQLGKRGMDNLGFAGQ
LGKRGMDHLGFAGTLGKRGMDSLGFAGTLGKRGIDNLGFAGQLGKRAMDKFGFASQLGK
RAMDKFGYASQLGKRGMD

Elysia chlorotica
>RUS89916.1
MDSMSYFGGIGKRPMDSMSYFGGIGKRPMDSMSYFGGIGKRPMDSMSYFGGIGKRPMDS
MSYFGGIGKRPMDSMSYFGGIGKRPMDSMSYFGGIGKRPMDSMSYFGGIGKRPMDSMSY
FGGIGKRPIDSMSYFGGIGKRSMDDMSYFGSIGTNSEHSSDSKNKRRLDYMSLYGGIGK
KSSPVSGEKSVKRQFDAIGLTGGIGKRQIDPLGFSAALGKRDGSGAKIVSDFTGMEKRG
YDKLKYFAGIGKRVVDSLSESSDGDRASIDRDDQPVGIDKRSAGHRVDSKAESDTAKVA
F

CEPHALOPODS
Nautilus pompilius
>JACATO010000118.1
KRGVDSSFFSGGLGKRGVDSSFFSGGLGKRGVDSSFFSGGLGKRGLDSSFFSGGLGKRG
VDSSFFSGGLGKRGVDSSFFSGGLGKRGVDSSFFSGGLGKRGVDSSFFSGGLGKRGVDS
SFFNGGLGKRGVDSSFFSGGLGKRGVDSSFFSGGLGKRGVDSSFFSGGLGKRGVDSSFF
SGGLGKRGVDSSFFSGGLGKRGVDSSFFNGGLGKRGVDSSFFNGGLGKRGVDSSFFSGG
LGKRGVDSSFFNGGLGKRGVDSSFFSGGLGKRGVDSSFFSGGLGKRGVDSSFFSGGLGK
RGVDSSFFSGGLGKRGVDSSFFSGGLGKRGVDSSFFNGGLGKRGVDSSFFSGGLGKRGV
DSSFFSGGLGKRGVDSSFFSGGLGKRGVDSSFFSGGLGKRGVDSSFFNGGLGKR

POLYPLACOPHORS
Acanthopleura granulata

>JABBOT010000025.1
AIILDLGKRNLCSAESILDLGKRNLCSTANILDLGKRNLYPTVIILDLGKRNLYSTAFILD
LGKRNLCSTAIILDLGKRNLCSAAIILDLGKRNLYLTVIILSLGKRNLYSTAIILDLGKRN
LYLIVIILGLGKRNLYSTAIVLDLGKRNLYSTAVILDLGKRNLCSTAIILDLGKRNLCSAA
IILDLGKRNLYLTVIILGLGKRNLYSTAIVLDLGKRNLCSTAIILDLDKRNLYLTAIILGL
GKRNLYSTAIVLDLGKRNLYSTAIILDLGKRNLCSTAIVLDLGKRNLYSTAIVLDLGKRNL
CSTAIILDLGKRNLCSTAIILGLGKRNLYSTAIVLDLGKRNLYSTAIILDLGKRNLCSTAI
ILDLGKRDLCP

BRACHIOPODS

Lingula_anatina

>XP_013384620.1
MEQRTSWSPLFRLIVTLCAVPGLTAADYVSPELLIRGTNGADGQQAFAEKRMDRNMLLM
GLGKRNGLDKHVLFQGLGKRNGLYVTDFGPNPYTFSKRRMSRGMLLAGIGKRMDRNMLF
TGIGKRPMDRAMLIQGLGKRADHDFLLSNLDDGVAAGIERRQDPKMILMGIGKRSALLN
SHPYALMPEEDEFDLNPNFSEDDIQDYDLQERDLDNALRFGGLGKKRRRRSTAVVPSLE
KDVSKRAADDSVLSEDQVVDGSKQKITAKKRGIDRAMLFSGMGKRDFNKRGMDRSMLFS
GVGKRGFDRNMLFTGMGKRSDPKRVMDSQMLLAGLGKRPHDEDEDEDVDHTAIFNELAD
AMDRAESQKNASDDGNINTVDTAEDDRLKIPYNYIYDDDDNMNEKRYSTKMLLTGLGRR
QDDAAQGEFDKRGIDRSMLFSGVGKRRLSKMLLTGLGKRRLDSSMLFSGLGKRGYSKLL
LTGLGKRRLDSSMLFSGLGKRRMDSSMLFSGLGKRRLSNQMLFSGLGRRETKQNVDNSK
RLNDDQSAITSGSEKRTVS

ANNELIDS

Platynereis dumerilii (Conzelmann et al., 2013)

>allatostatin-A-1
KTKNCLESFECGKGSNDNLMISDSFTFPRFKSGEDGSIKLSQLPNVTINDLTNISKYLG
LRKMIDYGLKFHGNGRQTDNTLKFYGPGKRINEILGYSGSGKRTDNGFKLPRRPNNIFK
FSGLVKRTDRARSGIKMRLSGKRVDDISKFSGLGKRDDNIFRFSELGKRVNNALKFSGL
GKRIDDGLRSSGSEKRTNDALEFSGLGKRGNDALQFSGLGKRGNDA

>allatostatin-A-2
RKANNALKFSGLGKRNDALKFSGLGKRANDALKFSGLGKREIDTLKFSELGKRNDALKF
SGLGKRTNDALEFPGSVKRADDHLIFSRLEKKEDDTLNFSGQGKRTEVGLRFSGLGKRN
DDIMNFSGLGKRADDSLGERVNDVEQFSGLEKRANDVMTFSGLGKRMDETLKFSGVGKK
ADDVLAFAELKKRVDNILKFSRIGQREDGGFRFSELEEKPDDILRAGEKNLFRLKKKSD
NGLKFSGLGKSHASKDSRMGKSTDGIMELPGLVKRANEIFRLSGLGKKIGNGLIFTGPE
RHIEEKGTDNSILHRGEKYNSLNLSEFAKYMPQCTKSINNSFLFSKFRKGMIDNAILSV
LSHRIDNGKTTPAPSPRKRRVNSMHKSGPGKEVNQNNVLTVSGSDKFKDNDMMWIHPTE
KINGYHHSRGISAVNQQADLYEPNSKSIPSSLKVGNPKHRKDRSVTLPDWKNHNKTRIP
PGRIFRESKNNLLPHFTPSRLQEIRIEGLNDGIPFKLKQKVVSRNSKKSVRRSKRRSLV
DEISAITNGKRLGHAYHSYVRRLSVDPGISLIGLGK

>allatostatin-A-3
RDPGISGLGKRGNDALQFSGLGKRGNDALQFSGLGKRGNDALQFSGLGKRGNDALEFSG
LGKRGNDALQFSGLGKRGNDALQFSGLGKRANDALKFSGLGKRGNDALQFSGLGKRGNE
ALEFSGLGKRGNDALQFSGLGKRGNDALKFSGLGKRGNDALEFSGLGKRGNDALKFSGL
GKRGNDALQFSGLGKRGNDALKFSGLGKRGNDALKFSGLERKANNALKFSGLGKRNDAL
KFSGLGKRANDPLKFSGLGKREIDTLKFSELGKRNDALKFSGLGKRTNDALEFPGSVKR

Capitella teleta

>ELU12101.1

LDPAVKFAGIGKKDMDPSMKFAG**L**GKREMDSFMKFAG**L**GKKAIDPWMKFAG**L**GKRYSNP
SMKFAG**I**GKKAMDPSMKFMG**L**GKRYSNPSMKFAG**L**GKRYSNPSMKFSG**I**GKKAMDTSMK
FAG**L**GKRYSNPSMKFAG**L**GKKAMDPSMKFAG**I**GKKSMDPSMKFAG**L**GKRYSNPSMQFAG
IGKRSTDRMMLAG**I**GKRISIDPGIKYFG**L**GKRSFDPTYSHIG**L**GKRSE

INSECTS

Drosophila melanogaster

>Q9VC44

MNSLHAHLLLLAVCCVGYIASSPVIGQDQRSGDSDADVLLAADEMADNGGDNIDKRVER
YAFG**L**GRRAYMYTNGGPGMKRLPVYNFG**L**GKRSRPYSFG**L**GKRSDYDYDQDNEIDYRVP
PANYLAAERAVRPGRQNKRTTRPQPFNFG**L**GRR

B) MIP/AST-B

MOLLUSCS

BIVALVES

Mytilus galloprovincialis
>VDI26375.1
MNLNIQNSLISCVTFLSVFVICNSAETLSKDDSNNAELSSIIGQNLIDTANQDIDKRKW
GSVASWGKRGWPLYSGKRWSVLHTWGKRASPDSDDINKRKWSGFTSWGKRALKDYLNND
DDKRKWSGFTSWGKRDLPDYENVDKRKWSGFTSWGKRSDSDDIENDKRKWASFNSWGKR
SPSSDDTELDTDMDKRRWNQVGVWGKRSEPEKRRWSSVSAWGKRGWNNFQSWGKRPWSS
FKSWGKRNPWHSLSTWGKRSVPG

Mytilus coruscus
>CAC5388310.1
MTPGKSVLIRIINRMKLNIQNSLISCVTFLSIYVICNSAETLSKDDSNSDELSSIIGQN
LIDTANQDIDKRKWSSVASWGKRGWPLYSGKRWSVLHTWGKRASPDSDDIDKRKWSGFT
SWGKRAVKDYLNNEDKRKWSGFTSWGKRDLPDFENVDKRKWSGFTSWGKRSDSDDIEND
KRKWSSFNTWGKRTPLSDDSELDNDMDKREWNQVGVWGKRSEPEKRRWSSVSAWGKRGW
NNFQSWGKRPWSSFKSWGKRNPWHSLSTWGKRSAPVESDQDATYTAQAV

Crassostrea gigas
>XP_011417556.2
MLCHLEQLLLSCIVLCLVKVCASLKAQDEASVNDHDIVRRQAMGHSFGDELEGFDLDPK
RVNNWNQFPAWGKRLSKRRWSSLGAWGKRSWLDRLISANNNWGKRWKSMSNSWGKRQAP
SEFDGLSDDYINIKKRSVDSKSSHNSRNKRSIPTELSPEQNEEKRRWSSLSAWGKRSDD
DEKRRWSSLSAWGKRSNPEAIDDNDSDNISKRKWSSFSSWGKRGDPVDLSKRLYSYWQN
RLMTNNPWMERRGWNAFSSWGKRSMD

Crassostrea virginica
>XP_022314361.1
MNCDAQQLFLSCVILCLVKVCASLGTQDEGSISDHDVVKRQAIGNSYGDELEGFDLDPK
RDNNWNQFPAWGKRPSKRRWSSLGAWGKRSWLDRLISANNNWGKRWNSMSNSWGKRQAP
MEYDGLSDDYINLKKRSVNSKLNNITRKKRSTSPEASPDESEEKRRWSSLSAWGKRSDD
AEKRRWSSLSAWGKRSYDDLFDSKNSDDVSKRKWSSFSSWGKRGFPAEISKRLNDYWRQ
RLMFNTPWIERRGWNALTTWGKRSMD

Mizuhopecten yessoensis
>AXN93470.1
MKFQMSITRLLCLTLLVLKTAYCMAKSLDGIDKTTGAVDASREKRGVLNSDSKEEALSK
FINTLDASPADESEVAKRGWQKFNSWGKRVLVNLSPWGKRRWARLQSWGKRSIDPSAID
ETGKDDIDSEITPEQALLYNEGDKRGWKDMGTWGKRDPTDELTDAGLEDVDKRKWTGYA
SWGKRDDENVSDDLLPKRKWNQLSAWGKRAEALTADQLEAIKRKWSSMASWGKRSNWSG
FNSWGKRNPWSNLATWGKRSKWSGFNSWGKRNAADDLEQ

Margaritifera margaritífera
>JADWMO010008838.1
RAWSQFAAWGKRDEGGKRKWSQFASWGKRNKEENGDDKRKWSQFTSWGKRDMDENMLSDKR
KWSQFASWGKRDEDEKAAEKRKWSQFVPWGKRDDGDKRKWSQFVPWGKRDDDFLDADKRKW

SQFASWGKRDLDSMDADKRKWSQFAPWGKRDDNSIDEEKRKWSQFASWGKRDLDSMDADKR
KWSQFASWGKRTGENMEDSEKDKWSNFKSSGKRNWGGLAVWGKRSTDD

Pinctada imbricata
>CM008064.1
RKWPGTNVWGKRKWMNVWGKRKWASMGTWGKRSDPLIDNGLENIHKRDTEHSFPLKRKW
NQFVTWGKRSMPIQRKWASVSLWGKRSQDNYNEPEKRKWSALTSWGKRDDVGDINSDKN
KRKWAMSAWGKRTNPIDGDNDMNKKWALASWGKRNDIYNTPRLQDNDAKSDNTVDKRKW
SNFSSWGKRMSIQNWRKRPWLWAKRRRGWSAMSSWGKRSSLD

Sinonovacula constricta
>RXXH01000349.1
RKWSSFASWGKRAGWKSNFAAWGKRKWGNFAVWGKRNAGDSTPRYLYDIAQPLEDKRAW
QFASWGKRTSAIPEIDQKRNKEEFQDALNVDVDKRKWSQFASWGKRDIGDDAEIDKRKW
SQFAAWGKRNGLNDLDKRKWSQFASWGKRSDDIDSENDLQEADKRKWSQFASWGKRFND
MESPEKRKWSQFASWGKRDDDDDLSYSSADKRKWAQFASWGKRGDLDDEQSLPKRRWSQ
FASWGKRLRDARLAALSSWAKRRWTGLPAWGKRSAD

Tegillarca granosa
>JABXWC010000010.1
MRTDIKCLGLFLLTILSLSQVLCEDSQELTNSDSLENSINQHIDTSPQNSYSKREWSKF
ASWGKRGWPQFASWGKRVFDSDDLMDKRKWSSLNSWGKRAWSSLSSWGKRDMNTDEPVD
KRKWSSFSSWGKRGYDRPIVYGQWSAEHLEDDDNMPDKRKWSKFASWGKRDPTDKRGWS
KFASWGKRDDSPIDTDKRKWNFNSWGKRSSWNGLQAWGKRSKWSALNSWGKRDGQLNNL
HDYLF

Ruditapes philippinarum
>CM018529.1
RLNSEGTTKLTAEGKRGWDGNFAVWGKRDYDAFSENARDNSLSSAQLDPEKRKWSKFAS
WGKREIPLDSNTGWIDQDIDNKRDLEVMTEPLKIDDSEWPKHSTDKRRWQALNTWGKRT
IANSLDEDSKRGWSGFTSWGKRDSEADQLNNIDKRKWQKFASWGKRDDEQLIGDADKRK
WSKFTSWGKRNYEPILAELKKRKWAKFTSWGKRSEINDDLEAEKRKWAKFTSWGKRDND
VDTDDELDTPDKRKWNRLAVWGKRSNDESNFVDKRNWAKFASWGKRPRNPQAWLALNTW
GKRRWTGLTTWGKRSGEGFNAET

Magallana hongkongensis
>WFKH01011801.1
MLCHLEQLLLPCIVLCLVKVCASLKAQDETSVNDHDIVRRQAMGHSFGDELEGLDLDPK
RVNNWNQFPAWGKRLSKRRWSSLGAWGKRSWLDRLISANNNWGKRWKSMSNSWGKRQAP
SEFDGLSDDYINIKKRSVDSKSSHNPRNKRSIPTELSPEQNEEKRRWSSLSAWGKRSDD
DEKRRWSSLSAWGKRSNPDAIDINDSDDISKRKWSSFSSWGKRGYPVDLSKRLYSYWQN
RLMTNSPWMERRGWNALSSWGKR

<u>GASTROPODS</u>

Aplysia californica
>NP_001191611.1
MTLHLASPFILLFTIAYSLTSAVQGLEPLPAASLSDSPASGADVPPLPSSAATNAAVDK
EWLRQKLEEGQFLPQQDKRWGGINSWMTHRLGGPSERDSSQDSLDKQLLVNNVQNYDDS
SKRKWSKFSSWGKRDASEETPEGGEGEDGLGAVKKWKNMAVWGKRAEDGLDKRWKQMAT
WGKREDGDVLGLGTDKRWKQMASWGKRLDDSDRDKKWKQMSVWGKREDNGEPLDKKWKE

MSVWGKRDTLDDPEKRWKQMAVWGKRQGLDDRNDKRWKQMATWGKRNSSENYDKRWKQM
SVWGKRDGDGDLDKRWKQMSVWGKRDGDGDLDKRWKQMSVWGKRNGDGDLDKRWKQMSV
WGKRDGDEDVEKRWKQMSVWGKRDGDADLDKRWKQMSVWGKRDEDGNLDKRWKQMSVWG
KRDEDGNLDKRWKQMSVWGKRDGDDNLDKRWKQMSVWEREREREWV

Lottia gigantea

>XP_009051899.1
MDLKTILCLIIYSLLLQISHAEEQLANDIELSNSLNPVDKRAWKSSYLNTWGKRWNPRY
NLRGYQRMPIWAKRWTNSGLITWGKRSADTEIPIHKRKWNQFITWGKRSGVPSIVKRSV
GDELVPWGKNKDTLPELNTSSDNLDNKLIDLETTPSTDKLSLDEKRASDKGWNGFTTWG
KRANKDWSSLSTWGKRGQNKDWSSLTTWGKRGHDRDWNSLTTWGKRANKDWSSLSTWGK
RARENDWSALSTWGKRANNKDWASLTTWGKRANDRDWNSLTTWGKRAKGNNWSGLTTWG
KRANKDWNSLTTWGKRANKDWSGLTTWGKRGNKDWSGLTTWGKRGNKDWSGLTTWGKRG
NKDWSGLTTWGKRGNKDWSGLTTWGKRGKNDWSGLTTWGKRGNKDWSGLTTWGKRGNND
WSGLTTWGKRGKNDWSGLTTWGKRSPDATSEDSGELSTLDKKDIKGWNGLTTWGKRFAG
DKNKWSSLTTWGKRDDNNKQDDKKWAQLSTWGKRSPEDAAELWKIYDSNGDGIMDKEEM
VSFLRSAASQKDSQQDEKS

Alviniconcha marisindica

>JABMCL010002007.1
RTPTKSGAMLLPWGKREDPDAHKKWSNVATWGKREDPDAHKKWSNVATWGKRNALHADK
KWSNLATWGKRDALQADKKWSNLATWGKREDPDVHKKWSNVATWGKREDSDAHKKWSNL
ATWGKRDDLQADKKWSNLATWGKRDDLQADKKWSNLATWGKRDDLQADKKWSNLATWGK
RDDLQADKKWSNLATWGKRDHMDLPNADKKWSSVATWGKREQPDSPDAEKKWSNLATWG
KRHDMQASPDADKRWNSMASWGKRSRDWGSVATWGKRGNRDQWGAMATWGKRGSDGLGD
ADRVMPPKEALFQLFDHDG

Conus ventricosus

>CM031614.1
KREWNQFVTWGKRDGSHHLAKRWAHAGGSQQVKDEGNALADNSANGETSDESNDASSEQ
LDQNDDSGSKDSDGSSSAPGEDDQNSDKDKHRQGTEEELGQAKDSVVKRWNSLATWGKR
EDSDDKKWSNMAVWGKRSPQDPSTPPKKWSLVNTWGKRWSNMATWGKRDSQPADKKWGN
VAVWGKRGDDVNKRWNNMAAWGKRDHPDPKRWASLTTWGKRNDADAEGQRADKKWSSLS
TWGKRGQMDDTDMDKKWSNMAAWGKRQHQQSLIPDQKRWNALTSWGKRDSRNWNVMSAW
GKRGLED

Candidula unifasciata

>CAJHNH020002065.1
KKWKEMSVWGKRDDSMDKKWKEMSVWGKRDAEMDKKWKEMSVWGKRNDEMDKKWRDMAV
WGKRDQEVPNVNWNYIAASGRHSLTPDLYKKWKEMAVWGKREDGDLDKKWKEMSVWGKR
NDELEKKWKEMAVWGKRNNELDKKWKEMSVWGKRDYDKKWKEMSVWGKRDDALDKKWKE
MSVWGKRDGDYDKKWKEMSVWGKRDDALDKKWKEMSVWGKRLTLPDLDKKWRDMAVWGK
RNGEHFDNNNDNNNVDKDGAWEKRTLRHVRNWRDMDMWGKRPSWSKTGFTSWGKRSQDV

>CAJHNH020003852.1
CLTCYILLVFVSHAASVSTEKGDPLSWYIAHDLTGSDDLNLATRFNTQTVRNDTDIFRL
PGSQRQPNLPLDVAPVAGLQIQGFPDGNSWTSRNSRWSGLSSWGQRTPSAAGWGDEILD
NTPSQTRNKRKWSHFSSWGKRSLHNTFGDGTDKRWKEMPVWGKRDGADKTKKWKEMTVW
GKRNDIEGIKKWKEMATWGKRNEIDNKKKWKEMTVWGKRDFIDNLKKWKEMSVWGKRND
IDNTKKWKEMSVWGKRNDVDNIKKWKEMSVWGKRNDVDNIKKWKEMSVWGKRNDVDNIQ
KWKEMSVWGKRNYLDNTKPWKEMSVWGKRNWDMNRSEMNLWRQNEQARLGKKWKSLSAW

GKRDSMTDSDTDISSLGNRDRDHIGFVNNVSTRDPSEGTNLDKKWREMTVWGKRSIQSP
EARLPTQLPLWRTLRRVRNWRDMDVWGKRPSWSKTGFTSWGKRSAD

Dracogyra subfuscus
>JAECMU010090506.1
MDLMNIICLVLSTFTIHLSIADDSTSLQSAAKPSANVHSPLASDGSALDKRKWSNFNTW
GKRGWMKFGTWGKRWHKPLSPWGKKWSSFATWGKRSGDESDSQNLQNEQKRTWNQFITW
GKRNWKALATWGKRSADEHTNWNTMPIDEKRKWNAMATWGKRSADEHTNRNTMPIDDKR
KWNAMATWGKRSADEHTNRNTMPIDEKRKWNALATWGKRSSDEQTNWNTMSTDEKRKWN
KFASWGKRSD

Gigantopelta aegis
>JAEHGF010000009.1
MDLTNIICLVLSTFTIHFSIADDSTSLQSAAKPSADVHSPLASDGGALDKRKWSNFNTW
GKRGWMKFGTWGKRWHKPLSPWGKKWSSFATWGKRSGDDSDSQNLQNEQKRTWNQFITW
GKRNWKALATWGKRSADEHTNWNTMPTDEKRKWNALATWGKRSADEHTNWNTMSTDEKR
KWNALATWGKRSADEHTNWNTMSTDEKRKWNALATWGKRSADEKTDWNTMSTDEKRKWN
KFASWGKRSD

Pomacea maculata
>SRHC01003577.1
MEILNLACFLLAVCAVLHLSLADEALSSASDQKPEAPSQTDDVAILQDGESLEKRWPHDQW
AAANNWRKKWMAMNTWGKRWTNQFATWGKRNWNERPFATWGKRRPDWNFATWGKRAWNDRQ
FATWGKRAWNNRQFATWGKRAGDSLPEGSSPNDVADVDFPEKKAWNQFVTWGKRVPSDDVN
KRWNTMATWGKRSNSGDDISNHEDAISNGSGLDSEQQHHQQLQQQGALSSDAAADKRWNS
FATWGKKWNTMSTWGKRDNNAEDADSLDKRWRSMATWGKRDSADQGEARYKRSRLGPWGKR
VRAEGWSVMPTWGKRGRGQWGVLSTWGKRAAGDRPG

Haliotis laevigata
>VKKT01000261.1
SVPEPNCPMDLKSFFCLILPTLATLQLSLAEDPSLVEGVKPVSEAGQRSLQDSDSAADK
RQWSNFHSWGKRWGNFGTWGKKWASSDFPAWGKRWAGSSFTSWGKRNASPEEQKRNWNQ
FITWGKRNWHALTSWGKRSQDKTEKKWNTLPTWGKRAGWDNGFASWGKRDEVD

Elysia chlorotica
>RUS72157.1
MELRLLSSSTVLAVQIIIFIKLHVLVTSGHSVSGDQAQLASGSSQLVPSKDIAKEILAL
DPVQNVFSSVDELQGEPLQGQLSLDPAQLARWSSKLRSWAEKPSHGLKDTISESDNAPL
DKRWREFNSFGKRSDGERDRDVFGVEGVDNYPGEWSKAAKDDWKGIEIPEKKWKEMSVW
GKRDEIPEKKWKEMSVWGKRDEIPEKKWKEMSVWGKRDEIPEKKWKEMSVWGKRDEIPE
KKWKEMSVWGKRDEIPEKKWKEMSVWGKRDETPDKKWKEMSVWGKRSGVAEKKWKEMSV
WGKRDDNLVNKNWGKRSTALELSRIWKSMPVWDKRSFQLKPGHTFRRIQNMPNLENAWR
KRPSWRTPGLTTWGKRASPAGLSAQEQRFQRQFHKVQDLLFGQEGQVQEHPQEDSAADK
GPTAAQKRMEG

CEPHALOPODS
Octopus bimaculoides
>XP_014775853.1
MATFLNFLLFVTVLALTKFSTLSAELNELSNKQSAIKVARSMMPAKFLESDLEAWDEKI
GDQRNWEDRLNMKRNWDELSSWGKRSAFDNNAIDNEFGNSLRRALNSHNLKLSSLLNGA

ELQKRWDSLQAWGKRNANGNEAAYGKNVKRDHMVKASDGTWKKVGSADGDKRGNGWDEM
NGWGKRNAVQHKIRSRNWDSLQAWGKRESPSDDSALYENNQRQKRSTGKIL

Nautilus pompilius
>JACATO010000978.1
MNSIFANIAVVFLVVSQSLSLNAEDKRDLNDPGDRLKPSVSDLSPDEDGPVREADVKRQWS
QFGTWGKRWSHFPTWGKRWDKFTTWGKKWQNPGSVLTALGKRRADDDVDKRQWKQFITWGK
RGDTKRDWSSFSSWGKRNAWNNFSTWGKRANIDDGILVERVMNLFDTNGKQS

Sepia pharaonis
>CAHIKZ030000343.1
INMASIFSHFLVIMMLALTQTRRLIAEEKNDIGSSKNLKPVSVVKSENLKSWSKRSTTN
GKALNAIRQAVARGAFPAPANALDSSDERYWNLMLLWLKENGYPSTTANAWGRRTGLRS
RVAREIDGTDEELPEKKNRLDTWNSMNTWGKRSPNTWDSMAAWGKRNPNTWDSMAAWGK
RNPNTWDSMAAWGKRNGDTWDSMSAWGKRNPDTWDSMSAWGKRGADTWDSMSAWGKRGA
DTWDSMSAWGKRGADTWDSMSAWGKRGADTWDSMSAWGKRNGDSKEKRDWDSLQSWGKR
VNAKNKKDWDSLAAWGKR

Architeuthis dux
>VCCN01004164.1
INMASIFSHFLVIVMLALTQSQTSTAEEKRDLGNTKNLKPVSVIKTDNLKLWNKRSTSN
GNALNAIRQAVARGVIPAPSNPLDNTDERYWGFLQLWLRDNGYPLTAANAWGRRMGLKS
RVARETDGTDEELPEKKDRLDTWNSMNTWGKRSPSTWDSMAAWGKRNPNTWDSMAAWGK
RNGDTWDSMSAWGKRNPDTWDSMSAWGKRGADTWDSMSAWGKRGADTWDSMSAWGKRAG
DTWDSMSAWGKRAGDTWDSMSAWGKRNGDSGAKRDWDSLHAWGKRVKDKKDWDSLAAWG
KR

<u>POLYPLACOPHORS</u>
Acanthopleura granulata
>JABBOT010000001.11
AVSDENIDRDTLDIKNLDEIEKPSSSKMEEKRKWADYATWGKRKWEGVPAWGKRWSEGL
TTWGKRDQAKRQWNQFVTWGKRSSAGDKRKDWSSFSSWGKRGKWSGFNTWGKRDANTD

BRACHIOPODS
Lingula_anatina
>XP_013414390.1
MAAITADSLTITFFLTLMVLSSPLVHCVINSQMLRDAQNLDQAPYGRYIEEDIVIPNDS
QEEENSHRYQSQAVSLKRLEYLFNKLENNIMRNEPLKRQWSKSMTMWGKRSPYFYSEDM
EDPHALRLRRRSLEKTDDNNKTKEKTDFQLTNNVIRSWKSGGMKLWGKRPFDKEMEESR
DADLKKLGENMDWKQKERDLKRSWSGNAMKLWGKRDNTFDDQHDKKSWSSKNMKLWGKR
EDDKRSWNSKSLKVWGKRDVDQDEEENNKRSWSSKNMKLWGKRNEQKDGLEYEKRPWSS
KSMKLWGKRDNSVEADINEKRQWNSNGMKVWGKRMDGEIIDELSNKRKWNSKEMKLWGK
RDSVQHNALNEKRGWNAKGMKLWGKRSENDEKRPWNGKGMKLWGKRDSSGYESESNNYP
YLYRKHSIDVDSKRSWKSGGIKLWGKRDMVDDDVDLVSNTAGGVKRPWNSGMKLWGKKR
ASDSLRDLDSAKSDSWIQDGKRSWIGKNPWATKIGFDPSKRWRSMKLWGKRDSGQSQHD
YEDAFNKWNSMKLWGKDLTSLIKAEVGNNPNTKTLSNEFEEKVFDHGVDNETNTETGSS
PLNKEISNEHVTTRAHTLSKRSGWMPREGLRNMWG

ANNELIDS
Platynereis dumerilii

>AFV92893.1
MDRVTITCFSLCLASVLIPLVHSEENVDLDEDKRAWMKNNIAWGKRGWKQGASYSWGKR
DSEGDGLMSDEEKRAWNKNNMRVWGKRSDEDDKRGWKDSSMRVWGKRAGEDDNNKRWGK
NNLRVWGKRADDLEVLEDKRAWGDNNMRVWGKRSDLEDDKRAWNKNSMRVWGKRDMEED
EDNKRAWKGQSARVWGKRADEDDKRGWNGNSMRVWGKRGWHGNGVRQWGKRLHLDDVEP
ILDDEESKRAWAKNNMRVWGKRSTDNVRNMKAVVAEPAEVAADAESAKSS

Capitella teleta
>ELU01469.1
MASCRLLLTVITLVICSLVVLADDPETEEQAQDLVPHDMDQDLMDKRKWGSNSMRVWGK
RDGDDEMEMDGGAEKRKWGGNNNMRVWGKRKWGANSMRVWGKRSELPEEEKRKWGGSNT
MRTWGKRADDNEEDELAKRKWGSNSMRVWGKRADDNKRKWGSNSMRVWGKRADDDMDES
KRGWKNNNMRVWGKRADDEIDEDKRKWGSNSMRVWGKRSADDDAELAAAVPHAIVKRSL
DSEEFTDDMEKRRWGGNDMRVWGKRRSRADGPKRSWKTNVMRVWGKRGWADNNMRVWGK
RADEGAEKRAWVGDKSLSWGKRSDNEVIRNLLAEQVMMISIISPTKYLRDVAICVGGVL
GGFSRDPLSE

INSECTS

Drosophila melanogaster
>Q9VVF7
MAHTKTRRTYGFLMVLLILGSACGNLVASGSAGSPPSNEPGGGGLSEQVVLDQLSESDL
YGNNKRAWQSLQSSWGKRSSSGDVSDPDIYMTGHFVPLVITDGTNTIDWDTFERLASGQ
SAQQQQQQPLQQQSQSGEDFDDLAGEPDVEKRAWKSMNVAWGKRRQAQGWNKFRGAWGK
REPTWNNLKGMWGKRDQWQKLHGGWGKRSQLPSN

Tribolium castaneum
>NP_001137202.1
MMSFAAAIMRDAVAPVLGAVLLTCYSLQATLALSDETPLKSSNDNPQIEDEMSKRDWNK
DLHIWGKRGWNNLHEGWGRKRSVPAWEEQQEKRAWQSLQSGWGKRFAPEDEYAIRQLAA
MLDSQYDDYNPEIETNDDEKRNWGQFHGGWGKRSKWDNFRGSWGKREPAWSNLKGIWGK
RSGEK

C) AST-C-like

MOLLUSCS

BIVALVES

Mytilus galloprovincialis#
>UYJE01007806.1
MNFSKSLSIIVKFLTVLLVLVLSTHAQMDVDEKALSTQAVEDYRFMKSILEVFYKSQLE
QFQEQEAFIQQQLDILHDQREAVRTKKRSHVQ**C**LVNVVA**C**YKKRR

Mytilus coruscus*
>c79189_g2c79189_g2_i1
MNFSKSLSIIVKFFTVLLVLVLSTHAQMDVDDKALSTQAVEDYRFMKSILEVFYKSQLE
QLQEQEAFIQQQLDILHDQREAVRTKKRSHVQ**C**LVNVVA**C**YKKRR

Crassostrea gigas
>XP_011412814.1
MELTQSVFVLKLYAAVVAVLLVAEVHAQPQKFSTEIQQTGDESSTDLNLFKMALREAYN
RELEFYEQQEAQIVKQLAALENDRNQIRERKRSHIR**C**LVNVIA**C**YRKK

Crassostrea virginica
>XP_022344895.1
MEISNSVFVLKVYALLVAVLLAAEVHTQPQKFNTEIQQTSDDSSSELNLFKMALREAYN
RELEFYETQEAQIMKQIAALENDRSHLRERKRSHIQ**C**LVNVIA**C**YRKK

Mizuhopecten yessoensis
>XP_021356393.1
MIGRDVYRLCFMLGTMFVCTLLITTAKAQSADENLSSALQAGDGEMMDMGGRGGLQDAA
YAYRQLALIQDEEDTLLRMMDNLKTKMEHVKVRKRGHIQ**C**LVNLVA**C**YGKRK

Margaritifera margaritifera
>JADWMO010001387.1
QRQDITERKRSHVH**C**LVNLIA**C**YRKRK

Pinctada imbricata
>CM008067.1
LESQKKSIQARKRSHVQ**C**LVNVIA**C**YRKRK

Tegillarca granosa
>JABXWC010000007.1
LESQTHSVDAQKRSHVQ**C**LVNLIA**C**YKRRK

>JABXWC010000007.1
LESQTHSVDAQKRSHVQ**C**LVNLIA**C**YKRRK

Ruditapes philippinarum
>CM018530.1
LYKERENIRTMKRGHTQ**C**FLNLVS**C**YRKRK

Magallana hongkongensis

>WFKH01010931.1
LENDRNQIRERKRSHIRCLVNVIACYRKK

GASTROPODS

Aplysia californica

>XP_005112794.1
MSVSVRTWRAVNTCLLLTLLTLWADVLVVRAAVIPVSSPEPMEEASALQLLPGKIGRAS
LLREMERQLMILQAAEENIVSGLQELEEERRVLSGRKRSHYSSMCMFNVVACYRKRK

Alviniconcha marisindica

>JABMCL010002252.1
EALKAKRQAISQRKRSHYMCLVNLIACYK

Conus ventricosus

>CM031611.1
EALKAQRQAVSQRKRSHYMCMVNIVACYK

Dracogyra subfuscus

>JAECMU010248346.1
LQAQKSQISQRKRSHYMCLVNLITCYRKRK

Gigantopelta aegis

>CM027804.1
LQAQKSQISQRKRSHYMCLVNLITCYRKRK

Pomacea maculata

>SRHC01001246.1
LKAKRQAISERKRSHYMCLVNLIACYRK

Haliotis laevigata

>VKKT01000229.1
LQHQRQEITQRKRSHYMCLVNLITCYRKRK

CEPHALOPODS

Nautilus pompilius

>JACATO010002127.1
LQQRKQEMTKRKRNHVQCMVNVVSCYRKRK

POLYPLACOPHORS

Acanthopleura granulata

>JABBOT010000080.1
QKRQHVACLVHVLSCYKKRR

BRACHIOPOD

Lingula_anatina

>XP_013393587.1

MSCSGRRCVFLRRQDWALALCAVVMSSMVLLARAAPATVETEPGYGSVIEVTDEEFRPA
GTAVQVPDYFTSGRQAYLNNFKERALVASKIRELEKEIDELDKLGSKLADQISAVQRKR
ANLQFKKRQRIA**C**QVWIVS**C**FGK

ANNELIDS

Platynereis dumerilii

>AHB62362.1
MVDFTAHTLAIVLIINCICIAFAEPAPSELGSVGDYENEHYKSLQGGMLERRLREDIER
EMTDVKKLEHQLMGHLNLIQEKKRQLEIKKRQPVQ**C**LVNIVS**C**WKRK

INSECTS

Drosophila melanogaster

>Q95NV8-AST-C
MMKFVQILLCYGLLLTLFFALSEARPSGAETGPDSDGLDGQDAEDVRGAYGGGYDMPAQ
AIYPNIPMDRLQMLFAQYRPTYSAYLRSPTYGNVNELYRLPESKRQVRYRQ**C**YFNPIS**C**
FRK

>Q9VKK4-AST-CC
MHQPPGRQTARRRRSCTSLAGKEGTPLCRTYHLPAMLIILLVLIQNFELHMCRQLMVYP
GADKRSPDKLLTIGGSAAGEVTLPEANTPADDKRAGGSRSAPSQPEEIFSAPADEGYDE
YPMVVPKRAALLLDRLMVALHHALEQERSEQRIGEFFGDRNILSGKFGDSHNGMEHHQA
REDGMYSDDDAGTLLDYDFKDLNQINRATGETRRAGADRSGTSTHSGSPAGSRRIQPSG
SGGGRAYWR**C**YFNAVS**C**F

Tribolium castaneum

>NP_001137205.1-AST-C
MAAQLPRYLTKTLFIFLIATLVVANARPNHFGDASQVVGEPADGNNLLDSRLKPWELEM
LVQRLSEISSQTGGDFAWDKSIRLPEAKRQSRYRQ**C**YFNPIS**C**FRK

>XP_001810067.1-AST-CC
MNRILMVVLESFLVAVLFEMKTDGFLIDRRSAASERNSDDYPDYQLGVKYDEYPMIVPK
KRTALLVDRLMVALQQAIEEEEAANRVDGPPLTNSFQLSPEEVRKMDLQRRGHGSMSGQ
QKGRVYWR**C**YFNAVT**C**F
