# Supplementary material for: Evolution and Potential Function in Molluscs of Neuropeptide and Receptor Homologues of the Insect Allatostatins
Source: Front Endocrinol (Lausanne). 2021 Sep 29;12:725022. doi: 10.3389/fendo.2021.725022 (PMC8514136; doi:10.3389/fendo.2021.725022)
Supplement: Supplementary Figure 2 — Deduced amino acid sequences of previously designated Mollusca buccalin-like precursors. The peptide precursors are now known to be members of the LASGLI/V-amide peptide family. Each precursor protein contains multiple small peptides. The predicted mature peptide sequences are highlighted in green. The protease cleavage sites are predicted based on the identification of dibasic residues (KR, RK) and are in yellow. The predicted glycine that forms the C-terminal amide are annotated in blue. [file DataSheet_2.pdf]

**Supplementary Figure 2**

**Mytilus galloprovincialis**
>VDH93971.1
MSQFDFIQMGYISFFILCLVLTASTESNNDVGDDSEFMDEDIIDTGDLDKRLFERLASG
LVGKRPFDRLASGLVGKRPFDRLASGLVGKREFDRLASGLVGKRPFDRLASGLVGKRPF
DRLASGLVGKRPFDRLASGLVGKRPFDRLASGLVGKRPFDRLASGLVGKRYMDPLASSL
VGKRDSQ

**Mytilus coruscus**
>CAC5386940.1
MSQIEYIQMGYISFLVLCLVLTASTESNNDVGDNSEFMDQDISDIDTGDIDKRLFERLA
SGLVGKRPFDRLASGLVGKRPFDRLASGLVGKREFDRLASGLVGKRPFDRLASGLVGKR
PFDRLASGLVGKRPFDRLASGLVGKRPFDRLASGLVGKRPFDRLASGLVGKRYMDPLAS
SLVGKRDSQ

**Crassotrea gigas**
>XP_011415970.2
MATTKSNAVLFTTIIYVVYTIGPIASQKSFRYDENLYQDQDETLVEKRQFDRLASGLIG
KRRLDSVASGLVGKRRLDTIASGLVGKRRLDSIASGLVGKRRLDTIASGLVGKRRLDSI
ASGLVGKRRLDSIASGLVGKRRLDSIADGLVGKRKMDYHSNTYPHFPAAEKRMMDSLAS
GLVGKRMMDSLASGLVGKRTMDSLASGLVGKRTLDSLASGLVGKRQSFPDFSKGGNEED

**Crassotrea virginica**
>XP_022340818.1
MAPSKSRHILLATFIYLMHNIAPIASQADFKYDDNSFQELEGTQAEKRQFDRLASGLIG
KRHLDSIASGLVGKRHLDSIASGLVGKRRLDSTASGLVGKRRLDSIASGLVGKRRLDSI
ASGLVGKRRLDTIASGLVGKRRLDSIASGLVGKREMEDHLNSYHKVPGFEKRMMDSLAS
GLVGKRMMDSLASGLVGKRTMDSLASGLVGKRTMDSLASGLIGKRALDSIASGLVGKRQ
SFLDFSNSGAGNEK

**Mizuhopecten yessoensis**
>XP_021372073.1
MTASTLRSNMDSLLSTLGKCTLVILYITGGWCGTQDVSHDDGSLTQEDKRGLDSIANSL
DLASGLEEKRMMDPLANGLVGKRYMDSLASGLIGKRFYDDSASDLIEKRYIGGIANGLI
GKRYMGSIANGLIGKRYMGSIANGLIGKRYMGSIANGLIGKRYIGGIANGLIGKRYIGG
IANGLIGKRYMGSIANGLIGKRFMSSIANGLIGKRYVDNIASDLIGKREDNGEMHEEKR
YIDSLANGLIGKRSDDEEGYDNERSVVDKRYIDNLASGLIGKRSNSYRHHDLQALLDKR
PFGQLANGLIGKRSGDD

**Biomphalaria glabrata**
>XP_013071848.1
MEGTNTGLRRRNLWAVETNSSLPGRVDMKRPFDEIGSGLIGKRPFDEIGSGLIGKRPFD
EIGSGLIGKRPFDEIGSGLIGKRPFDEIGSGLIGKRPFDEIGSGLIGKRPFDEIGSGLI
GKRPFDEIGSGLIGKRPFDEIGSGLIGKRPFNEIGSGLIGKRPFDEIGSGLIGKRPFDE
IGSGLIGKRPFDEIGSGLIGKRPFDEIGSGLIGKRAF

**Aplysia californica**
>XP_012945643.1
MTSPRRLTSFLFLSLAVCATIALSASETAATDDEESGEEGHVEKRSIWARRNRLGFNSF
RRYFNKRPFDELASGLVGKRSVDAKLETQEKEKAKAKRPFDELGSGFIGKRPFDEIGSG

FIGKRPFDEIGSGFIGKRPFDELGSGFIGKRPFDELGSGFIGKRPFDEIGSGFIGKRPF
DELGSGFIGKRPFDELGSGFIGKRPFDEIGSGFIGKRPFDEIGSGFIGKRPFDELGSGF
IGKRPFDELGSGFIGKRPFDEIGSGFIGKRPFDELGSGFIGKRPFDELGSGFIGKRPFD
ELGSGFIGKRPFDELGSGFIGKRPFDEIGSGFIGKRPFDEIGSGFIGKRPFDELGSGFI
GKRRPFSELASGLVGKRRPFGELSSGLVGKRPFDELGSGLVGKRPFDELSSGLVGKRSE
NKAVKRPFDELASGLVGKRSTQ

**Octopus bimaculoides**
>XP_014768501.1
MKMWKITNISLVTLLLLHTVFTENSPDKKQLDQLSGKLNRELSLDHLANQLSENIEPLS
NRLIGKRFLDPLASGLIGKRYLDPLASGLLGKRNSDVNTSKNHAGKSQKDGILSQTGKQ
HFDLLDRELIGKRYLDPLASGLIGKRYLDPLASGLIGKRYLDPLASGLLGKRDTHLDSK
SDDYQGTDDNTSNDLAINDAGKRYLDPLASSIIGKRHLDPLASGLIGKRYLDPLASGLI
GKRYLDPLASGLISKRYLDPLASGLIGKRYLDPLASGLIGKRYLDPLASGLIGKRYLDP
LASGLISKRYLDPLASGLIGKRYLDPLASGLIGKRYLDPLASGLIGKRYLDPLASGLIG
KRYLDPLASGLIGKRYLDPLASGLIGKRYLDPLASDLIGKRYLDPLASDLIGKRDVNPN
SSKENSENSRSNVILDPTTNGSAMNKRYLDPLASGIIGKRQNEKLE
