# Supplementary figures and images for: Evolution and Potential Function in Molluscs of Neuropeptide and Receptor Homologues of the Insect Allatostatins
Source: Front Endocrinol (Lausanne). 2021 Sep 29;12:725022. doi: 10.3389/fendo.2021.725022 (PMC8514136; doi:10.3389/fendo.2021.725022)

Supplementary Figure 3

A)

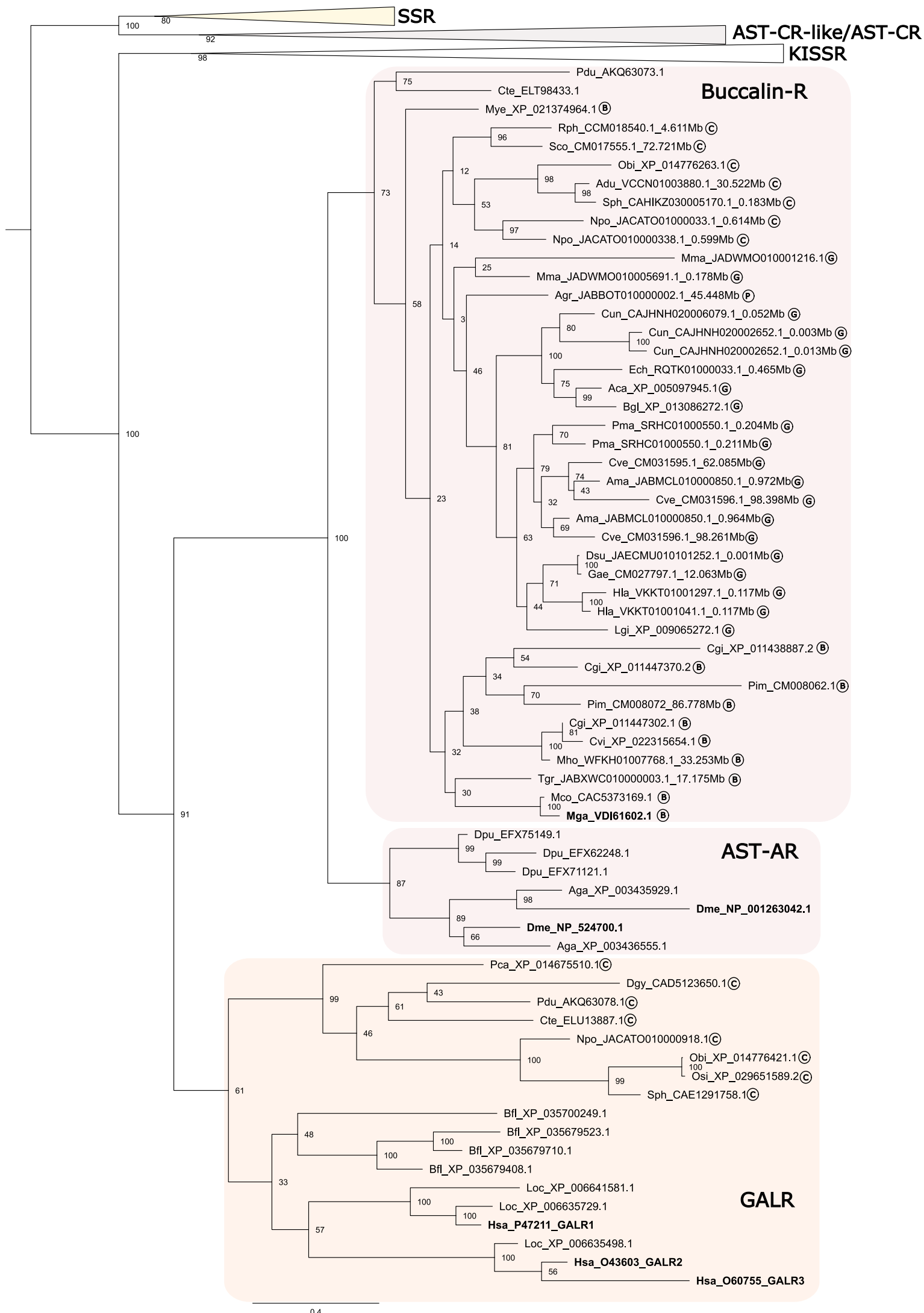

B)

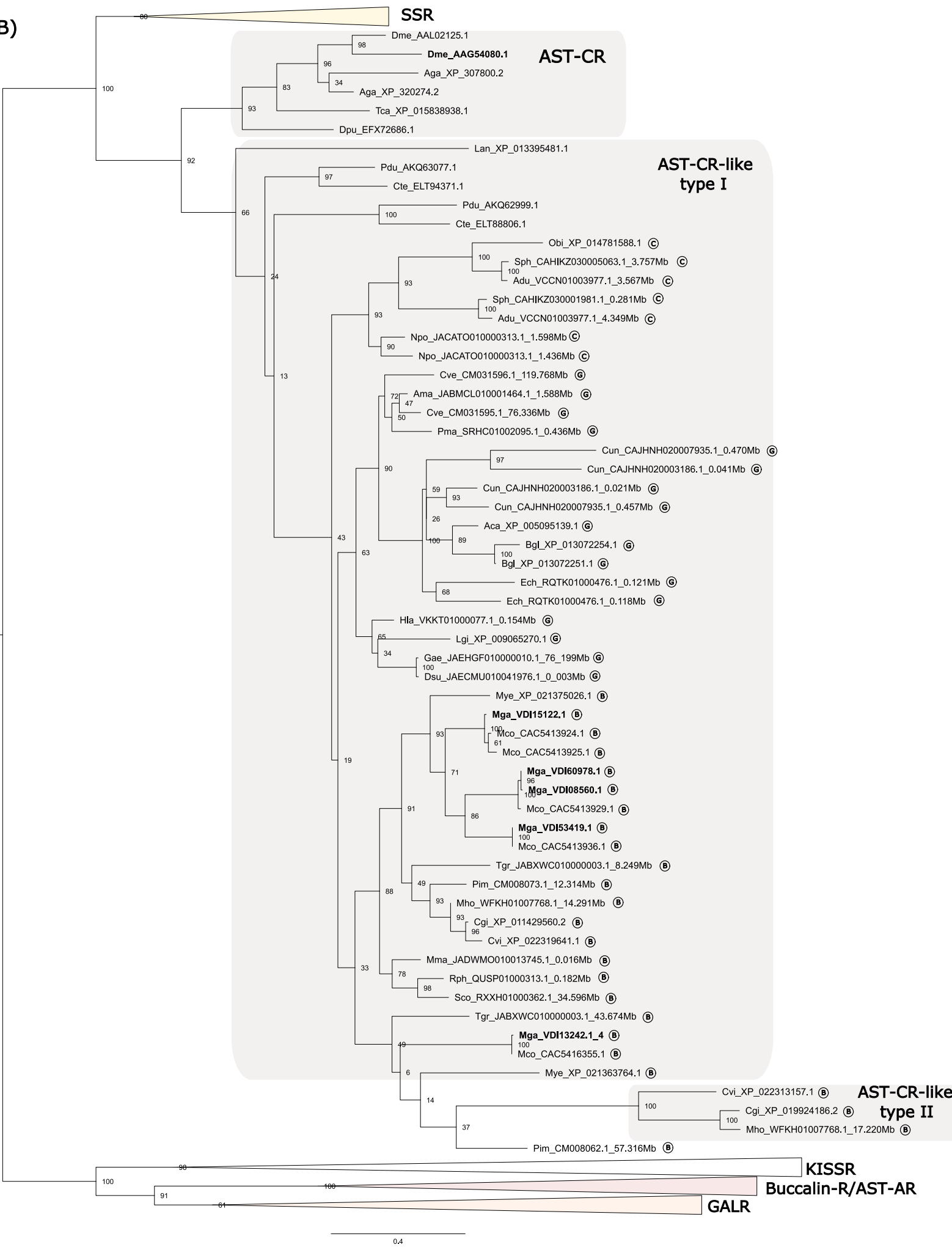

c)

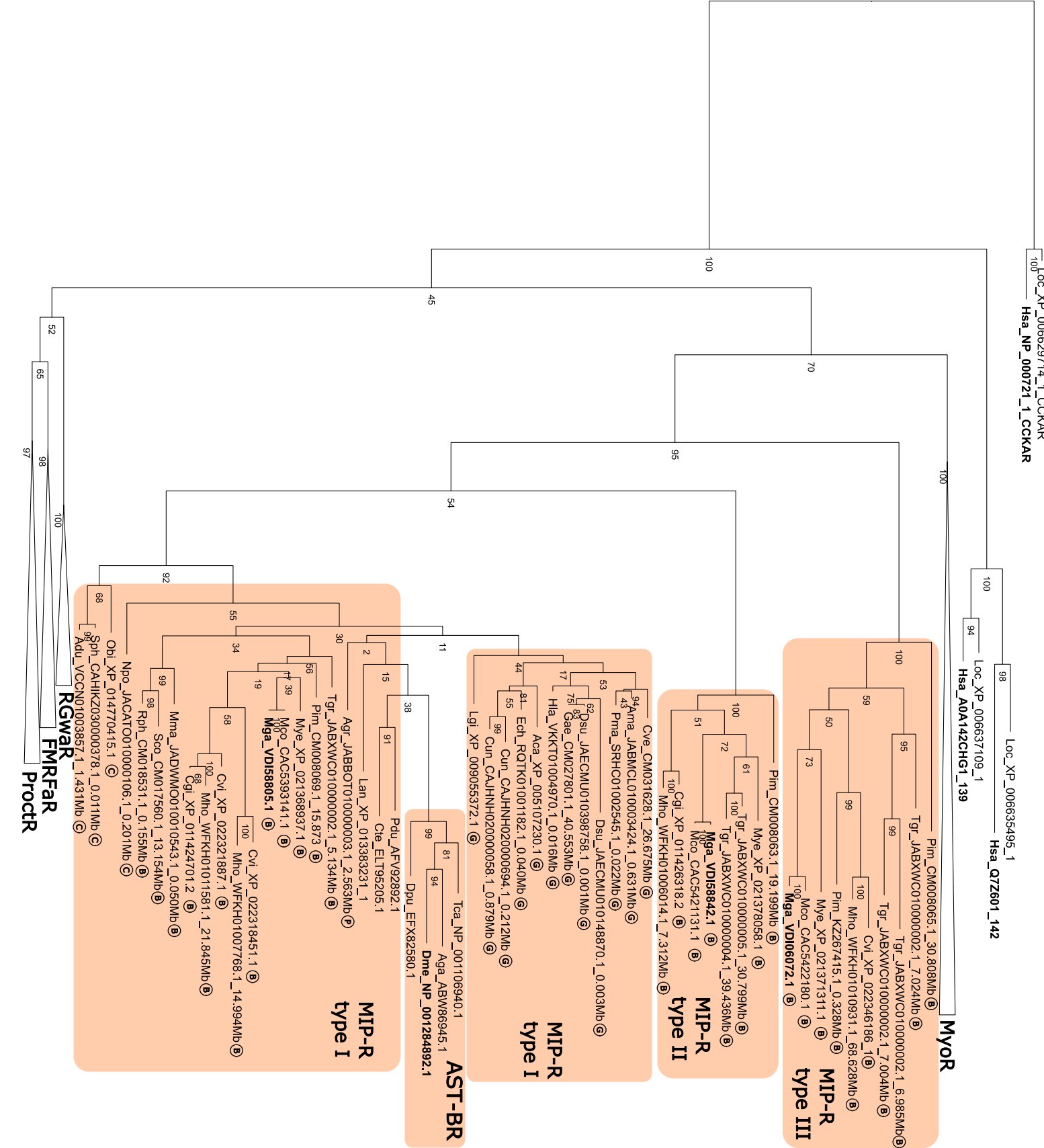

Supplement: Supplementary Figure 3 — Maximum Likelihood (ML) trees of the Molluscan receptors and homologues from other lophotrochozoans, ecdysozoans and deuterostomes. (A) Phylogenetic tree of the Buccalin-R/AST-ARs and AST-CR-like/AST-CRs (Rhodopsin γ family) and (B) Phylogenetic tree of MIP-Rs/AST-BRs (Rhodopsin β family). Trees were built with 100 bootstrap replicates and were performed with an LG model. The Rhodopsin γ family tree was mid-rooted based on the clustering of the sequences and the Rhodopsin β family tree was rooted with the H. sapiens (NP_000721) and L. oculeatus (XP_006629714) cholecystokinin receptor type A (CCKAR). Trees were displayed in FigTreev1.4.4 and edited in the Inkscape programme. Circled letters indicate: B- bivalves; G- gastropods, C- cephalopods; and P- polyplacophore species used in the analysis. The sequences that were retrieved from non-annotated genomes have the putative localization (Mbp) indicated. [file DataSheet_3.pdf]

Supplementary Figure 6

*TLRa*

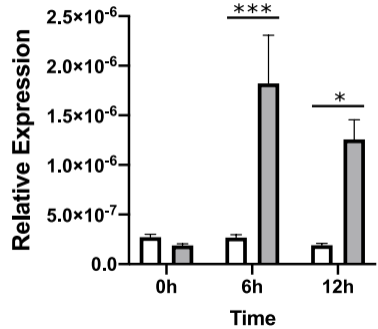

*LYG1*

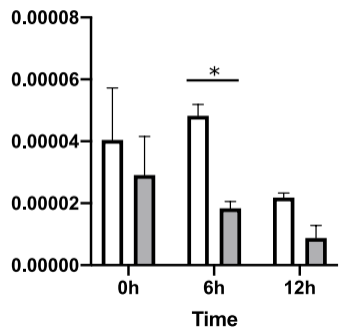

*C345i*

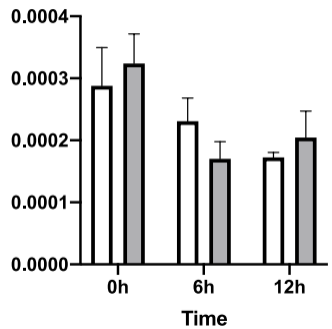

Control  
*Vibrio harveyi*

Supplement: Supplementary Figure 6 — Quantitative expression analysis of TLRa, LYG1 and C3-like in the mantle edge of control and V. harveyi challenged M. galloprovincialis. Mantle samples at 0, 6 and 12 hours post exposure to the pathogen were analysed. The immune genes tested were TLRa (82), LYG1 (83) and C3-like (Peng et al., submitted). Expression levels were normalized using the geometric mean of two reference genes (EF1α and 18S). The results are represented as the mean ± SEM of three (n= 3) biological replicates per group/sampling point. Significant differences (*p< 0.05 and ***p< 0.001) between the control and immune challenged groups of the same sampling period were detected using two-way ANOVA and a Sidak’s multiple comparison test using GraphPad Prism version 8.0.0 software for Mac OS X. [file DataSheet_6.pdf]
