# Supplementary material for: Evolution and Potential Function in Molluscs of Neuropeptide and Receptor Homologues of the Insect Allatostatins
Source: Front Endocrinol (Lausanne). 2021 Sep 29;12:725022. doi: 10.3389/fendo.2021.725022 (PMC8514136; doi:10.3389/fendo.2021.725022)
Supplement: Supplementary Figure 4 — Multiple sequence alignments of Mollusca (bivalve, gastropod, and cephalopod) receptors with orthologues from annelids and D. melanogaster. (A) Buccalin-R/AST-AR, (B) MIP-R/AST-BR and (C) AST-CR-like/AST-CR. Receptor transmembrane domains are annotated within red boxes. Shading denotes amino acid conservation; dark grey represents 80% and black 100% conservation. Other sequence motifs suggested to be important for receptor function the motif DRY in ICL2 between TM3 and TM4 and NSxxNPxxY (where x represents any aa) within TM7 are marked with “*”. The putative disulphide bridge between the ECL1 and ECL2 is annotated and the C-terminal cysteine for potential palmitoylation after TM7 is marked with “#”. The N-terminal N-glycosylation sites conserved across the species are annotated with “.”. The accession number of receptor genes are indicated. [file DataSheet_4.pdf]

Supplementary Figure 4

A)

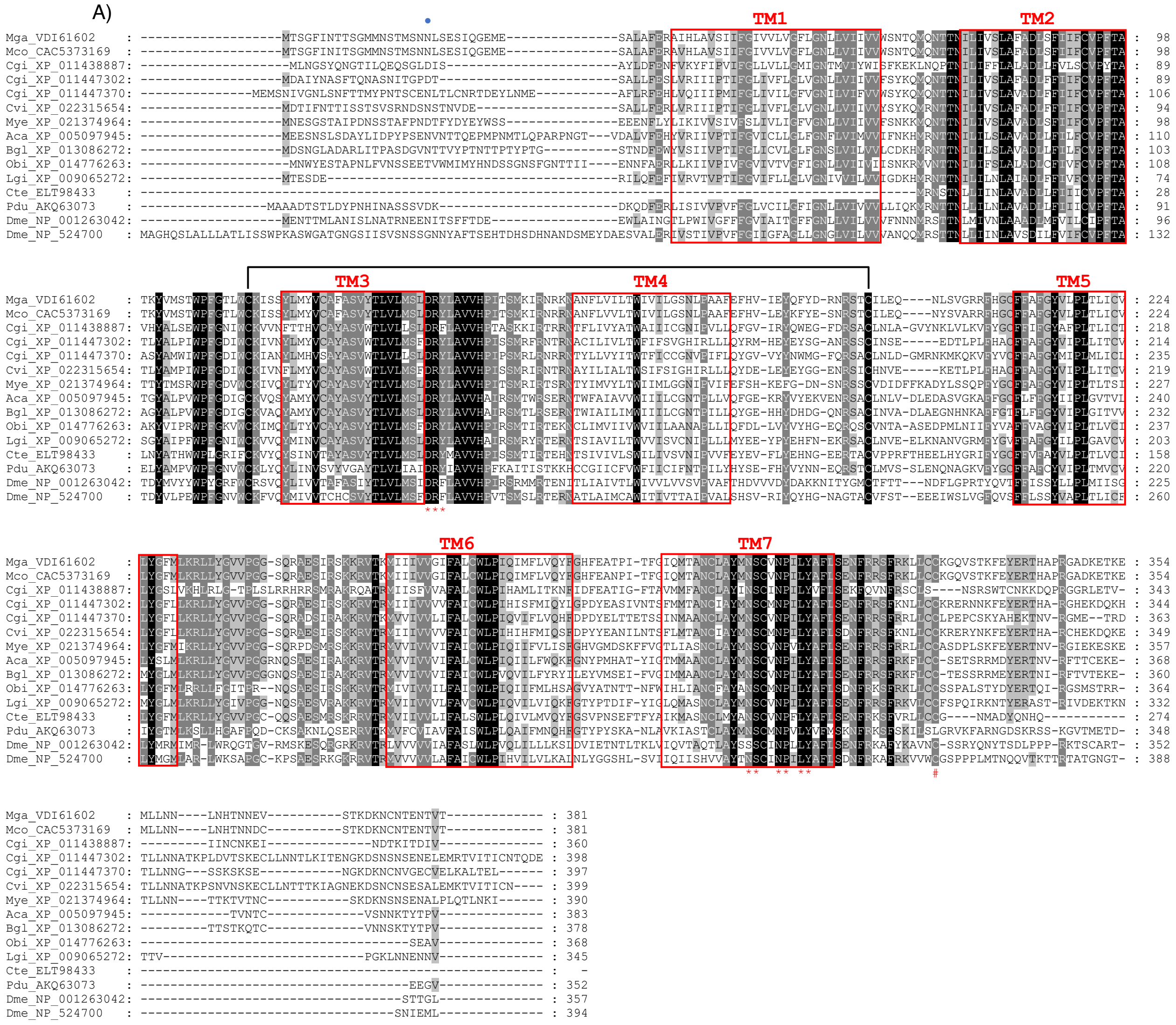

B)

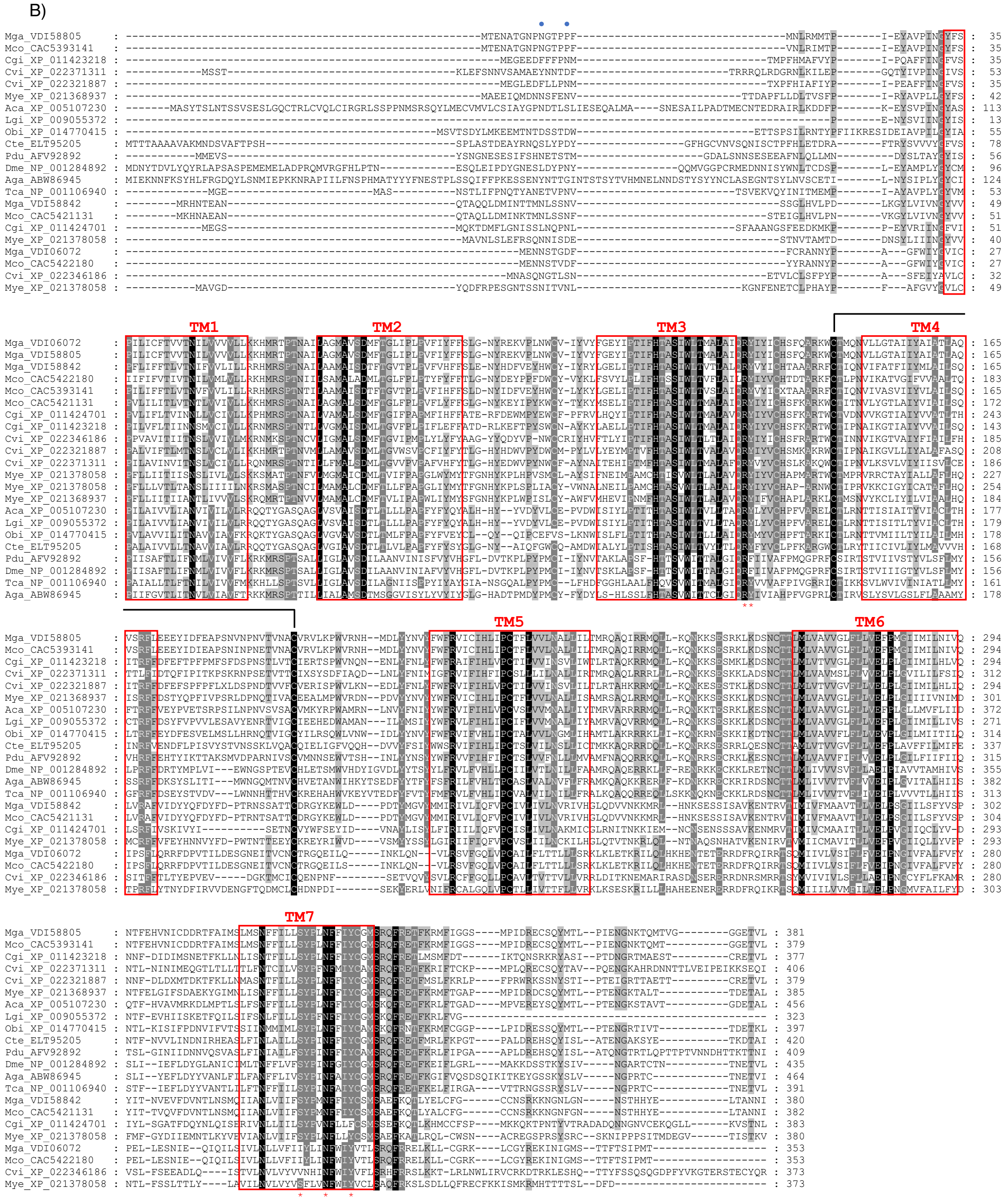

C)

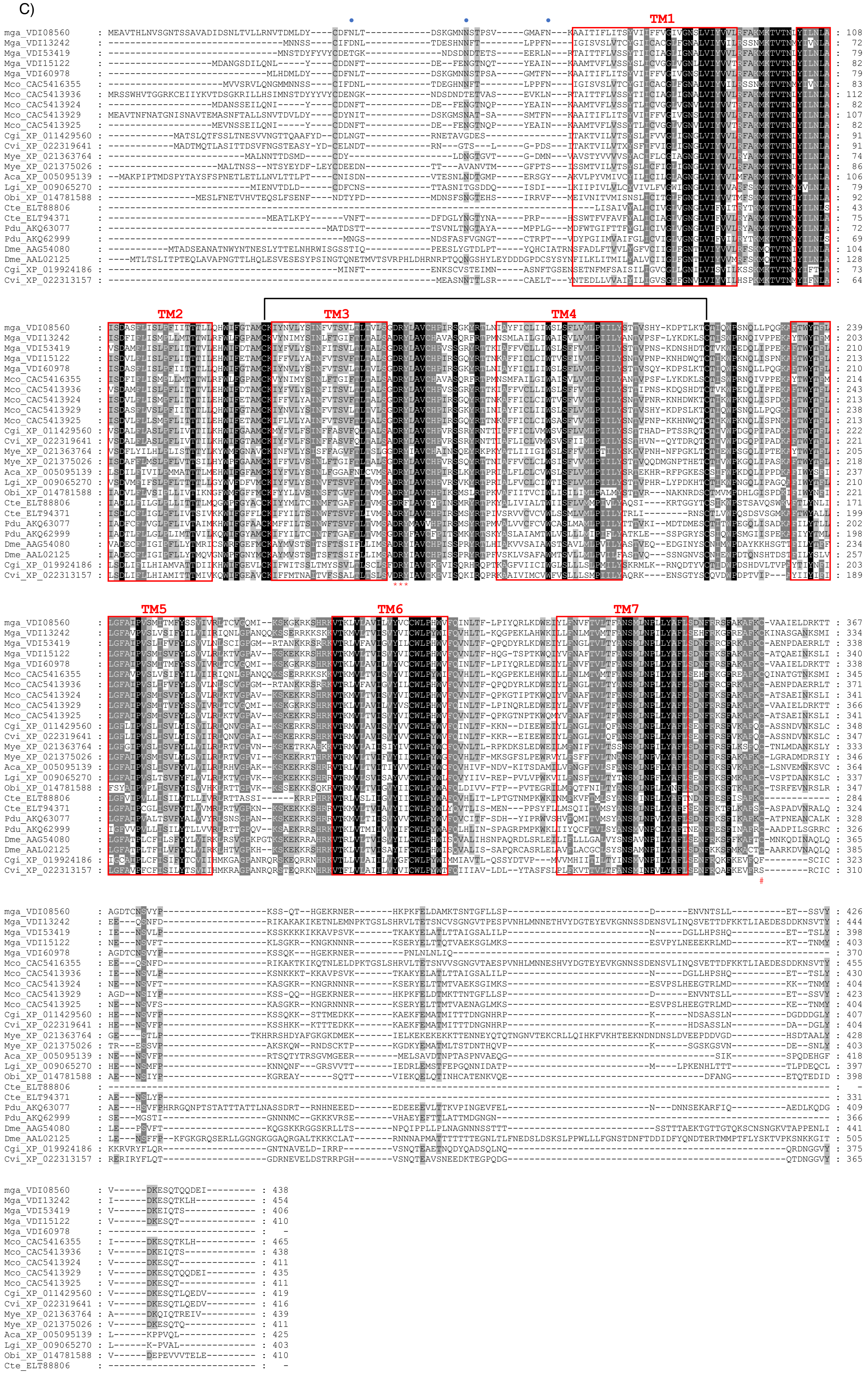

```
Cte_ELT94371     : --------------------------- :   -
Pdu_AKQ63077     : L------DKPTAV-------------- : 416
Pdu_AKQ62999     : --------------------------- :   -
Dme_AAG54080     : ICLSEQQEAFCTTARRGSGAVQQTDL- : 467
Dme_AAL02125     : CAILWPSDKHTEGTKAFILYCFFLSYA : 532
Cgi_XP_019924186 : T------GMLE---------------- : 380
Cvi_XP_022313157 : T------GP------------------ : 368
```
