# Supplementary material for: Evolution and Potential Function in Molluscs of Neuropeptide and Receptor Homologues of the Insect Allatostatins
Source: Front Endocrinol (Lausanne). 2021 Sep 29;12:725022. doi: 10.3389/fendo.2021.725022 (PMC8514136; doi:10.3389/fendo.2021.725022)
Supplement: Supplementary Figure 5 — Transcript abundance (FPKM) of the Buccalin, MIP and AST-CR-like peptide precursors and receptors in mantle edge transcriptomes from control and LPS challenged M. galloprovincialis. Data represents transcript abundance of the control (n= 3) and LPS challenged (n= 3) animals after 12h. The colour grades are indicative of transcript abundance, and the more intense colour indicates higher abundance. FPKM counts were calculated taking into consideration the number of reads, gene length and the transcriptome sequencing depth. The AST-CR (VDI60978.1) transcript highlighted in bold and italics was DE (p-adj < 0.05, log2-fold > 2) between the two conditions. [file DataSheet_5.pdf]

Supplementary Figure 5

		**Accession number**	**Control 1**	**Control 2**	**Control 3**	**LPS 1**	**LPS 2**	**LPS 3**	**Log2-fold**	**P-adj value**
**Buccalin system**	Precursor	VDI48108.1	1,36	1,72	1,49	1,55	1,80	1,93	0,79	0,93
	Receptor	VDI61602.1	0,68	1,41	1,31	1,16	1,17	0,93	-0,43	1,00
**MIP system**	Precursor	VDI26375.1	1,19	1,60	1,69	0,00	1,57	1,31	-0,87	0,99
	Receptor	VDI06072.1	0,00	0,16	0,00	0,00	0,76	1,37	4,46	NA
		VDI58805.1	2,04	2,17	2,28	1,76	1,96	2,00	-0,85	0,72
		VDI58842.1	0,00	0,00	1,70	0,34	1,03	1,53	-0,11	1,00
**AST-C-like system**	Precursor	AST-C	0,00	0,00	0,00	0,00	0,00	0,00	0,00	NA
	Receptor	VDI08560.1	0,00	1,55	0,63	1,68	1,54	0,45	1,10	0,99
		VDI13242.1	0,00	0,00	0,00	0,00	0,00	1,08	4,15	NA
		VDI15122.1	0,00	0,00	1,54	0,00	0,00	0,00	-6,37	NA
		VDI53419.1	0,00	1,50	1,71	1,18	2,03	0,69	0,61	1,00
		VDI60978.1	***1,46***	***0,63***	***1,33***	***0,00***	***0,00***	***0,00***	***-7,04***	***0,04***
