# Supplementary material for: Evolution and Potential Function in Molluscs of Neuropeptide and Receptor Homologues of the Insect Allatostatins
Source: Front Endocrinol (Lausanne). 2021 Sep 29;12:725022. doi: 10.3389/fendo.2021.725022 (PMC8514136; doi:10.3389/fendo.2021.725022)
Supplement: Supplementary Table 2 — Accession numbers of bivalve sequence read archives (SRA) for RPKM analysis. SRA are available from https://www.ncbi.nlm.nih.gov/sra/. [file Table_2.docx]

Supplementary Table 2

|  | **Gills** | **Digestive gland** | **Mantle** | **Haemocytes** | **Muscle** | **Nerve ganglia** |
| --- | --- | --- | --- | --- | --- | --- |
| *M. galloprovincialis* | SRX389466 | SRX3346683 | SRX389462 | SRX4059490 | SRX389464 |  |
| *M. coruscus* | SRX7191260 | SRX7191250 | SRX7191264 | SRX7191262 | SRX7191249 |  |
| *C. gigas* | SRX093414 | SRX093412 | SRX093411  SRX093415 | SRX093417 | SRX093416 |  |
| *M. yessoensis* | SRX2238797  SRX2250258  SRX2238798 | SRX2238801  SRX2251047  SRX2238802 | SRX2238803  SRX2250257  SRX2238804 | SRX2250259  SRX2238800 | SRX2251056  SRX2238806  SRX2238806  SRX2238807  SRX2251049  SRX2279546 | SRX3500764 |
